# A near telomere-to-telomere phased genome assembly and annotation for the Australian central bearded dragon *Pogona vitticeps*

**DOI:** 10.1093/gigascience/giaf085

**Published:** 2025-08-19

**Authors:** Hardip R Patel, Kirat Alreja, Andre L M Reis, J King Chang, Zahra A Chew, Hyungtaek Jung, Jillian M Hammond, Ira W Deveson, Aurora Ruiz-Herrera, Laia Marin-Gual, Clare E Holleley, Xiuwen Zhang, Nicholas C Lister, Sarah Whiteley, Lei Xiong, Duminda S B Dissanayake, Paul D Waters, Arthur Georges

**Affiliations:** National Centre for Indigenous Genomics, John Curtin School of Medical Research, Australian National University, Canberra, ACT 2601, Australia; National Centre for Indigenous Genomics, John Curtin School of Medical Research, Australian National University, Canberra, ACT 2601, Australia; Genomics and Inherited Disease Program, Garvan Institute of Medical Research, Sydney, NSW 2010, Australia; Centre for Population Genomics, Garvan Institute of Medical Research and Murdoch Children’s Research Institute, Darlinghurst, NSW 2010, Australia; Faculty of Medicine, University of New South Wales, Sydney, NSW 2052, Australia; Faculty of Science, School of Biotechnology and Biomolecular Science, University of New South Wales, Sydney, NSW 2052, Australia; National Centre for Indigenous Genomics, John Curtin School of Medical Research, Australian National University, Canberra, ACT 2601, Australia; National Centre for Indigenous Genomics, John Curtin School of Medical Research, Australian National University, Canberra, ACT 2601, Australia; Genomics and Inherited Disease Program, Garvan Institute of Medical Research, Sydney, NSW 2010, Australia; Centre for Population Genomics, Garvan Institute of Medical Research and Murdoch Children’s Research Institute, Darlinghurst, NSW 2010, Australia; Genomics and Inherited Disease Program, Garvan Institute of Medical Research, Sydney, NSW 2010, Australia; Centre for Population Genomics, Garvan Institute of Medical Research and Murdoch Children’s Research Institute, Darlinghurst, NSW 2010, Australia; Faculty of Medicine, University of New South Wales, Sydney, NSW 2052, Australia; Department of Cel.lular Biology, Physiology and Immunology, Universitat Autònoma de Barcelona (UAB), Cerdanyola del Vallès 08193, Spain; Genome Integrity and Instability Group, Institut de Biotecnologia i Biomedicina, Universitat Autònoma de Barcelona (UAB), Cerdanyola del Vallès 08193, Spain; Department of Cel.lular Biology, Physiology and Immunology, Universitat Autònoma de Barcelona (UAB), Cerdanyola del Vallès 08193, Spain; Genome Integrity and Instability Group, Institut de Biotecnologia i Biomedicina, Universitat Autònoma de Barcelona (UAB), Cerdanyola del Vallès 08193, Spain; Australian National Wildlife Collection, CSIRO, Canberra, ACT 2601, Australia; Institute for Applied Ecology, University of Canberra,Canberra, ACT 2617, Australia; Faculty of Science, School of Biotechnology and Biomolecular Science, University of New South Wales, Sydney, NSW 2052, Australia; Institute for Applied Ecology, University of Canberra,Canberra, ACT 2617, Australia; Institute for Applied Ecology, University of Canberra,Canberra, ACT 2617, Australia; Department of Biochemistry and Molecular Biology, School of Basic Medicine, Wannan Medical College, Wuhu 241001, Anhui, China; Institute for Applied Ecology, University of Canberra,Canberra, ACT 2617, Australia; Faculty of Science, School of Biotechnology and Biomolecular Science, University of New South Wales, Sydney, NSW 2052, Australia; Institute for Applied Ecology, University of Canberra, Canberra, ACT 2617, Australia

**Keywords:** Squamata, Agamidae, lizard, AusARG, sex determination

## Abstract

**Background:**

The central bearded dragon (*Pogona vitticeps*) is widely distributed in central eastern Australia and adapts readily to captivity. Among other attributes, it is distinctive because it undergoes sex reversal from ZZ genotypic males to phenotypic females at high incubation temperatures. Here, we report an annotated near telomere-to-telomere phased assembly of the genome of a female ZW central bearded dragon.

**Results:**

Genome assembly length is 1.75 Gbp with a scaffold N50 of 266.2 Mbp, N90 of 28.1 Mbp, 26 gaps, and 42.2% GC content. Most (99.6%) of the reference assembly is scaffolded into 6 macrochromosomes and 10 microchromosomes, including the Z and W microchromosomes, corresponding to the karyotype. The genome assembly exceeds standard recommended by the Earth Biogenome Project (6CQ40): 0.003% collapsed sequence, 0.03% false expansions, 99.8% *k*-mer completeness, 97.9% complete single-copy BUSCO genes, and an average of 93.5% of transcriptome data mappable back to the genome assembly. The mitochondrial genome (16,731 bp) and the model ribosomal DNA repeat unit (length 9.5 Kbp) were assembled. Male vertebrate sex genes *Amh* and *Amhr2* were discovered as copies in the small non-recombining region of the Z chromosome, absent from the W chromosome. This, coupled with the prior discovery of differential Z and W transcriptional isoform composition arising from pseudo-autosomal sex gene *Nr5a1*, suggests that complex interactions between these genes, their autosomal copies, and their resultant transcription factors and intermediaries determine sex in the bearded dragon.

**Conclusion:**

This high-quality assembly will serve as a resource to enable and accelerate research into the unusual reproductive attributes of this species and for comparative studies across the Agamidae and reptiles more generally.

## Graphical Abstract

**Graphical Abstract:**
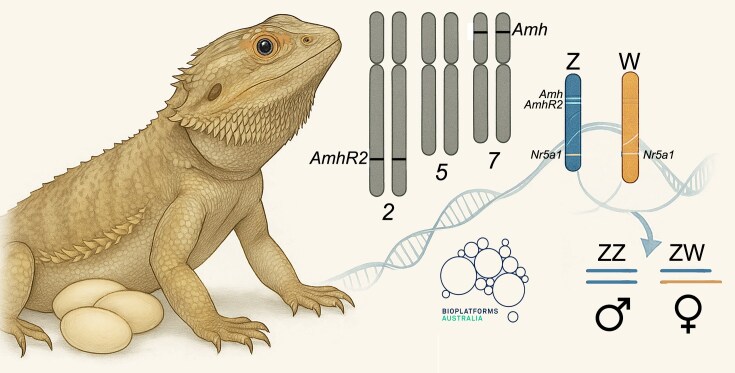
Assembly of a ZW female of the Central Bearded Dragon.

## Species Taxonomy

Eukaryota; Animalia; Chordata; Reptilia; Squamata; Iguania; Agamidae; Amphibolurinae; *Pogona; Pogona vitticeps* (NCBI:txid103695).

## Introduction

**Figure 1:**
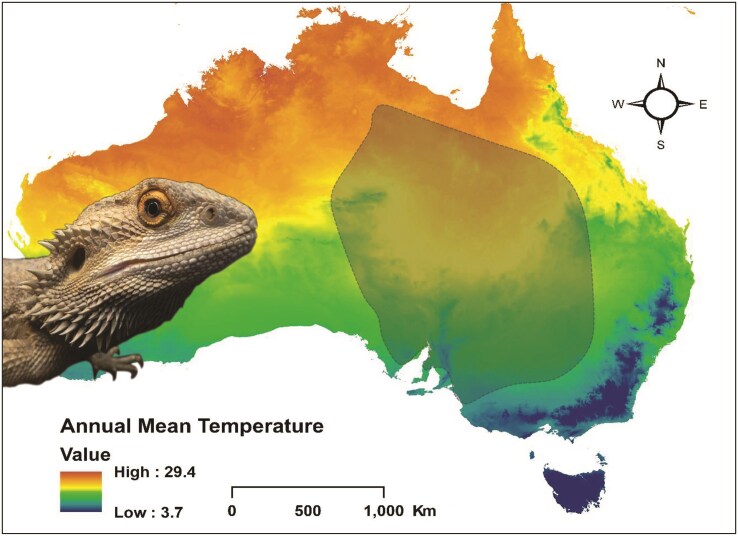
The central bearded dragon *P. vitticeps* and the distribution of the species based on records from Australian museums (via Atlas of Living Australia https://www.ala.org.au/).

The family Agamidae, commonly known as dragon lizards, is a diverse group of lizards found in Africa, Asia, Australia, the Western Pacific, and warmer regions of southern Europe. The Agamidae family is well represented in Australia, in part because of their successful radiation in response to the progressive aridification of the Australian continent during the Pleistocene. New species are continually being described, but on recent count, they comprise 81 species in 15 genera [1] that occupy a very wide array of habitats ranging from the inland deserts to the mesic habitats of the coast and the Australian Alps below the treeline. The family includes some iconic species, such as the thorny devil *Moloch horridus* and the frillneck lizard *Chlamydosaurus kingii*. Less spectacular perhaps is the central bearded dragon *Pogona vitticeps*, a widely distributed species of Amphibolurine dragon common in central eastern Australia (Fig. 1). The bearded dragon feeds on insects and other invertebrates, but a substantial component of the diet of adults is vegetable matter. It lives in the dry sclerophyll forests and woodlands in the southeast of its range, mallee and arid acacia scrublands further north and west, and the sandy deserts of the interior. Semi-arboreal, the species often perches on fallen timber and tree branches, only to retreat to ground cover when disturbed.

Central bearded dragons adapt readily to captivity, lay large clutches of eggs several times per season, and are commonly kept as a pet in Europe, Asia, and North America. These attributes also increase its value as a popular reptile research model in a range of disciplines [2–7]. Central bearded dragons are a particularly compelling model species for sex determination because they display temperature-induced sex reversal in the laboratory and in the wild [8–10]. The sex chromosomes of central bearded dragons are poorly differentiated morphologically. They exhibit female heterogamety (ZZ/ZW sex chromosome system, [11]) with 6 macrochromosome pairs and 10 microchromosome pairs [12] that include the sex microchromosome pair [11]. Bacterial artificial chromosome (BAC) sequences have been physically mapped uniquely to each of the chromosomes [13, 14].

Sex determination in this species is particularly subtle until now, with no substantial difference between the Z and W chromosome gene content or single-copy sequence [15]. The developmental program initiated by chromosomal sex determination can be reversed by high incubation temperature, allowing for investigations of environmental influences on fundamental developmental processes. Research in these areas of interest will be greatly facilitated by applying modern sequencing technologies to generate a high-quality draft genome assembly for the central bearded dragon. The ability to generate telomere-to-telomere (T2T) assemblies of the sex chromosomes and identify the non-recombining regions within which lies any master sex determining gene will greatly narrow the field of candidate sex-determining genes in species with chromosomal sex determination. Furthermore, the disaggregation of the Z and W sex chromosome haplotypes (phasing) will allow comparisons of the Z and W sequences to gauge putative loss or difference in function of key sex gene candidates.

In this article, we present a draft annotated near-T2T phased assembly of the genome of the Australian central bearded dragon as a resource to enable and accelerate research into the unusual reproductive attributes of this species and for comparative studies across the Agamidae and reptiles more generally. This is a vastly improved assembly in comparison with an earlier assembly based on Illumina short-read technology published in 2015 [16].

## Materials and Methods

### Sample collection

DNA samples were obtained from a blood sample taken from a single female *P. vitticeps* (RadMum, UCID Pit_001,003,342,236, NCBI:txid103695) collected on 15 March 2011 on a road verge 62 km west of Eulo on Adventure Way, Queensland (GPS −28.099000 144.433000). It was verified as a ZW female using sex-linked PCR markers [9].

An additional 3 adult individuals were sampled to provide tissues (brain, heart, kidney, liver, lung, skeletal muscle, testes, ovary), complemented by embryonic brain and gonad, for transcriptomics (Supplementary Table S2).

### Extraction and sequencing

**Figure 2:**
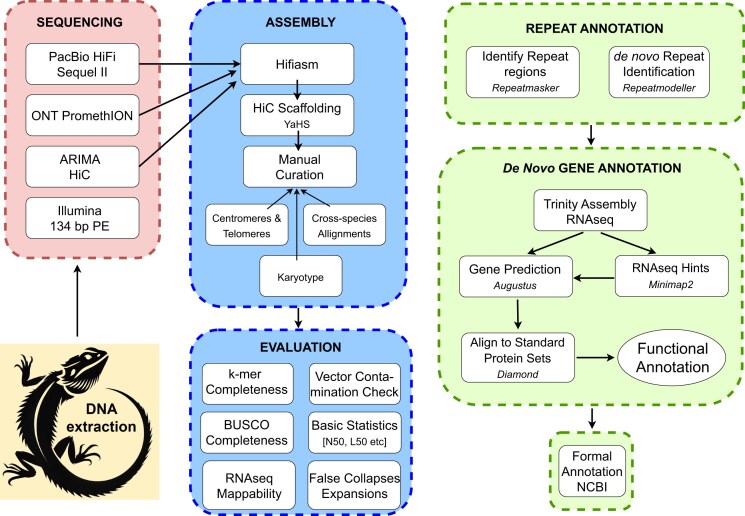
Schematic overview of workflow for sequencing, assembly, and annotation of the genome of the central bearded dragon *P. vitticeps*. Target: Earth Biogenomes Project standard 6CQ40 [17]. Illumina 134-bp PE reads (Supplementary Table S6) were not used directly in the assembly, but for quality assessment of the genome. Quality control workflow not shown. Repeat annotation was undertaken with Repeatmasker (4.1.2-p1, [18]). Refer to Supplementary Table S1 for software used in this project.

We generated sequencing data using 3 platforms—Pacific Biosciences (PacBio) HiFi, Oxford Nanopore Technologies (ONT) ultralong reads, and HiC generated using the Arima Genomics protocols (Fig. 2). Illumina short-read DNA data were previously generated [16]. Transcriptome data were generated using the Illumina platform. All sequence data generated in this study are available from the National Center for Biotechnology Information (NCBI) SRA under BioProject ID PRJNA1252275.

#### PacBio HiFi

Genomic DNA was extracted from blood of the focal ZW individual by PacBio Asia (Singapore) and sequenced using 2 flow cells on a PacBio Sequel II (Supplementary Table S3). HiFi data were processed using *cutadapt* (v3.7, parameters: –anywhere –error-rate 0.1 –overlap 25 –match-read-wildcards –revcomp –discard-trimmed) to remove reads containing PacBio primers and adapter sequences. This step removes putative chimeric sequences.

#### ONT PromethION

Genomic DNA was extracted from blood of the focal ZW individual (Supplementary Table S4) using the salting-out procedure [19] and spooled to enrich for high molecular weight DNA. DNA was shipped to the Garvan Institute of Medical Research in Sydney. Library preparation was performed with 3 µg DNA as input, using the SQK-LSK109 kit (Oxford Nanopore Technologies) and sequenced across 4× promethION (FLO-PRO002) flow cells, with washes (EXP-WSH004) performed when sequencing dropped.

A second extraction was performed on 10 µL of blood using the Circulomics UHMW extraction kit, following the “Nucleated blood” protocol, obtaining approximately 60 µg of ultra-high molecular weight DNA. Library preparation was then performed using a prerelease version of the SQK-ULK001 kit from Oxford Nanopore Technologies, which uses the RAP adapter. The library was then loaded onto 1 promethION (FLO-PRO002) flow cell with washes (EXP-WSH004) performed at 24 and 48 hours to increase output.

ONT basecalling was performed using the *buttery-eel* (v0.4.2+dorado7.2.13, [20], parameters: –config dna_r9.4.1_450bps_hac_prom.cfg –detect_mid_strand_adapter –trim_adapters –detect_adapter –do_read_splitting –qscore 7). Parameters were chosen to remove reads with average quality value score <7, remove adapters at 5′ or 3′ ends of sequence, and split reads if adapters were in the middle of the read.

#### HiC

A blood sample from the focal ZW individual (Supplementary Table S5) was used for HiC. Blood sample was processed by the Biomolecular Resource Facility (BRF) at the Australian National University using the Arima HiC 2.0 kit for library preparation and sequencing with 2 flow cells on an Illumina NovaSeq 6000 NovaSeq 6000, S1 300 cycles kit 2 × 150 bp.

#### RNA

Total RNA was extracted from adult brain, liver, heart, and ovary/testis (Supplementary Table S2) by the Garvan Institute of Medical Research. Tissue extracts were homogenized using the T10 Basic ULTRA-TURRAX Homogenizer (IKA) and extracted using TRIzol reagent following the manufacturer’s instructions, purifying with an isopropanol precipitation. In total, 75-bp single-end reads were generated for recent samples on the Illumina NextSeq 500 platform at the Ramaciotti Centre for Genomics (UNSW, Sydney, Australia). Some earlier samples generated 100-bp PE reads.

RNA sequencing (RNA-seq) from 3 embryonic gonads was sourced from Whiteley et al. [21], and RNA-seq from 3 embyronic brains was sourced from Whiteley et al. [22] and Wagner et al. [23] (Supplementary Table S2).

### Assembly

All data analyses were performed on the high-performance computing facility, Gadi, hosted by Australia’s National Computational Infrastructure (NCI, https://nci.org.au). Scripts are available at https://github.com/kango2/ausarg.

#### Primary genome assembly

PacBio HiFi, ONT, and HiC sequence data were used to generate interim haplotype assemblies and an interim pseudo-haplotype (=consensus haplotype) assembly using *hifiasm* (v0.19.8, [24, 25], RRID:SCR_021069, default parameters). HiC data were aligned to the interim pseudo-haplotype and haplotype assembly using the *Arima Genomics alignment pipeline* [26] following the user guide for scaffolding and assessing the accuracy of assembly. HiC read alignments were processed using *YaHS* (v1.1, [27], RRID:SCR_022965, parameters: -r 10,000,20,000, 50,000,100,000,200,000,500,000,1,000,000,1,500,000 –no-contig-ec -e GATC,GANTC,CTNAG,TTAA) to generate scaffolds. Range resolution parameter (-r) in *YaHS* was restricted to 1,500,000 to ensure separation of microchromosomes into individual scaffolds. Contig correction was disabled to maintain the original contig structure produced by *hifiasm*.

HiC contact maps were processed and visualized using *Juicer* (v1.5, [28], RRID:SCR_017226). Read depth, GC content, and telomere locations for *YaHS* scaffolds >1 Mbp length were visually inspected. One scaffold in the pseudo-haplotype assembly contained an internal telomeric repeat and contact pattern of a misjoin, owing to incorrect contig assembly by *hifiasm*. A similar error was observed for 1 scaffold in haplotype 2 as a result of scaffolding error (Supplementary Fig. S2). YaHS was rerun without –no-contig-ec parameter, which is the default behavior that fixed these errors.

#### Reference genome assembly

The karyotype was obtained from Witten [12] and Ezaz et al. [11] as a guide for the expected number of chromosomes for final T2T assembly. A reference assembly was *generated* by choosing the best chromosome scaffolds from 1 of the 2 haplotype assemblies. The basis for selection was as follows for scaffolds >1 Mbp in size. If the scaffold of haplotype 1 had both ends represented by telomeric sequence and the corresponding scaffold of haplotype 2 had only 1 end represented by a telomeric sequence, then the scaffold for haplotype 1 was chosen for the reference assembly and vice versa. If both the scaffolds for haplotype 1 and haplotype 2 contained telomeric sequence at both ends, then the scaffold with the fewest gaps was chosen for the reference assembly. If both T2T haplotypes had the same number of gaps, then the longest scaffold was selected for the reference assembly. If both haplotypes were equal in telomere presence, number of gaps, and length, haplotype 1 sequence was chosen for the reference assembly. The Z- and W-specific scaffolds were added to the reference assembly. All scaffolds <1 Mbp were drawn from haplotype 1 for the reference assembly.

#### Chromosome assignments

BAC clones were previously used for generating the physical map for *P. vitticeps* [13, 14]. BAC end sequences (*n* = 273) corresponding to 137 clones were downloaded from the NCBI GSS database. These sequences were aligned to the reference genome using *minimap2* (parameters: -x asm20 –secondary-no) to identify their locations in the reference genome. We also mapped the sex-linked sequence represented by 3,288-bp clone C1 of Quinn et al. [29] (Genbank accession EU938138) generated by walking out from a sex-linked 50-bp AFLP Pvi72W marker (Genbank accession ED982907) identified by Quinn et al. [10] to confirm the assignment of a scaffold to the non-recombining region of the W chromosome (scaffold 17).

#### Read depth and GC content calculations

PacBio HiFi (parameter: -x map-pb) and ONT (parameter: -x map-ont) sequence data were aligned to the scaffold assembly using *minimap2* (v2.17, [30]). Similarly, Illumina sequence data were aligned to the assembly using *bwa-mem2* (v2.2.1, [31]) using default parameters. Resulting alignment files were sorted and indexed for efficient access using *samtools* (v1.19, [32]). Read depth in non-overlapping sliding windows of 10 Kbp was calculated using the *samtools bedcov* command. GC content in non-overlapping sliding windows of 10 Kbp was calculated using *calculateGC.py* script.

#### Telomere repeats

*Tandem Repeat Finder* (*TRF*) (v4.09.1, [33], parameters: 2 7 7 80 10 500 6 -l 10 -d -h) was used to detect all repeats up to 6 bp length. TRF output was processed using the *processtrftelo.py* script to identify regions >600 bp that contained a conserved vertebrate telomeric repeat motif (TTAGGG). These regions were labeled as potential telomeres.

#### Centromere annotations

Enrichment of satellite repeats, increased interchromosomal HiC contacts [34], and reduced recombination typically mark centromeric regions. To identify satellite repeats, we followed the procedure described by Zhang et al. [35] with some modifications. Briefly, we counted 101-mers occurring 20 times or more with *k*-mer counter *KMC* (v3.2.4, [36], parameters: k = 101, ci = 20, -cs = 100,000). Satellite Repeat Finder (*SRF*, [35], commit id e54ca8c) was used to identify putative satellite repeats using those *k*-mers. Identified repeat units were elongated up to 1,000 bp if they were <1,000 bp, and all-versus-all alignments were performed using *minimap2* to group repeats into classes based on their sequence similarity. The reference genome was aligned to the identified repeat units using *minimap2* ([30] Parameters: –c –N1000000 –f1000 –r100,100 <(srfutils.js enlong srf.fa)). Note that repeat units <200 bp were extended to 200 bp before alignments using the srfutils.js utility in *SRF*. Alignments were processed using the *srfprocess.R* script to merge consecutive alignments to the same repeat unit separated by <10 bp. All regions >100 bp long and 10% of the repeat unit length were retained for further analysis. If a genomic region overlapped multiple repeat classes, the longer region with its repeat class was chosen as a set of the putative satellite repeat region with corresponding repeat class.

HiC interchromosomal interactions were examined and quantified for their association with centromeres. HiC data were mapped against the reference genome using the *GEM mapper* (v3.6.1, [37]) from *TADbit* (v1.0.1, [38]). Reads were iteratively mapped using windows from 15 to 75 bp in 5-bp steps. Possible artifacts were then removed, including “self-circle,” “dangling-end,” “error,” “extra dangling-end,” “too short,” “too large,” “duplicated,” and “random breaks.” Binning and data normalization were conducted using an in-house script that imports the “HiC_data” module of *TADbit* to bin unique reads into a square matrix of 50 Kbp. A 500-Kbp matrix was created and subsequently processed with *HiCExplorer* (v3.7, [39], RRID:SCR_022111). Both 50-Kbp and 500-Kbp matrices were corrected with iterative correction and eigenvector (ICE) decomposition and normalized to a total of 100,000,000 interaction counts by scaling the sum of all interactions within the matrix. Normalized matrices were then plotted at a 500-Kbp resolution using *HiCExplorer*. The normalized 50-Kbp matrix was transformed into a GInteraction table using *HiCExplore*r, which includes interaction values between all genomic bins. Interchromosomal interactions were log-transformed and normalized to obtain *z*-score values for each chromosome and genomic bin, as previously described [40, 41]. The *z*-score values were plotted with ggplot2 as points, and the LOESS method (span = 0.4, [42]) was used for the best-fit line.

For measuring heterozygosity changes across the genome, each haplotype sequence was aligned to the reference genome using *minimap2* (parameters: -x asm5 –cs –K 1000 M). Resulting alignments were processed using *paftools.js call* to identify variant sites. Since one of the haplotype sequences is the reference sequence, all variable sites are considered heterozygous sites. Heterozygous variant site counts in 50-Kbp windows were counted and plotted using *ggplot2*. LOESS smoothing (span = 0.5) was applied for the best-fit line.

#### Sex chromosome identification

The putative Z and W scaffolds will have half the read depth of the autosomal scaffolds in a ZW individual. Scaffolds >1 Mbp long were examined for median read depths in 10-Kbp windows. Sex-specific Z and W scaffolds were identified by having approximately half the median read depth of autosomes and the pseudo-autosomal region (PAR) in the sequenced ZW individual. The PAR scaffold was identified by homology with a known Z chromosome sequence.

#### HiC analysis for sex chromosome differences in contact maps

HiC reads were quality-trimmed using *Trimmomatic* v0.39 [43] (RRID:SCR_011848) to remove adapter sequences and low-quality reads. The trimmed reads of HiC data and Illumina DNA sequence data were aligned to both genome haplotypes using *BWA-mem* (v0.7.17). PacBio data were aligned to both genome haplotypes using *minimap2* v2.28. Resulting BAM files were merged and coordinate-sorted using *SAMtools* v1.19.2 (RRID:SCR_002105). Variant calling for each haplotype was performed using Illumina BAM files with *FreeBayes* v1.3.8 to generate VCF files. These VCF files were normalized using *BCFtools* v1.14, then compressed using *bgzip* from *HTSlib* v1.20. Phasing of VCF files was then conducted using *WhatsHap* v2.3 to resolve haplotype-specific information across the dataset, using genome haplotypes, normalized VCF files, and PacBio BAM files as inputs. Phased VCF files were then used to phase the mapped HiC reads.

#### Mitochondria genome assembly

PacBio HiFi and ONT sequences were aligned to a *P. vitticeps* reference (NCBI Accession: NC_006,922, [44]) using *minimap2* (RRID:SCR_018550, parameters: –map-pb or –map-ont) to search for mitochondrial reads. Alignments were processed to identify reads <20 Kbp and aligned residues >5 Kbp. No PacBio HiFi reads were identified using this filter. ONT reads were assembled using *flye* (v2.9.3, parameters: – iterations 2, [45]) to generate a mitochondrial genome sequence. The output assembly sequence was processed using *MitoHiFi* (v2.9.5, [46], RRID:SCR_026369) to adjust the start coordinate and obtain annotations. Illumina data were aligned to the reference genome, including the mitochondrial sequence, using *bwa-mem2* (v2.2.1, parameters: -Y -K 100,000,000). Alignments were processed using *bcftools* (v1.21, parameters: mpileup –min-MQ 30 -r –adjust-MQ –redo-BAQ –fasta-ref reference.fasta aligned.bam | bcftools call -mv -Ov –ploidy 1 >out.vcf) to call variants on the mitochondria sequence.

### Assembly evaluation

The assembly was evaluated against criteria established by the Earth Biogenomes Project (EBP, https://www.earthbiogenome.org/report-on-assembly-standards, version 6CQ40, [17])—namely, percentage of collapsed sequence, percentage of false expansions, *k*-mer completeness, complete single-copy BUSCO genes, and average percentage of transcriptome data mappable to the genome assembly and contaminations (Fig. 2).

#### K-mer completeness and per base error rate estimation

Illumina sequence data were trimmed for adapters and low-quality reads using *Trimmomatic* (v0.39, [43], parameters: ILLUMINACLIP:TruSeq3-PE.fa":2:30:10:2:True LEADING:3 TRAILING:3 SLIDINGWINDOW:4:20 MINLEN:36). Resultant paired-end sequences were used to generate the *k*-mer database using *meryl* (v1.4.1, [47]). *Merqury* (v1.3, [47]) was used with the *meryl k*-mer database to evaluate assembly *k*-mer completeness and estimate per base error rate of pseudo-haplotype and individual haplotype assemblies.

#### False expansions and collapses

Putative false expansion and collapse metrics were calculated using the *Inspector* (v1.2, [48], default parameters) and PacBio HiFi data.

#### Contamination check

Vector contamination was assessed using *VecScreen*-defined parameters for *BLAST* (v2.14.1, [49], parameters: -task blastn -reward 1 -penalty -5 -gapopen 3 -gapextend 3 -dust yes -soft_masking true -evalue 700 -searchsp 1750000000000) and the *UniVec* database (accessed 18 June 2024).

#### Gene completeness evaluation

*BUSCO* (v5.4.7, [50], RRID:SCR_015008) was run using the *sauropsida_odb10* library in offline mode to assess completeness metrics for conserved genes. BUSCO synteny plots were created with *ChromSyn* (v1.3.0, [51]).

#### RNA-seq mapping rate

RNA-seq data from multiple tissues (Supplementary Table S2) were aligned to the assembly using *subread-align* (v2.0.6, parameters: -n 150 [52]) to calculate the percentage of mapped fragments for evaluating the RNA-seq mapping rate. We chose –n 150 to sample all possible seeds for alignments because of high heterozygosity observed for the species. We did not have RNA-seq data for the focal individual used for the genome assembly.

### Annotation

#### Repeat annotation

*RepeatModeler* (v2.0.4, [53], RRID:SCR_015027, parameters: -engine ncbi) was used to identify and classify repetitive DNA elements in the genome. Subsequently, *RepeatMasker* (v4.1.2-pl, [18], RRID:SCR_012954) was used to annotate and soft-mask the genome assembly using the species-specific repeats library generated by *RepeatModeler*, and families were labeled accordingly.

#### Ribosomal DNA

Assembled scaffolds were searched for ribosomal DNA (rDNA) units using *ribocop.py*, which searches for consecutive alignments of 18S, 5.8S, and 28S to determine rDNA sequences.

#### De novo gene annotations

RNA-seq data from multiple tissues (Supplementary Table S2) were processed using *Trinity* (v2.12.0, [54], parameters: –min_kmer_cov 3 –trimmomatic) to produce individual transcriptome assemblies. Parameters were chosen to remove low abundance and sequencing error *k*-mers. The assembled transcripts were aligned to the UniProt-SwissProt database (last accessed 28 February 2024) using *diamond* (v2.1.9, [55], parameters: blastx –max-target-seqs 1 –iterate –min-orf 30). Alignments were processed using the *blastxtranslation.pl* script to obtain putative open reading frames and corresponding amino acid sequences. Transcripts containing both the start and the stop codons, with translated sequence length between 95% and 105% of the best hit to the UniProt_SwissProt sequence, were selected as full-length transcripts.

Amino acid sequences of full-length transcripts were processed using *CD-HIT* (v4.8.1, [56], parameters: -c 0.8 -aS 0.9 -g 1 -d 0 -n 3) to cluster similar sequences with 80% pairwise identity and where the shorter sequence of the pair aligned at least 90% of its length to the larger sequence. A representative transcript from each cluster was aligned to the repeat-masked genome using *minimap2* (v2.26, parameters: –splice:hq), and alignments were coordinate-sorted using *samtools*. Transcript alignments were converted to *gff3* format using *AGAT* (v1.4.0, [57], agat_convert_minimap2_bam2gff.pl) and parsed with *genometools* (v1.6.2, [58]) to generate training gene models and hints for *Augustus* (v3.4.0, [59]) with untranslated regions (UTRs). Similarly, transcripts containing both start and stop codons with translated sequence length outside of 95% and 105% of the best hit to the UniProt_SwissProt sequence were processed in the same way to generate additional hints. A total of 500 of these representative full-length transcripts were used in training for gene prediction to calculate species-specific parameters. During the gene prediction model training, parameters were optimized using all 500 training gene models, with a subset of 200 used only for intermediate evaluations to improve runtime efficiency. Gene prediction for the full dataset used 20-Mbp chunks with 2-Mbp overlaps to improve runtime efficiency.

An issue was identified where the predicted *Amh* gene on the Z-specific scaffold (scaffold 18) was fused with neighboring genes. To resolve this, gene prediction was rerun on the Z scaffold with manually modified hints. Specifically, the weighting of UTR hints intersecting with the 2 predicted introns flanking the *Amh* coding sequence was increased to down-weight intronic predictions by *Augustus* in that region. The updated Z scaffold gene predictions were then concatenated with the original gene predictions.

Completeness of annotated genes was evaluated using *BUSCO* (v5.8.2, parameters: –mode protein) in offline mode using the sauropsida_odb10 library. Predicted genes were aligned against the Uniprot_Swissprot database for functional annotation using the best-hit approach and *diamond*. Unaligned genes were subsequently aligned against the Uniprot_TrEMBL database for functional annotation (RRID:SCR_002380).

## Results and Discussion

### DNA sequence data quantity and quality

**Fig. 3:**
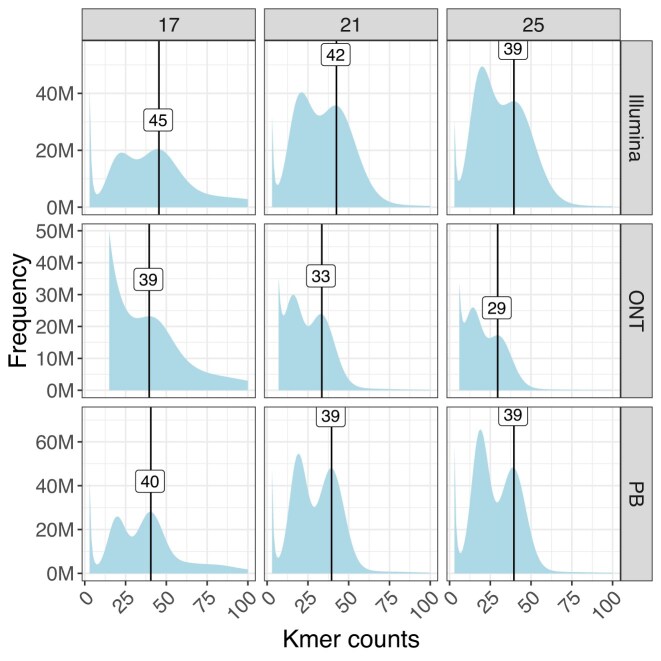
Distribution of *k*-mer counts frequency using sequences from Illumina, Oxford Nanopore Technologies (ONT), and PacBio (PB) platforms for the bearded dragon *P vitticeps*. Heterozygosity is high, as indicated by dual peaks in each graph, and the height of the heterozygous peak increases with the length of the *k*-mer. This confirms diploidy.

PacBio HiFi sequencing yielded 70.6 Gbp with a mean read length of 14,980 bp (Table 1, Supplementary Table S3) and mean quality value >Q30 of all reads. The ONT sequencing yielded 105.6 Gbp of reads with an N50 value of ∼37 Kbp and 48.1% reads with mean quality value >Q20 (Table 1, Supplementary Table S4). High molecular weight DNA often results in lower yields for the ONT platform. The distributions of quality scores and read lengths for the long-read sequencing align with known characteristics of the ONT and PacBio platforms (Supplementary Fig. S1). The *k*-mer frequency histograms of Illumina, ONT, and PacBio HiFi sequence data for *k* = 17, *k* = 21, and *k* = 25 show 2 distinct peaks (Fig. 3), confirming the diploid status of this species. The peak for heterozygous *k*-mers was smaller for *k* = 17 compared to the homozygous *k*-mer peak. In contrast, the heterozygous *k*-mer peak was higher for *k* = 25 compared to the homozygous *k*-mer peak, suggestive of high heterozygosity at a small genomic distance. Genome size was estimated to be 1.81 Gbp using the formulae of Georges et al. [16] and Illumina sequence data, with a *k*-mer length of 17 bp, a homozygous peak of 45.5 (Fig. 3), and the mean read length of 134.3 bp. However, the PacBio estimate of genome size of 1.74 Gbp agrees more closely with the previous estimate using earlier Illumina reads [16] and the estimate from flow cell cytometry of 1.77 Gbp [16]. The reason for the discrepancy between the current and former estimates of genome size from Illumina data is unclear but may have arisen because the current Illumina data were not filtered for error reads in the same way.

**Table 1:** Summary metrics for sequence data and assembly for the bearded dragon *P. vitticeps*

Sequencing platform	Number of reads	Mean read length (bp)	Total bases	Est. genome size (Gbp)	Read depth
Illumina PE DNA	694,401,150	134	93,255,202,850	1.81	52.6*×*
PacBio HiFi Sequel II	4,714,654	14,980	70,625,888,904	1.74	40.4*×*
ONT R9.4.1	7,118,515	14,830	105,566,275,033	2.7	60.5*×*
Arima Genomics HiC	590,697,330	151	89,195,296,830	–	–

Read depth, obtained by dividing the total DNA sequence data from each platform by the assembly size, was consistent (Table 1) with the median read depths of 60.5*×* for ONT, 40.4*×* PacBio HiFi, and 52.6*×* Illumina platforms calculated for 10-Kbp non-overlapping sliding windows of the assembly.

### Assembly

*Hifiasm* produced 3 assemblies: one for each haplotype and a pseudo-haplotype of high quality, as evidenced by assembly metrics (Table 2). The haplotype assemblies were subject to further scaffolding and misjoin error correction using the HiC data to improve assembly contiguity (Supplementary Fig. S2). Minimal manual curation was required as wrongly joined scaffolds were corrected by YaHS (Supplementary Fig. S2). The reference assembly for the central bearded dragon had a total length of 1,752,814,424 bp assembled into 89 scaffolds, with 26 gaps each marked by 100 Ns. This compares well with other published squamate genome assemblies.

**Table 2:** Summary metrics for the genome assembly of the bearded dragon *Pogona vitticeps*. The pseudo-haplotype is a combination of haplotypes 1 and 2 (sensu *hifiasm*); the Reference Assembly was constructed by selecting the best scaffolds from each of haplotypes 1 and 2

Metric	Haplotype 1	Haplotype 2	Pseudo-haplotype	Reference Assembly
Assembly length (bp)	1,752,200,003	1,747,167,247	1,747,460,405	1,752,814,424
No. of scaffolds/contigs	89	51	71	89
GC content (%)	42.2	42.2	42.2	42.2
No. of gaps >100 bp	31	28	15	26
Mean sequence length (bp)	19,687,640	34,258,181	24,612,118	19,694,544
Median sequence length (bp)	77,000	152,108	85,021	77,000
Longest sequence (bp)	359,918,989	358,276,425	359,349,958	358,276,425
Shortest sequence (bp)	4,000	18,330	4,000	4,000
N50 (bp)	265,980,915	266,210,064	266,029,613	266,210,064
N90 (bp)	28,115,431	28,121,876	28,118,385	28,115,431
L50	3	3	3	3
L90	9	9	9	9

The central bearded dragon reference genome (PviZW2.1) is contiguous with a scaffold N50 value of 266.2 Mbp and an N90 value of 28.1 Mbp, with the largest scaffold of 358.3 Mbp (Table 2). L50 and L90 values were 3 and 9, respectively, typical of species with microchromosomes, where most of the genome is present in large macrochromosomes. The highly contiguous scaffolded assembly was obtained because the foundational contigs had an N50 value of 134.1 Mbp and an N90 value of 14.1 Mbp and a largest contig at 240.1 Mbp.

**Figure 4:**
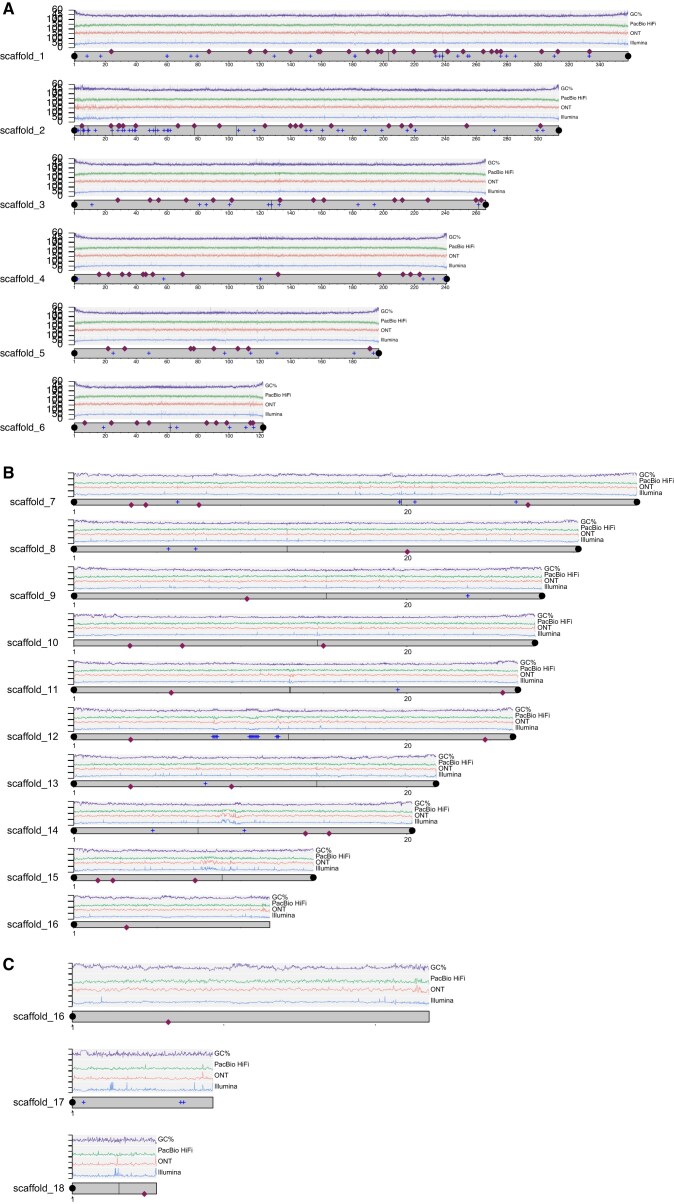
A plot of the 18 longest scaffolds (corresponding to the number of chromosomes of the bearded dragon *P. vitticeps*. Four traces are shown on each chromosome. The top trace (purple, range 30%–60%) represents GC content, the next trace (green, range 0–50*×*) represents PacBio HiFi read depth, the next trace (red, range 0–100*×*) represents ONT read depth, and the fourth trace (blue, range 0–100*×*) represents Illumina read depth. Note that there is no indication in any of these traces of centromeric position in contrast to *Bassiana* [60]. Telomeres are shown as black dots; satellite repeats are indicated by the blue plus symbols (+) and gaps by vertical black lines. The red diamonds show the location of BAC anchors ([13, 14], Supplementary Table S7). Locations of the putative centromeres are shown in Fig. 5. (A) Macrochromosomes. (B) Microchromosomes. (C) Both the Z- and W-specific regions were assembled into single scaffolds, with the PAR assembled into a single scaffold in both haplotype**s**. Refer to Supplementary materials for a high-resolution version of this figure.

**Figure 5:**
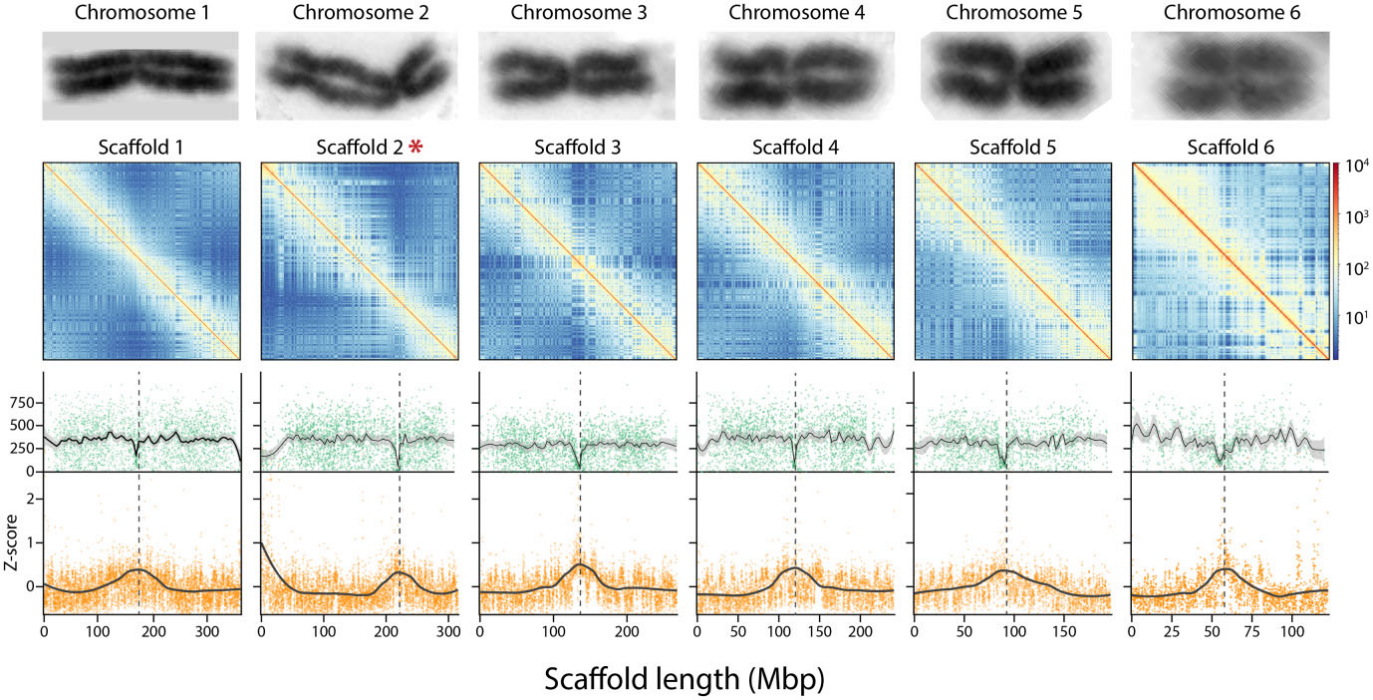
Identification of putative centromeres for the 6 macrochromosomes. The upper row of panels gives chromosome-specific HiC heatmaps showing intrachromosomal interactions. The second row of panels shows the count of heterozygous sites per 50-Kbp window (green dots) with lines of best fit and 95% confidence interval (gray shading). The lower row of panels shows the *z*-scores for interchromosomal HiC interactions along chromosome length (Mbp) with smoothed lines of best fit. Each dot in the lower panels represents the *z*-score interaction value of a different 50-Kbp bin. Chromosomes images are taken from Ezaz et al. [11] and are not to scale. They are to illustrate the correspondence between the karyotype centromere and the putative position of the centromere (dashed lines) inferred from the dip in heterozygosity and the peak in interchromosomal contact. Scaffold 2 marked (*) is inverted with respect to the published karyotype. Refer to Supplementary Fig. S3 for similar plots for the microchromosomes.

All 15 major scaffolds in the assembly (corresponding to autosome number in the karyotype of the bearded dragon) had well-defined telomeres at each end (Fig. 4). Telomeres comprised the vertebrate telomeric motif TTAGGG and ranged in size from 2,430 bp (405 copies of the repeat motif) to 42,098 bp (7,151 repeat copies). The telomeric regions were typically characterized by an expected rise in GC content (Fig. 4) and a significant rise in interchromosomal contact (Fig. 5; Supplementary Fig. S3), mirroring patterns previously described in turtles [41].

**Figure 6:**
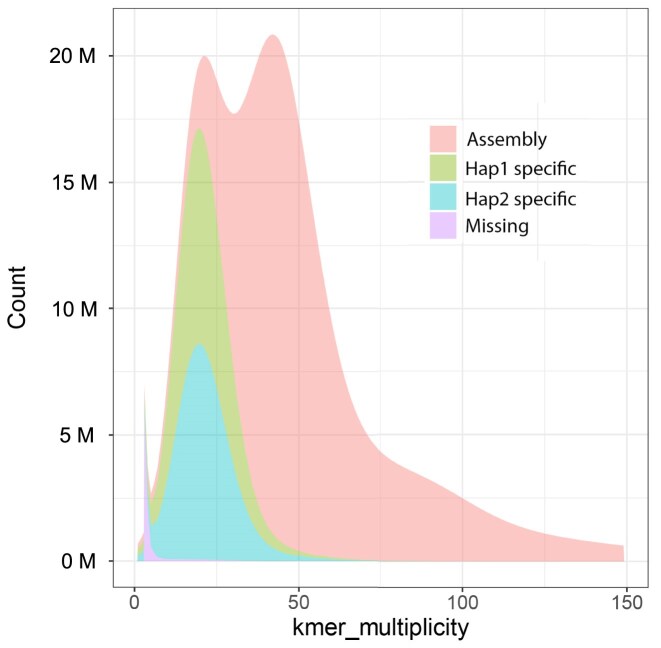
Distribution of Illumina *k*-mers (k = 17) in the genome assembly of the bearded dragon *P. vitticeps* (Supplementary Table S6). The *k*-mer counts are shown on the x-axis and the frequency of occurrence of those counts on the y-axis. Those scored as missing are found in reads only.

Initially, we did not detect a telomere repeat sequence on the 5′ end of scaffold 10 using a stringent threshold of 600 bp for the telomeric region. However, manual examination revealed 32 repeats of the telomeric sequence from position 1–214 on scaffold 10, verifying that it had telomeres at both ends. The missing telomere for scaffold 16 is expected because it is the pseudo-autosomal region of the sex chromosomes. The putative sex chromosome scaffolds 17 = W and 18 = Z also each possessed only 1 terminal telomeric sequence. This is consistent with T2T assembly for the sex chromosomes once the PAR and the non-recombining regions of Z and W are combined.

Typical centromeric satellite repeats units were not evident in the repeat structure, read depth profiles, or GC content profiles (Fig. 4) as they were for *Bassiana duperreyi* [60]. Putative centromeric regions were evident for the macrochromosomes as an increase in the levels of interchromosomal contact in the HiC data and as a drop in heterozygosity (Fig. 5 and Supplementary Fig. S3).

Of 137 BAC clones [13, 14], 5 with single sequences did not align, 2 had interchromosomal mappings, 14 had discrepant mappings for macrochromosomes, and 2 had end sequences that were too far apart to be considered valid. This left 114 clones (83.2%) with reliable mappings. This physical mapping validated the assignment of assembly scaffolds 1–6 to the macrochromosomes 1–6 of the genome (Fig. 4A), along with the assignment of scaffolds 7–15 to the microchromosomes (Fig. 4B), albeit with altered order (Fig. 8); scaffold 16 to the PAR of the sex chromosomes (Fig. 4C); and scaffold 18 as the nonrecombining region of the Z chromosome (Fig. 4C). Mapping of the W-linked sequence clone C1 (3,288 bp, [29]) confirmed the identity of scaffold 17 as the non-recombining region of the W chromosome (Fig. 4C).

### Assembly evaluation

The percent collapsed sequence in the assembly was exceptionally low at 0.003% (492,971 bp, 54–13,643 bp, *n* = 255), as was the percentage of false expansions at 0.03% (49,447 bp, 52–5,133 bp, *n* = 69)—2 of the indicators of genome assembly quality identified by the Earth Biogenome Project [17].

Completeness of the assembly was estimated to be 99.82% for both haplotype assemblies combined, and the per base assembly quality estimate exceeded Q40 at 48.36 (1 error in 68.5 Kbp). High heterozygosity in the *k*-mer profiles (Fig. 3) affected assembly completeness metrics measured by *Merqury*. Individual haplotype assemblies were 85.5% complete, which is expected of animals with a high heterozygosity (in our case, 1.98%). This shows that assembly completeness metrics for a single haplotype assembly measured using *k*-mers can be understated for species with a high heterozygosity (Fig. 6).

**Figure 7:**
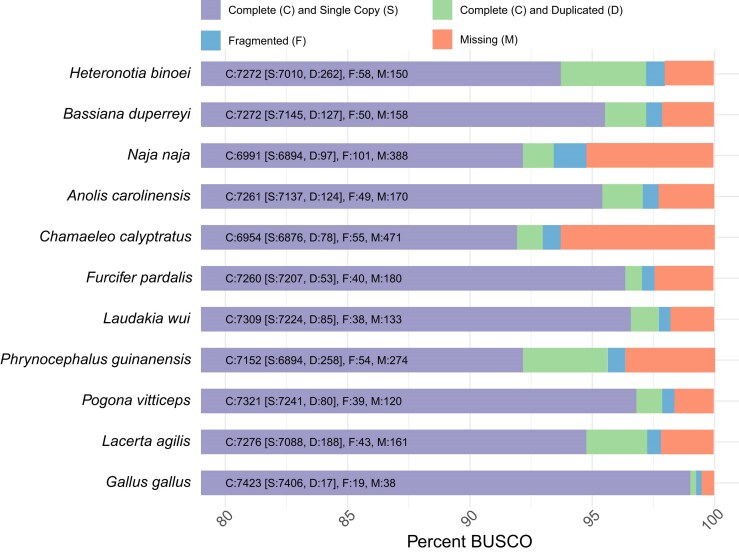
A visual representation of how complete the gene content is for each listed species genome, including *P. vitticeps*, based on BUSCO (*n* = 7,480).

Analyses using the BUSCO gene set for Sauropsids reveals 7,321 genes as complete (97.9%), with a minimal proportion duplicated (D: 1.1%), indicating a robust genomic structure with minimal redundancy (Fig. 7). The central bearded dragon genome also had a low proportion of fragmented (F: 0.5%) and missing (M: 1.6%) orthologs. These results positioned the central bearded dragon favorably in terms of genome completeness and integrity, on par with other squamates, highlighting its potential as a reference for further genomic and evolutionary studies within this phylogenetic group. In our comparison set, only chicken (*Gallus gallus*) has better BUSCO statistics than the bearded dragon. RNA-seq data mappability was on average 93.5%, and 18 of 22 samples had more than 90% of fragments mapped to the genome (Supplementary Table S2). Note that sensitivity settings for alignments had to be increased for mapping RNA-seq data given high hetergozygosity observed for this species (1.98%).

## Chromosome Assembly

**Figure 8:**
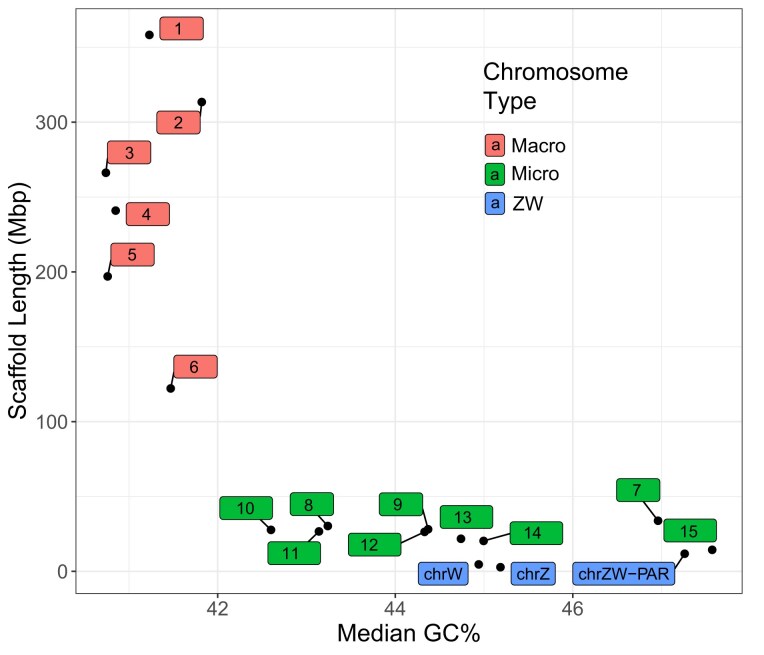
A plot of assembly scaffolds defined by scaffold length versus median GC content in 10-Kbp windows. Microchromosomes are characterized by a higher GC content than macrochromosomes. Median GC content in 10-Kbp windows of scaffolds versus length of scaffolds representing macrochromosomes (scaffolds 1–6, red), the sex chromosomes (blue, the PAR and nonrecombining regions of the Z and W), and the other microchomosomes (green, scaffolds 7–15). Scaffold numbers 1–6 correspond to the macrochromosome numbers of Deakin et al. [13] for scaffolds. Scaffold numbers 7–15 translate to the microchromosome numbers of Deakin et al. as per Supplementary Table S6.

The bearded dragon has 2*n* = 32 chromosomes with 6 pairs of macrochromosomes and 10 pairs of microchromosomes, including the sex chromosomes. The distinction between macro- and microchromosomes typically relies on a bimodal distribution of size, but other characteristics, such as GC content, provide additional evidence for this classification [41, 61] (Fig. 8). The median GC content of 10-Kbp windows for the 6 largest scaffolds (representing macrochromosomes) ranged between 40.7% and 41.8%. In contrast, the remaining 12 scaffolds ordered by decreasing length had a median GC content of between 42.6% and 47.6%, characteristic of microchromosomes in other squamates.

**Figure 9:**
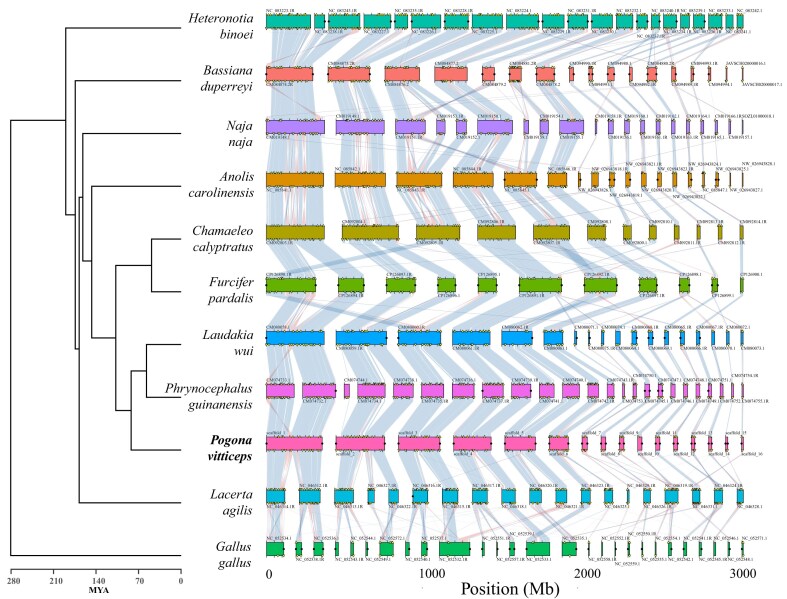
Synteny conservation of BUSCO homologs for the bearded dragon *P. vitticeps* and squamates with chromosome-level assemblies, including representative skink, iguanid, snake, and gecko lineages and chicken. Synteny blocks corresponding to each species are aligned horizontally, highlighting conserved chromosomal segments across the genomes. The syntenic blocks are connected by ribbons that represent homologous regions shared between species, with the varying colors denoting segments of inverted gene order. Duplicated BUSCO genes are marked with yellow triangles. Predicted telomeres are marked with black circles.

Unlike mammals, reptiles (including most birds) show a high level of chromosomal homology across species [41, 61]. Figure 9 shows synteny conservation between bearded dragon, representative squamate species, and chicken. Apart from a handful of intrachromosomal rearrangements, the major scaffolds of bearded dragon and other squamates corresponded well, including the PAR of the sex microchromosomes (scaffold 16) within the Agamidae. When compared with other genomes in the analysis, the bearded dragon genome showed a high degree of evolutionary conservation with respect to both chromosomal arrangement and gene order (Fig. 9).

**Figure 10:**
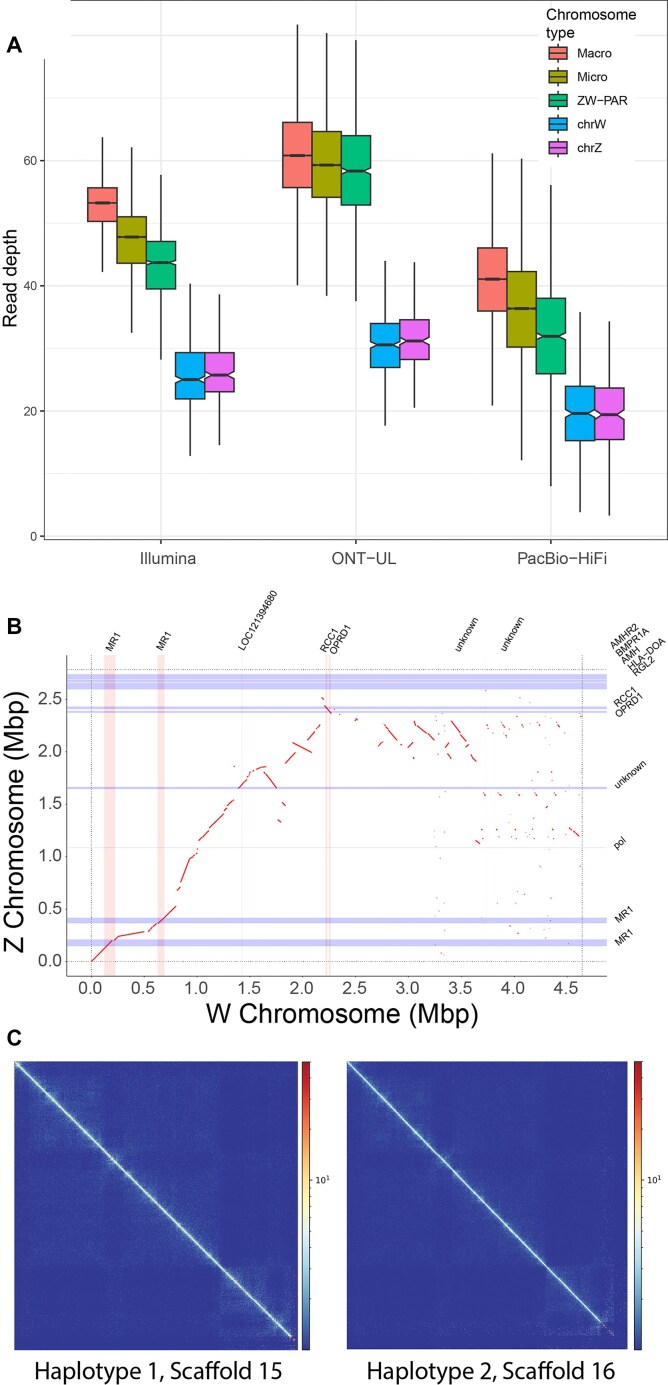
Sex chromosome analysis. (A) For each sequencing technology, boxplots of read depth in 10-Kbp windows of macrochromosomes, microchromosomes, the PAR, and Z- and W-specific scaffolds. Boxes represent the middle 50% of the data, notches represent 95% confidence intervals of the medians (central horizontal black bar), whiskers are ±1.5 the interquartile range, and outliers are not plotted. (B) Alignment of the Z-specific (y-axis) and W-specific (x-axis) scaffolds. Red lines represent homologies. Blue horizontal bars are genes annotated on the Z scaffold (gene names given on the y-axis), and pink vertical bars are genes annotated on the W scaffold (gene names given on the x-axis). (C) Phase HiC contact maps of the PAR in the 2 different haplotypes. Note that it is unknown which is the Z PAR and which is the W PAR.

The Z- and W-specific sex chromosome scaffolds were identified as 18 and 17, respectively. These represent the non-recombining region of the sex chromosomes. They were not assembled to the PAR in either haplotype despite the use of HiC data for scaffolding. The Z-specific scaffold was 2.78 Mbp, and the W-specific scaffold was 4.64 Mbp. In the sequenced ZW female, read depth for both scaffolds was identified based on the median read depth in 10-Kbp sliding windows. As expected, read depth was approximately half that of the autosomes and the PAR scaffold (Fig. 10A). The first half of the Z and W scaffolds shared good homology (Fig. 10B). On the second half of the W scaffold, there appeared to have been duplication and expansion that increased its size relative to the Z. The PAR scaffold (scaffold 16 reference, 11.77 Mbp) was identified by homology to known Z sequences from Pvi1.0.

The W-specific scaffold had 7 annotated genes, with none presenting as an obvious sex-determining candidate. The Z-specific scaffold also had 7 annotated genes, 4 of which were ZW shared (Fig. 10B). Notably, copies of both *Amh* and its receptor (*AmhR2*) were located on the Z, presumably duplicated from the autosomal homologues, which remained present on scaffolds 7 and 2, respectively. Both genes are central to the sex-determining pathway in other vertebrates, so they present as strong sex-determining candidates that would presumably function in a dosage-dependent manner.

HiC reads were phased to the PAR scaffolds to determine if there was a different 3-dimensional structure of the Z and W scaffolds (see [15]). Surprisingly, despite clear cytological differences between the Z and W in cultured fibroblasts [11], there was little difference between HiC contact maps for each haplotype (Fig. 10C). The discrepancy between cytogenetic (fibroblasts) and HiC data (blood) with respect to the Z and W structure likely arises from the different cell types examined. Alternatively, the cytogenetic data only capture cells in metaphase when 3-dimensional genome structure differences might be at their most pronounced.

### Annotation

#### General repeat annotation

An estimated 45.6% (798 Mbp) of the bearded dragon genome was composed of repetitive sequences, including interspersed repeats, small RNAs, and simple and low-complexity tandem repeats (Table 3). Retroelements (short interspersed nuclear elements [SINEs] and long interspersed nuclear elements [LINEs]) were the most common repetitive element (17.3%). DNA transposons were the second most common repetitive element (5.1%) and were dominated by Tc-Mar and hAT elements (Supplementary Table S8). CR1, BovB, and L2 elements were the dominant long interspersed elements (81% of LINE elements; 13.4% of the genome), which is consistent with other squamate genomes [62]. A total of 37% of all repeat content was unclassified and did not correspond to any element in the RepeatModeler libraries. Refer to Supplementary Fig. S4 for the size distribution of these unclassified repeats. The number of elements masked and their relative abundances are presented in the supplementary material (Supplementary Table S8).

**Table 3:** Condensed summary of the copy number and percentage of the bearded dragon (*P. vitticeps*) genome covered by repeat elements. Refer to Supplementary Table S8 for a full breakdown

Family	Numbers of elements	Length masked (bp)	% of sequence
Retroelements	1,061,885	303,713,020	17.33
SINEs	114,787	15,769,671	0.90
LINEs	947,098	287,943,349	16.43
Long terminal repeat elements	80,454	72,706,738	4.15
DNA transposons	432,771	89,795,030	5.12
Penelope-like elements	1,797	150,905	0.01
Rolling circles	447	95,044	0.01
Unclassified retroelements	1,775,114	294,309,581	16.79
**Total interspersed repeats**	**3,352,468**	**760,770,318**	**43.40**
Satellite	4,246	1,284,141	0.07
Simple repeat	576,326	34,276,116	1.96
Ribosomal RNA	402	1,812,567	0.10
Small nuclear RNA	2,397	502,447	0.03
Transfer RNA	101	6,775	0.00
**Total masked**		**798,652,364**	**45.56**

### Satellite repeats

We undertook a more detailed analysis in an attempt to identify centromeric satellite repeats and centromeric regions as we did for the genome assembly of the skink *B. duperreyi* [60]. The 67 satellite repeat units identified in the KMC/SRF analysis had lengths between 5 and 9,460 bp. These collapsed into 45 distinct classes based on sequence similarity (Supplementary Table S9).

One repeat class (srfclass-16) corresponded to the telomeric microsatellite repeats (TTAGGG). A second class (srfclass-18) with a large repeat unit of 9,460 bp corresponded to a ribosomal DNA sequence, dealt with in more detail later. A class of interspersed repeats (srfclass-11), possibly LINE elements, comprised 5,695 bp in unit length. A class of repeats (srfclass-30) was present as 84 copies on scaffold 4 (119,114,420–119,423,712); all other copies were interspersed across the genome. A telomeric repeat was embedded in this larger repeat. A fifth class (srfclass-21) comprised repeat units of 2,190 bp on scaffold 1 (117 copies, 167,719,827–167,977,892) and was somewhat enigmatic. These units were tandemly organized as 1 to 43 repeats, occasionally with a small intervening sequence. This repetitive sequence was found also on other scaffolds as interspersed units comprising a 1,406-bp motif and a 606-bp motif separated by a 500-bp intervening sequence. A sixth class (srfclass-38) comprised repeat units of 877 bp, found only on scaffold 1 (238 copies, 54,463,716–254,497,379). A seventh class (srfclass-15) comprised repeat units of 398 bp, each as a composite of a 68-bp subunit, on scaffold 1 (181,407,117– 181,830,275, ca. 1,063 copies). These repeat units align with elements on scaffolds 3, 4 and 5, but with abbreviated subunits (e.g., 56 bp on scaffold 3; 64 bp on scaffold 4). An eighth repeat class (srfclass-4) comprised a 98-bp motif that occurred on the W chromosome scaffold 17 (1,961 copies, 281,785–473,916), found as 109- to 450-bp alignments on other scaffolds. Of the 45 repeat classes, only 1 (srfclass-5,151 bp) showed potential as a centromeric repeat unit. However, this repeat class was not distributed as a single consolidated cluster on each chromosome, as would be expected of centromeric repeat units.

We were thus unable to definitively identify centromeric repeat units in the bearded dragon to confirm the presence of only 1 per chromosomal scaffold as we were able to do in the skink *B. duperreyi* [60]. We were, however, able to confirm the likely presence of 1 centromere per scaffold as expected if the scaffolds correspond to chromosomes using plots of heterozygosity and an index of interchromosomal contact rates against position on the scaffold (Fig. 5). A dip in heterozygosity corresponded to a peak in HiC interchromosomal contact rate, which together corresponded well with the position of the centromere taken from metaphase chromosomal spreads [11].

## Gene Annotation

We assembled transcriptomes from 22 samples (Supplementary Table S2). Genome annotation using *Augustus* predicted 17,237 genes and transcripts, of which 16,799 had a match to a Uniprot_Swissprot or Uniprot_TrEMBL protein sequence, and 16,483 were assigned a gene name. Analyses using the BUSCO gene set (v5.8.2, *n =* 7,480 genes) for Sauropsids reveals 6,558 genes as complete (87.7%), with 1.3% duplicates. There were 237 genes (3.2%) identified as fragmented and a further 685 genes (9.2%) as missing in the annotated protein sequences. These data suggest relatively accurate gene prediction. The quality of the annotation was further validated using RNA-seq data from 22 samples, with an average 54.4% (ranging from 22.8% to 76.4%) of aligned reads assigned to annotated exons, indicating a reasonable level of correspondence between the predicted gene models and the observed transcriptomes. Relatively lower mapping rates of RNA-seq data can be attributed to incomplete annotations of untranslated regions and the presence of other noncoding RNAs that are not annotated. Further improvements to annotations in the future will likely improve the utility of this genomic resource.

### Mitochondrial genome

The bearded dragon mitochondrial genome assembly was 16,731 bp in size with 37 intact genes without frameshift mutations. It consisted of 22 transfer RNAs, 13 protein-coding genes, 2 ribosomal RNA genes, and the control region (Supplementary Fig. S5), so it was typical of the vertebrate mitochondrial genome. Base composition was A = 33.0%, C = 29.8%, G = 13.1%, and T = 24.0%. Illumina data showed only 12 homopolymer variant sites, suggesting accurate recovery of the mitochondrial sequence.

We note that mitochondrial sequence was absent in the HiFi data presumably because it was eliminated during the size selection step. As the assembly software uses PacBio HiFi for the core assembly, these mitochondrial sequences, although present in the ONT data, were not recovered during the combined assembly process. We also note a drop in the read depth for the PacBio HiFi and Illumina data for exceptionally small microchromosomes (Fig. 10A) that is not observed for ONT data. This suggests a systematic bias in the data from sequence-by-synthesis platforms for small elements and high GC content sequences.

### Ribosomal DNA

The rDNA unit length in the bearded dragon is approximately 9.5 Kbp, with a total of 1.75 Mbp of sequence across 24 scaffolds containing rDNA sequences. The rDNA sequence was found on the chromosome 2 scaffold, as expected [14], near the subtelomeric region of 2q. There were 23 additional short scaffolds composed entirely of rDNA arrays as well, indicating poor-quality assembly of the rDNA array. The first and second internal transcribed spacers (533 bp and 344 bp, respectively) and intergenic spacer (2.7 Kbp) are relatively small compared to mammals [63].

## Conclusion

Here we present a high-quality genome assembly of the central bearded dragon *P. vitticeps*. The quality of the genome assembly and annotation compares well with other chromosome-length assemblies and is among the best for any species of Agamidae. We have chromosome-length scaffolds and near telomere-to-telomere quality.

The non-recombining regions of the Z and W chromosomes were each assembled as a single scaffold. The PAR was assembled as a single scaffold in both haplotypes. The sex chromosomes scaffolds and PAR scaffold each lacked 1 telomere, but this is likely resolved when they are combined to form Z and W scaffolds, including both the PAR and non-recombining regions. The identification of *Amh* and *Amhr2* on the Z-specific scaffold (but not the W) has them as strong candidates for the sex-determining gene(s) in this species. This will be a fruitful area for future functional studies based on upregulation and knockout technologies (e.g., CRISPR knockouts). Such approaches were necessary to establish the *Sry* gene on the Y chromosome of eutherian mammals as the master sex-determining gene acting through dominance [64, 65], as well as establish *Drmt1* on the Z chromosomes of birds as the master sex-determining gene acting though dosage [66, 67]. Gene *Nr5a1*, encoding transcription factor SF1, was previously identified as a candidate sex-determining gene because it resided on the sex chromosomes and because of its differential transcript isoform composition [15]; it is confirmed as residing within the PAR on both the Z and W chromosomes. The concurrent discovery of *Amh* and *Amhr2* as duplicate copies of their autosomal orthologs (see [68]) on the Z chromosome, as well as confirmation here that they do not reside on the W, hints at a dosage-based mechanism of sex determination involving 1 or both of these genes. *Amh* and its receptor *AmhR2* are central to male differentiation in vertebrates and so are predisposed to recruitment as master sex-determining genes on the sex chromosomes. This has occurred multiple times in fish with the enlistment of *Amh* or *AmhR2* to the Y chromosome [69–72] or the involvement of *Amh* in the establishment of a *de novo* sex chromosome [73]. In the frog *Rana temporaria*, the Y chromosome underwent a reciprocal translocation with an autosome, fusing them into a single inherited neo-Y chromosome that included key sex genes *Dmrt1, Amh*, and *AmhR2* [74]. *Amh* is also implicated as the master sex- determining gene in monotremes [75]. Our results indicate that sex determination in the dragon likely involves more complex gene interactions, involving expression of the Z and autosomal copies of *Amh* and *AmhR2*, as well as *Nr5a1*, which encodes transcription factor SF1 and has a foundational involvement in sex determination in vertebrates. The gene *Nr5a1*, although on the PAR, as confirmed here, and with virtually identical copies on the Z and W chromosomes, yields substantially different Z and W transcriptional isoform composition [15]. This suggests that complex interactions between these genes and their resultant transcription factors and intermediaries determine sex in the bearded dragon. This will be a fruitful area for future investigation.

This annotated assembly for the central bearded dragon was generated as part of the AusARG initiative of Bioplatforms Australia, to contribute to the suite of high-quality genomes available for Australian reptiles and amphibians as a national resource. The central bearded dragon is already widely used in research requiring genomic foundations, in large part because of the earlier publication of an assembly based on short-read technologies [16]. The central bearded dragon is an emerging model species [6] because of its high fecundity and short incubation, ease with which it adapts to captivity, and a published genome, all considered key advantages accelerating its use [76]. We anticipate that this new and vastly improved reference genome will serve to accelerate comparative genomics, developmental studies, and evolutionary research on this and other species. As an exemplar of a well-studied oviparous taxon with sex reversal by temperature, the central bearded dragon reference assembly will provide a solid basis for genomic studies of the evolution of the genetic basis for reprogramming of sexual development under the influence of environmental temperature [8–10].

## Editors’ Note

Another near-T2T bearded dragon genome assembly is published alongside this work, sequenced using different sequencing technology and approaches [68].

## Additional Files

**Supplementary Fig. S1**. Comparison of average read quality values (QVs) versus read length for the 2 sequencing technologies: Oxford Nanopore Technologies (ONT) and PacBio HiFi.

**Supplementary Fig. S2**. HiC contact maps for Haplotype 2 showing an assembly misjoin in the YAHS assembly. (a) The original contact map showing the misjoin. (b) The resolved assembly with the misjoin resolved manually.

**Supplementary Fig. S3**. Figure showing identification of putative centromeres for the 6 macrochromosomes and 10 microchromosomes of the bearded dragon *Pogona vitticeps*.

**Supplementary Fig. S4**. Size distribution of the repetitive elements that could not be identified.

**Supplementary Fig. S5**. Annotation of the mitochondrial genome of *Pogona vitticeps* assembled using *flye* and annotated using *mitoHiFI*.

**Supplementary Table S1**. A list of software used for the analyses reported in this article, including version numbers and where it can be accessed.

**Supplementary Table S2**. Summary statistics for the raw Illumina RNA sequence data used to assemble the transcriptome and for annotation.

**Supplementary Table S3**. Summary statistics for the raw PacBio HiFi sequence data used for the assembly.

**Supplementary Table S4**. Summary statistics for the raw Oxford Nanopore sequence data used for the assembly.

**Supplementary Table S5**. Summary statistics for the HiC sequence data used to scaffold the assembly.

**Supplementary Table S6**. Summary statistics for the raw Illumina DNA sequence data.

**Supplementary Table S7**. Bacterial artificial chromosome (BAC) sequences mapped to the assembly scaffolds for the bearded dragon *Pogona vitticeps*.

**Supplementary Table S8**. Satellite repeat units of the genome assembly for the bearded dragon *Pogona vitticeps* collapsed into 45 distinct classes based on sequence similarity.

**Supplementary Table S9**. Summary of the copy number and percentage of the bearded dragon (*Pogona vitticeps*) genome covered by repeat elements

giaf085_Pogona_genome_Supplementary-Revision

giaf085_Authors_Response_To_Reviewer_Comments_Original_Submission

giaf085_GIGA-D-25-00166_Original_Submission

giaf085_GIGA-D-25-00166_Revision_1

giaf085_Reviewer_1_Report_Original_Submission Yuan Li -- 5/15/2025

giaf085_Reviewer_2_Report_Original_Submission Heiner Kuhl -- 5/31/2025

## Abbreviations

BAC: bacterial artificial chromosome; BUSCO: Benchmarking Universal Single-Copy Orthologs; EBP: Earth BioGenome Project; HiC: high-throughput chromosome conformation capture; HiFi: high fidelity; L50: minimum number of contigs (or scaffolds) to add in length to 50% of assembly length; L90: minimum number of contigs (or scaffolds) to add in length to 90% of assembly length; LINE: long interspersed nuclear element; LTR: long terminal repeat; N50: median (50th percentile) contig or scaffold length; N90: 90th percentile of contig or scaffold length; NCBI: National Center for Biotechnology Information; ONT: Oxford Nanopore Technologies; PacBio: Pacific Biosciences; PAR: pseudo-autosomal region; Q20: Phred score of 20 corresponding to a 1% error rate; Q30: Phred score of 30 corresponding to a 0.01% error rate; rDNA: ribosomal DNA; RNA-seq: RNA sequencing; SINE: short interspersed nuclear element; T2T: telomere-to-telomere; UTR: untranslated region.

## Data Availability

The supplementary file contains a description of all supplemental materials, which include tables showing software used in the preparation of this article, outcomes of the sequencing on the 4 sequencing platforms used, and figures in support of statements on the quality of data. The authors affirm that all other data necessary for confirming the conclusions of the article are present within the article, figures, and tables. The annotated assembly can be accessed from NCBI as PviZW2.1, and all reads used in support of the assembly are lodged with the Short Read Archive. All sequence data generated in this study are available from NCBI SRA under BioProject ID PRJNA1252275. Accession numbers are provided in the main text and the supplementary tables (Supplementary Tables S2–S6). High-resolution versions of figures and custom scripts used to conduct the analyses are at https://github.com/kango2/ausarg/. Additional data are available in the *GigaScience* repository, GigaDB [77].
