# Supplementary material for: A near telomere-to-telomere phased genome assembly and annotation for the Australian central bearded dragon Pogona vitticeps
Source: Gigascience. 2025 Aug 19;14:giaf085. doi: 10.1093/gigascience/giaf085 (PMC12360841; doi:10.1093/gigascience/giaf085)
Supplement: giaf085_Pogona_genome_Supplementary-Revision [file giaf085_pogona_genome_supplementary-revision.docx]

Supplementary Materials

**A telomere to telomere phased genome assembly and annotation for the Australian central bearded dragon *Pogona vitticeps***

Hardip R. Patel, Kirat Alreja, Andre L.M. Reis, J King Chang, Zahra A. Chew, Hyungtaek Jung, Jillian M. Hammond, Ira W. Deveson, Aurora Ruiz-Herrera, Laia Marin-Gual, Clare E. Holleley, Xiuwen Zhang, Nicholas C. Lister, Sarah Whiteley, Lei Xiong, Duminda S.B. Dissanayake, Paul D. Waters, Arthur Georges

## List of Tables

**Table S1.** A list of software used for the analyses reported in this paper including version numbers and where it can be accessed.

**Table S2.** Summary statistics for the raw Illumina RNA sequence data used to assemble the transcriptome and for annotation.

#### **Table S3.** Summary statistics for the raw PacBio HiFi sequence data used for the assembly.

#### **Table S4.** Summary statistics for the raw Oxford Nanopore sequence data used for the assembly.

#### **Table S5.** Summary statistics for the HiC sequence data used to scaffold the assembly.

#### **Table S6.** Summary statistics for the raw Illumina DNA sequence data.

#### **Table S7.** Bacterial Artificial Chromosome (BAC) sequences mapped to the assembly scaffolds for the bearded dragon *Pogona vitticeps*.

**Table S8.** Satellite repeat units of the genome assembly for the bearded dragon *Pogona vitticeps* collapsed into 45 distinct classes based on sequence similarity.

**Table S9.** Summary of the copy number and percentage of the bearded dragon (*Pogona vitticeps*) genome covered by repeat elements

## List of Figures

**Figure S1.** Comparison of average read quality values (QV) versus read length for the two sequencing technologies: Oxford Nanopore Technologies (ONT) and PacBio HiFi.

**Figure S2.** HiC contact maps for Haplotype 2 showing an assembly mis-join in the YAHS assembly. (a) The original contact map showing the mis-join; (b) the resolved assembly with the mis-join was resolved manually.

**Figure S3.** Figure showing identification of putative centromeres for the six macrochromosomes and 10 microchromosomes of the bearded dragon Pogona vitticeps.

**Figure S4.** Size distribution of the repetitive elements that could not be identified.

**Figure S5**. Annotation of the mitochondrial genome of *Pogona vitticeps* assembled using *flye* and annotated using *mitoHiFi*.

## Custom Scripts

Custom scripts used to conduct the analyses are at https://github.com/kango2/ausarg/.

**Script 1**: *pacbiobam2fastx.sh* A custom script to remove any reads containing PacBio adapter sequences and convert the .bam files to FASTQ.

**Script 2**: *calculateGC.py* A custom script to calculate GC content in non-overlapping sliding windows of 10 Kbp.

**Script 3.** *processtrftelo.py* A script to identify regions >600 bp that contained conserved vertebrate telomeric repeat motif (TTAGGG).

**Script 4.** *ribocop.py* A script to search for consecutive alignments of 18S, 5.8S, and 28S to determine rDNA sequences.

## High Resolution Figures

**Figures 4 (a-c)**. A plot of the 16 longest scaffolds (corresponding to the number of chromosomes of the bearded dragon *Pogona vitticeps*.

**Figures 10 (a-c).** Sex chromosome analysis. a) For each sequencing technology, boxplots of read depth in 10 Kbp windows of macrochromosomes, microchromosomes, the PAR, and Z and W specific scaffolds.

**Table S1.** A list of software used for the analyses reported in this paper. Included are the use to which the software was put, the version number used, the source of the latest release of the software, and the associated published reference if available.

| Software | Use case | Version | URL (latest release) | Reference |
| --- | --- | --- | --- | --- |
| *Arima Genomics Alignment Pipeline* | Align HiC data to the interim haplotype consensus | 03 | https://github.com/ArimaGenomics/mapping_pipeline | Wingett et al. (2015) |
| *3D-DNA* | Visualisation of HiC scaffolding | 180922 | https://github.com/aidenlab/3d-dna | Dudchenko et al. (2023) |
| *AGAT* | Conversion from bam to gff3 | 1.4.0 | https://github.com/NBISweden/AGAT | Dainat, 2022 |
| *Augustus* | De novo gene annotations | 3.4.0 | https://github.com/Gaius-Augustus/Augustus | Stanke et al. (2008) |
| *bcftools* | Normalise vcf files | 1.14 | https://samtools.github.io/bcftools/ | Danecek et al. (2021) |
| *Biopython* | Python tools for Computational Molecular Biology | 1.79 | https://github.com/biopython/biopython | Cock et al. (2009) |
| *BLAST* | Vector contamination | 2.12.0 | https://blast.ncbi.nlm.nih.gov/Blast.cgi | Camacho et al. (2009) |
| *BUSCO* | Standard gene set | 5.4.7 | https://busco.ezlab.org/ | Manni *et al.* 2021 |
| *buttery-eel* | ONT basecalling wrapper for Dorado |  | <https://github.com/Psy-Fer/buttery-eel> | <https://doi.org/10.1093/bioinformatics/btad352> |
| *bwa-mem* | Short-read alignments | 0.7.17 | https://sourceforge.net/projects/bio-bwa/files/ | Li and Durban, (2010) |
| *CD-HIT* | Clustering of redundant transcript sequences across multiple samples | 4.8.1 | https://sites.google.com/view/cd-hit | Fu et al. (2012) |
| *chromsyn* | Synteny plotting tool |  | https://github.com/slimsuite/chromsyn | Edwards et al. (2022) |
| *cutadapt* | Trim reads containing adapter sequences | 3.7 | <https://github.com/marcelm/cutadapt> | <https://doi.org/10.14806/ej.17.1.200> |
| *dorado* | ONT basecalling | 7.2.13 | <https://github.com/nanoporetech/dorado> | (c) 2024 Oxford Nanopore Technologies PLC |
| *diamond* | Aligning transcriptomic and peptide sequences to uniprot databases for annotation | 2.1.9 | https://github.com/bbuchfink/diamond | Buchfink et al. (2021) |
| *flye* | Mitochondrial genome assembly | 2.9.5 | https://github.com/mikolmogorov/Flye | Kolmogorov et al., 2019 |
| *freebayes* | Creating vcf files | 1.3.8 | https://github.com/freebayes/freebayes | Garrison & Marth (2012) |
| *GEMmapper* | Mapping HiC data to the reference genome | 3.6.1 | https://github.com/smarco/gem3-mapper | Marco-Sola et al. (2012) |
| *genometools* | Parse gff3 annotation files | 1.6.2 | https://github.com/genometools/genometools | Gremme et al. (2013) |
| *GNU parallel* | Parallel processing of commands | 20191022 | https://www.gnu.org/software/parallel/ | Tange, 2018 |
| *HiCexplorer* | Visualising HiC contact maps | 3.7.3 | https://hicexplorer.readthedocs.io/en/latest/ | Ramirez et al. (2018); Wolff et al. (2020) |
| *hifiasm* | Assembly construction | 0.19.6 | https://github.com/chhylp123/hifiasm | Cheng et al. (2021, 2022) |
| *htslib* | bgzip of vcf files | 1.20 | https://github.com/samtools/htslib | Bonfield et al. (2021) |
| *inspector* | Assembly evaluation | 1.3 | https://github.com/Maggi-Chen/Inspector | Chen et al. 2021 |
| *Juicer* | Construct and visualize HiC contact maps | 1.5 | https://github.com/aidenlab/juicer | Durand et al. (2016) |
| *karyoploteR* | Plot customizable genomes in R | 1.8.4 | https://github.com/bernatgel/karyoploteR | Gel and Serra (2017) |
| *KMC* | Kmer counts | 3.2.4 | https://github.com/refresh-bio/KMC | [Marek Kokot et al. 2017](https://academic.oup.com/bioinformatics/article/33/17/2759/3796399) |
| *Merqury* | Quality, completeness, and phasing assessment | 1.3 | https://github.com/marbl/merqury | Rhie *et al.* 2020 |
| *MitoHiFi* | Mitochondrial genome annotation | 2.9.3 | https://github.com/marcelauliano/MitoHiFi | Uliano-Silva et al. (2023) |
| *minimap2* | Long-read alignments, Alignment for gene model training and prediction | 2.28 | https://github.com/lh3/minimap2 | Li, H. (2018) |
| *RepeatMasker* | Repeat annotations | 4.1.2-p1 | https://github.com/rmhubley/RepeatMasker | Smit et al. (2013-2015) |
| *RepeatModeler* | Repeat annotations | 2.0.4 | https://github.com/Dfam-consortium/RepeatModeler | Smit et al. (2008-2015) |
| *RepeatScout* | Repeat annotation | 1.0.6 | https://github.com/mmcco/RepeatScout | Price et al. (2005) |
| *Samtools* | SAM/BAM viewing, manipulation and calculations | 1.19,1.19.2 | <https://github.com/samtools/samtools> | Danecek et al. (2021) |
| *seqkit* | Toolkit for FastA/FastQ files | 2.5.1 | https://github.com/shenwei356/seqkit | Shen et al. (2016) |
| *seqtk* | Toolkit for FastA/FastQ files | 1.3 | https://github.com/lh3/seqtk | – |
| *SRF* | Satellite Repeat Finder | 9ab3695 | https://github.com/lh3/srf | Zhang et al. 2023 |
| *subread-align* | Align RNAseq to the assembly | 2.0.6 | https://sourceforge.net/projects/subread/ | Liao et al. 2013 |
| *TADbit* | Analysis of HiC data | 1.0.1 | https://github.com/3DGenomes/TADbit | Serra et al. (2017) |
| *TRF* | Tandem repeat annotations including telomeres | 4.09.1 | https://github.com/Benson-Genomics-Lab/TRF | Benson, G. (1999) |
| *trimmomatic* | Trim Illumina sequence data | 0.39 | https://github.com/usadellab/Trimmomatic | Bolger et al., 2014 |
| *Trinity* | Transcriptome assembly | 2.12.0 | https://github.com/trinityrnaseq/trinityrnaseq | Grabherr et al. (2011) |
| *whatshap* | phasing vcf files and HiC reads | 2.3 | https://github.com/whatshap/whatshap | Martin et al. (2016) |
| *YAHS* | Scaffolding with HiC | 1.1 | https://github.com/c-zhou/yahs | Zhou et al. (2023) |

#### **Table S2.** Summary statistics for the raw Illumina RNA sequence data used to assemble the transcriptome and for annotation.

| SpecimenID (UC<Aus>) | Tissue | LibraryID | SRA | No. of Reads | Read Length | Mapped read % |
| --- | --- | --- | --- | --- | --- | --- |
| SW_28ZWC1_2a | Embryonic brain | CAGRF20994.36 | SRR33206849 | 77479290 | 100 | 94.38 |
| SW_28C1_2a | Embryonic brain | CAGRF20994.70 | SRR33206848 | 81974270 | 100 | 94.31 |
| 28ZWC6_2a | Embryonic brain | CAGRF20994.18 | SRR33206842 | 83774333 | 100 | 93.67 |
| SW_3603zz:18:1:19 | Embryonic gonad | CAGRF19863.35 | SRR17842336 | 56313909 | 150 | 82.53 |
| SW_3603zz:18:1:12 | Embryonic gonad | CAGRF19863.28 | SRR17842344 | 56727130 | 150 | 84.47 |
| SW_3632:18:2:1 | Embryonic gonad | CAGRF19863.42 | SRR17842329 | 60050561 | 150 | 85.37 |
| Pit_001003344319 | Heart | POGwqlTABRAAPEI-88 | ERR413072 | 15400726 | 90 | 95.07 |
| Pit_005005002929 | Heart | PW2_GCCAAT_L002 | ERR413078 | 16503039 | 101 | 93.64 |
| Pit_001003348030 | Heart | POGwqlTAHRAAPEI-32 | ERR413065 | 19844224 | 90 | 94.92 |
| Pit_001003344319 | Kidney | 344319kidneyA_FCC1L8RACXX_POGwqlTACRAAPEI-89 | ERR413073 | 12018708 | 90 | 94.71 |
| Pit_001003348030 | Kidney | POGwqlTAIRAAPEI-55 | ERR413066 | 18108164 | 90 | 95.57 |
| Pit_005005002929 | Liver | PW4_GTGAAA_L002 | ERR413080 | 140350855 | 101 | 93.87 |
| Pit_001003344319 | Liver | POGwqlTAARAAPEI-87 | ERR413074 | 164221222 | 90 | 95.70 |
| Pit_001003348030 | Liver | POGwqlTAGRAAPEI-26 | ERR413067 | 16466411 | 90 | 95.74 |
| Pit_001003344319 | Lung | 344319lungA_FCC1L8RACXX_POGwqlTADRAAPEI-90 | ERR413075 | 12205166 | 90 | 95.53 |
| Pit_005005002929 | Lung | PW3_CTTGTA_L002 | ERR413079 | 15418237 | 101 | 92.67 |
| Pit_001003348030 | Lung | POGwqlTAJRAAPEI-61 | ERR413068 | 21179788 | 90 | 96.02 |
| Pit_001003344319 | Skeletal muscle | 344319muscleA_FCC1L8RACXX_POGwqlTAERAAPEI-94 | ERR413076 | 11722151 | 90 | 96.30 |
| Pit_001003348030 | Skeletal muscle | POGwqlTAKRAAPEI-71 | ERR413069 | 18252709 | 90 | 96.82 |
| Pit_005005003588 | Ovary | PW6_ACTTGA_L002 | ERR413082 | 20079842 | 101 | 94.46 |
| Pit_005005002929 | Ovary | PW5_ATCACG_L002 | ERR413081 | 8909165 | 101 | 94.38 |
| Pit_001003348030 | Testis | POGwqlTAMRAAPEI-83 | ERR413070 | 20764245 | 90 | 95.94 |

####

#### **Table S3.** Summary statistics for the raw PacBio HiFi sequence data used for the assembly.

| SpecimenID (UC<Aus>) | Tissue | Flow Cell | SRA | No. of Bases | No. of Reads | Read Length | N50 | N90 |
| --- | --- | --- | --- | --- | --- | --- | --- | --- |
| Pit_001003342236 | Blood | DAGPOG | SRR33206838 | 70,625,888,904 | 4,714,654 | 14,980 | 15,021 | 12,612 |

#### **Table S4.** Summary statistics for the raw Oxford Nanopore sequence data used for the assembly.

| SpecimenID (UC<Aus>) | Tissue | Flow cell | SRA | No. of Bases | No. of Reads | Read Length (bp) | N50 | N90 |
| --- | --- | --- | --- | --- | --- | --- | --- | --- |
| Pit_001003342236 | Blood | PAF09309 | SRR33206836 | 25,283,044,433 | 1,617,304 | 15,633 | 32,645 | 8,495 |
| Pit_001003342236 | Blood | PAF09661 | SRR33206837 | 39,491,153,126 | 3,394,876 | 11,633 | 32,142 | 5,155 |
| Pit_001003342236 | Blood | PAF10280 | SRR33206835 | 13,661,149,861 | 1,040,853 | 13,125 | 33,901 | 5,869 |
| Pit_001003342236 | Blood | PAF14969 | SRR33206845 | 3,934,999,270 | 332,739 | 11,826 | 34,339 | 4,907 |
| Pit_001003342236 | Blood | PAF21165 | SRR33206847 | 13,025,356,130 | 41,0291 | 31,747 | 59,981 | 16,099 |
| Pit_001003342236 | Blood | PAF32809 | SRR33206846 | 10,170,572,213 | 322,452 | 31,541 | 59,310 | 16,562 |

####

#### **Table S5.** Summary statistics for the HiC sequence data used to scaffold the assembly.

| SpecimenID (UC<Aus>) | Tissue | Library | SRA | No. of Bases | No. of Reads | Read Length |
| --- | --- | --- | --- | --- | --- | --- |
| Pit_001003342236 | Blood | 350768_L001 | SRR33206844 | 22,349,129,631 | 148,007,481 | 151 |
| Pit_001003342236 | Blood | 350768_L002 | SRR33206843 | 22,248,518,784 | 14,7341,184 | 151 |

#### **Table S6.** Summary statistics for the Illumina sequence data.

| SpecimenID (UC<Aus>) | Tissue | SRA | No. of Bases | No. of Reads | Mean Read Length |
| --- | --- | --- | --- | --- | --- |
| Pit_001003342236 | Blood | [ERR409918](https://trace.ncbi.nlm.nih.gov/Traces/sra?run=ERR409918), ERR409919, ERR409920 | 46,627,601,425 | 347,200,575 | 134 |

**Table S7.** Bacterial Artificial Chromosome (BAC) sequences mapped to the assembly scaffolds for the bearded dragon *Pogona vitticeps*. These BAC sequences were physically mapped to the chromosomes of the dragon by Deakin et al. (2016) and Young et al. (2013). They serve to confirm the association of the assembly scaffolds with the physical chromosomes. Scaffolds 1-6 correspond to Chromosomes 1-6, both assigned numbers by size. Scaffolds 7-15 correspond to microchromosomes numbered by Deakin et. al. (2016) as indicated. Scaffold 16, the pseudo-autosomal region of the Z and W sex chromosomes, is confirmed to correspond to the Z chromosome in the physical mapping of Clone 150H19. Scaffold 17 is confirmed to be associated with the W chromosome by the anchor Clone C1 of Quinn et al. (2010, Genbank EU938138).

| **BAC ID** | **Chromosome** | **Scaffold** | **Start** | **End** | **Pairing** | **Length (bp)** |
| --- | --- | --- | --- | --- | --- | --- |
| 16A1 | 1 | 1 | 123,407,376 | 123,573,280 | Double End | 165904 |
| 16A9 | 1 | 1 | 206,788,482 | 206,922,132 | Double End | 133650 |
| 57H2 | 1 | 1 | 23,855,452 | 23,978,560 | Double End | 123108 |
| 170F19 | 1 | 1 | 264,497,932 | 264,639,501 | Double End | 141569 |
| 184J20 | 1 | 1 | 273,284,146 | 273,381,615 | Double End | 97469 |
| 220D11 | 1 | 1 | 269,856,714 | 269,989,320 | Double End | 132606 |
| 220D7 | 1 | 1 | 198,454,349 | 198,556,275 | Double End | 101926 |
| 229E3 | 1 | 1 | 333,283,458 | 333,284,262 | Single End | 804 |
| 16A12 | 1 | 1 | 196,202,148 | 196,307,065 | Double End | 104917 |
| 11A17 | 1 | 1 | 157,690,828 | 157,788,565 | Double End | 97737 |
| 63H9 | 1 | 1 | 87,094,079 | 87,094,329 | Single End | 250 |
| 55I5 | 1 | 1 | 241,422,170 | 241,423,063 | Single End | 893 |
| 50I5 | 1 | 1 | 241,629,475 | 241,630,352 | Single End | 877 |
| 32C14 | 1 | 1 | 251,475,667 | 251,576,845 | Double End | 101178 |
| 31C8 | 1 | 1 | 159,024,102 | 159,024,242 | Single End | 140 |
| 240M14 | 1 | 1 | 219,470,320 | 219,470,616 | Single End | 296 |
| 191F7 | 1 | 1 | 302,271,442 | 302,349,405 | Double End | 77963 |
| 166M7 | 1 | 1 | 177,617,052 | 177,734,321 | Double End | 117269 |
| 161K22 | 1 | 1 | 312,800,053 | 312,800,696 | Single End | 643 |
| 12E8 | 1 | 1 | 275,882,655 | 275,883,474 | Single End | 819 |
| 57C5 | 1 | 1 | 113,655,489 | 113,656,282 | Single End | 793 |
| 28K11 | 1 | 1 | 233,289,187 | 233,289,570 | Single End | 383 |
| 231C17 | 1 | 1 | 139,946,455 | 139,946,593 | Single End | 138 |
| 167F2 | 1 | 1 | 189,870,499 | 189,871,006 | Single End | 507 |
| 16A23 | 2 | 2 | 66,988,935 | 67,151,660 | Double End | 162725 |
| 16A4 | 2 | 2 | 253,796,445 | 253,948,084 | Double End | 151639 |
| 176E5 | 2 | 2 | 211,772,680 | 211,923,226 | Double End | 150546 |
| 189J12 | 2 | 2 | 143,064,625 | 143,210,897 | Double End | 146272 |
| 195K1 | 2 | 2 | 166,137,002 | 166,292,352 | Double End | 155350 |
| 200H9 | 2 | 2 | 166,111,395 | 166,264,490 | Double End | 153095 |
| 219G15 | 2 | 2 | 39,640,903 | 39,810,574 | Double End | 169671 |
| 238E7 | 2 | 2 | 39,702,429 | 39,859,488 | Double End | 157059 |
| 203J2 | 2 | 2 | 146,656,877 | 146,757,171 | Double End | 100294 |
| 119M24 | 2 | 2 | 28,575,669 | 28,661,793 | Double End | 86124 |
| 76H7 | 2 | 2 | 301,486,841 | 301,597,372 | Double End | 110531 |
| 63C12 | 2 | 2 | 28,669,241 | 28,669,816 | Single End | 575 |
| 52J6 | 2 | 2 | 217,609,420 | 217,729,471 | Double End | 120051 |
| 21F11 | 2 | 2 | 93,857,250 | 93,857,797 | Single End | 547 |
| 213G6 | 2 | 2 | 139,763,286 | 139,763,386 | Single End | 100 |
| 160F4 | 2 | 2 | 29,206,132 | 29,283,406 | Double End | 77274 |
| 153E7 | 2 | 2 | 23,679,237 | 23,797,983 | Double End | 118746 |
| 74F13 | 2 | 2 | 77,615,637 | 77,615,778 | Single End | 141 |
| 57B1 | 2 | 2 | 123,317,551 | 123,318,392 | Single End | 841 |
| 42J16 | 2 | 2 | 203,443,365 | 203,443,969 | Single End | 604 |
| 225M3 | 2 | 2 | 4,920,160 | 4,920,229 | Single End | 69 |
| 104C4 | 2 | 2 | 31,514,680 | 31,515,011 | Single End | 331 |
| 185A1 | 3 | 3 | 228,674,547 | 228,798,733 | Double End | 124186 |
| 213B13 | 3 | 3 | 161,338,667 | 161,452,623 | Double End | 113956 |
| 214J17 | 3 | 3 | 154,656,138 | 154,797,939 | Double End | 141801 |
| 220D15 | 3 | 3 | 132,974,112 | 133,117,342 | Double End | 143230 |
| 221A23 | 3 | 3 | 72,300,526 | 72,406,473 | Double End | 105947 |
| 233A1 | 3 | 3 | 259,774,934 | 259,868,300 | Double End | 93366 |
| 71H17 | 3 | 3 | 212,191,524 | 212,191,947 | Single End | 423 |
| 60E8 | 3 | 3 | 101,809,810 | 101,809,872 | Single End | 62 |
| 41K6 | 3 | 3 | 263,235,206 | 263,235,636 | Single End | 430 |
| 224N13 | 3 | 3 | 54,477,440 | 54,572,033 | Double End | 94593 |
| 212O13 | 3 | 3 | 28,075,418 | 28,172,637 | Double End | 97219 |
| 98J10 | 3 | 3 | 48,936,383 | 49,023,996 | Double End | 87613 |
| 70H15 | 3 | 3 | 89,843,258 | 89,843,367 | Single End | 109 |
| 141N2 | 3 | 3 | 207,206,868 | 207,207,300 | Single End | 432 |
| 16A5 | 4 | 4 | 223,343,607 | 223,475,138 | Double End | 131531 |
| 219I19 | 4 | 4 | 15,956,020 | 16,099,868 | Double End | 143848 |
| 219N21 | 4 | 4 | 212,509,566 | 212,656,462 | Double End | 146896 |
| 230L10 | 4 | 4 | 30,902,447 | 31,039,140 | Double End | 136693 |
| 240P5 | 4 | 4 | 217,448,160 | 217,604,952 | Double End | 156792 |
| 94G20 | 4 | 4 | 131,828,670 | 131,828,800 | Single End | 130 |
| 55L2 | 4 | 4 | 35,486,665 | 35,486,806 | Single End | 141 |
| 39P7 | 4 | 4 | 22,140,696 | 22,140,795 | Single End | 99 |
| 240G13 | 4 | 4 | 70,024,998 | 70,025,094 | Single End | 96 |
| 6M2 | 4 | 4 | 46,157,770 | 46,158,228 | Single End | 458 |
| 47D22 | 4 | 4 | 50,742,605 | 50,742,749 | Single End | 144 |
| 43K4 | 4 | 4 | 197,338,787 | 197,338,848 | Single End | 61 |
| 152L3 | 4 | 4 | 44,449,933 | 44,450,480 | Single End | 547 |
| 16A22 | 5 | 5 | 21,793,409 | 21,907,879 | Double End | 114470 |
| 16A3 | 5 | 5 | 77,173,589 | 77,287,486 | Double End | 113897 |
| 210E16 | 5 | 5 | 191,209,567 | 191,327,937 | Double End | 118370 |
| 220D13 | 5 | 5 | 90,196,139 | 90,319,940 | Double End | 123801 |
| 233L23 | 5 | 5 | 191,071,018 | 191,295,551 | Double End | 224533 |
| 127M2 | 5 | 5 | 75,255,243 | 75,255,595 | Single End | 352 |
| 64B22 | 5 | 5 | 32,602,157 | 32,602,461 | Single End | 304 |
| 213P24 | 5 | 5 | 105,780,143 | 105,780,819 | Single End | 676 |
| 106M24 | 5 | 5 | 112,608,113 | 112,608,684 | Single End | 571 |
| 16A11 | 6 | 6 | 48,179,595 | 48,324,927 | Double End | 145332 |
| 132P11 | 6 | 6 | 98,533,797 | 98,652,352 | Double End | 118555 |
| 174P24 | 6 | 6 | 40,475,783 | 40,599,680 | Double End | 123897 |
| 200O10 | 6 | 6 | 115,445,513 | 115,564,715 | Double End | 119202 |
| 211I19 | 6 | 6 | 6,747,727 | 6,748,354 | Single End | 627 |
| 212P4 | 6 | 6 | 91,758,787 | 91,899,479 | Double End | 140692 |
| 225A2 | 6 | 6 | 113,902,256 | 114,060,808 | Double End | 158552 |
| 58O7 | 6 | 6 | 85,615,842 | 85,616,283 | Single End | 441 |
| 64E23 | 6 | 6 | 23,949,286 | 23,949,848 | Single End | 562 |
| 197P21 | 7 | 7 | 3,381,866 | 3,526,151 | Double End | 144285 |
| 75B13 | 7 | 7 | 4,323,835 | 4,324,297 | Single End | 462 |
| 163B7 | 7 | 7 | 7,515,270 | 7,515,386 | Single End | 116 |
| 55E1 | 7 | 7 | 27,269,115 | 27,269,479 | Single End | 364 |
| 220D8 | 11 | 8 | 19,977,289 | 20,074,547 | Double End | 97258 |
| 60I7 | Unplaced | 9 | 10,399,195 | 10,399,466 | Single End | 271 |
| 105P18 | 8 | 10 | 14,907,609 | 15,070,287 | Double End | 162678 |
| 232P19 | 8 | 10 | 3,325,360 | 3,419,255 | Double End | 93895 |
| 90K22 | 8 | 10 | 6,519,310 | 6,519,416 | Single End | 106 |
| 220D12 | 10 | 11 | 5,805,476 | 5,892,623 | Double End | 87147 |
| 161M1 | 10 | 11 | 25,752,319 | 25,752,921 | Single End | 602 |
| 188M22 | 11? | 12 | 3,335,567 | 3,499,827 | Double End | 164260 |
| 26E15 | 9 | 12 | 24,695,378 | 24,696,082 | Single End | 704 |
| 221B16 | 12 | 13 | 3,354,875 | 3,470,616 | Double End | 115741 |
| 39B11 | Unplaced | 13 | 9,469,199 | 9,469,292 | Single End | 93 |
| 214G3 | 14 | 14 | 13,829,067 | 13,993,455 | Double End | 164388 |
| 104H17 | Unknown micro | 14 | 15,328,359 | 15,328,421 | Single End | 62 |
| 16A10 | 15 | 15 | 7,185,376 | 7,387,041 | Double End | 201665 |
| 185N3 | 13 | 15 | 2,286,971 | 2,409,964 | Double End | 122993 |
| 240C19 | 13 | 15 | 1,448,843 | 1,448,947 | Single End | 104 |
| 218G5 | Unknown micro | 16 (PAR) | 3,169,270 | 3,169,542 | Single End | 272 |
| 150H19 | Z | 16 (PAR) | 3,169,270 | 3,169,542 | Single End | 272 |
| Contig C | Unplaced | 17 | 2,268,844 | 2,271,580 | -- | 2,736 |
| 57P12 | Unknown micro | 18 | 2,381,218 | 2,381,349 | Single End | 131 |

**Table S8.** Satellite repeat units of the genome assembly for the bearded dragon *Pogona vitticeps* collapsed into 45 distinct classes based on sequence similarity.

| Satellite Repeat Name | Class | Repeat Unit Length (bp) |
| --- | --- | --- |
| POGVIT.v2.1#circ15-93 | srfclass-1 | 93 |
| POGVIT.v2.1#circ19-93 | srfclass-1 | 93 |
| POGVIT.v2.1#circ56-93 | srfclass-1 | 93 |
| POGVIT.v2.1#circ47-93 | srfclass-1 | 93 |
| POGVIT.v2.1#circ54-93 | srfclass-1 | 93 |
| POGVIT.v2.1#circ45-93 | srfclass-1 | 93 |
| POGVIT.v2.1#circ43-93 | srfclass-1 | 93 |
| POGVIT.v2.1#circ55-93 | srfclass-1 | 93 |
| POGVIT.v2.1#circ35-93 | srfclass-1 | 93 |
| POGVIT.v2.1#circ39-93 | srfclass-1 | 93 |
| POGVIT.v2.1#circ23-93 | srfclass-1 | 93 |
| POGVIT.v2.1#circ60-93 | srfclass-1 | 93 |
| POGVIT.v2.1#circ9-167 | srfclass-2 | 167 |
| POGVIT.v2.1#circ24-166 | srfclass-2 | 166 |
| POGVIT.v2.1#circ33-169 | srfclass-2 | 169 |
| POGVIT.v2.1#circ44-166 | srfclass-2 | 166 |
| POGVIT.v2.1#circ4-128 | srfclass-3 | 128 |
| POGVIT.v2.1#circ11-129 | srfclass-3 | 129 |
| POGVIT.v2.1#circ49-129 | srfclass-3 | 129 |
| POGVIT.v2.1#circ5-98 | srfclass-4 | 98 |
| POGVIT.v2.1#circ10-98 | srfclass-4 | 98 |
| POGVIT.v2.1#circ8-151 | srfclass-5 | 151 |
| POGVIT.v2.1#circ22-151 | srfclass-5 | 151 |
| POGVIT.v2.1#circ17-115 | srfclass-6 | 115 |
| POGVIT.v2.1#circ27-115 | srfclass-6 | 115 |
| POGVIT.v2.1#circ20-161 | srfclass-7 | 161 |
| POGVIT.v2.1#circ41-161 | srfclass-7 | 161 |
| POGVIT.v2.1#circ32-83 | srfclass-8 | 83 |
| POGVIT.v2.1#circ50-44 | srfclass-8 | 44 |
| POGVIT.v2.1#circ52-63 | srfclass-9 | 63 |
| POGVIT.v2.1#circ57-63 | srfclass-9 | 63 |
| POGVIT.v2.1#circ1-6 | srfclass-10 | 6 |
| POGVIT.v2.1#circ2-5695 | srfclass-11 | 5,695 |
| POGVIT.v2.1#circ3-151 | srfclass-12 | 151 |
| POGVIT.v2.1#circ6-150 | srfclass-13 | 150 |
| POGVIT.v2.1#circ7-151 | srfclass-14 | 151 |
| POGVIT.v2.1#circ12-398 | srfclass-15 | 398 |
| POGVIT.v2.1#circ13-157 | srfclass-16 | 157 |
| POGVIT.v2.1#circ14-230 | srfclass-17 | 230 |
| POGVIT.v2.1#circ16-9460 | srfclass-18 | 9,460 |
| POGVIT.v2.1#circ18-175 | srfclass-19 | 175 |
| POGVIT.v2.1#circ21-6 | srfclass-20 | 6 |
| POGVIT.v2.1#circ25-2190 | srfclass-21 | 2,190 |
| POGVIT.v2.1#circ26-115 | srfclass-22 | 115 |
| POGVIT.v2.1#circ28-249 | srfclass-23 | 249 |
| POGVIT.v2.1#circ29-179 | srfclass-24 | 179 |
| POGVIT.v2.1#circ30-236 | srfclass-25 | 236 |
| POGVIT.v2.1#circ31-5 | srfclass-26 | 5 |
| POGVIT.v2.1#circ34-144 | srfclass-27 | 144 |
| POGVIT.v2.1#circ36-150 | srfclass-28 | 150 |
| POGVIT.v2.1#circ37-102 | srfclass-29 | 102 |
| POGVIT.v2.1#circ38-3674 | srfclass-30 | 3,674 |
| POGVIT.v2.1#circ40-252 | srfclass-31 | 252 |
| POGVIT.v2.1#circ42-145 | srfclass-32 | 145 |
| POGVIT.v2.1#circ46-130 | srfclass-33 | 130 |
| POGVIT.v2.1#circ48-5 | srfclass-34 | 5 |
| POGVIT.v2.1#circ51-6 | srfclass-35 | 6 |
| POGVIT.v2.1#circ53-171 | srfclass-36 | 171 |
| POGVIT.v2.1#circ58-104 | srfclass-37 | 104 |
| POGVIT.v2.1#circ59-877 | srfclass-38 | 877 |
| POGVIT.v2.1#circ61-5 | srfclass-39 | 5 |
| POGVIT.v2.1#circ62-5 | srfclass-40 | 5 |
| POGVIT.v2.1#circ63-6 | srfclass-41 | 6 |
| POGVIT.v2.1#circ64-152 | srfclass-42 | 152 |
| POGVIT.v2.1#circ65-128 | srfclass-43 | 128 |
| POGVIT.v2.1#circ66-54 | srfclass-44 | 54 |
| POGVIT.v2.1#circ67-5 | srfclass-45 | 5 |

**Table S9.** Summary of the copy number and percentage of the bearded dragon (*Pogona vitticeps*) genome covered by repeat elements.

| **Family** | | **Numbers of elements** | **Length masked (bp)** | **% of sequence** |
| --- | --- | --- | --- | --- |
| **Retroelements** | | **1,061,885** | **303,713,020** | **17.33** |
| SINEs | | 114,787 | 15,769,671 | 0.90 |
|  | 5S | 6,625 | 553,278 | 0.03 |
|  | ID | 3,444 | 310,000 | 0.02 |
|  | MIR | 36,494 | 5,075,373 | 0.29 |
|  | U | 346 | 11,467 | 0.00 |
|  | U-L1 | 82 | 5,430 | 0.00 |
|  | tRNA | 15,532 | 1,687,441 | 0.10 |
|  | tRNA-Core-RTE | 8,936 | 319,829 | 0.02 |
|  | tRNA-Deu | 39,885 | 7,574,891 | 0.43 |
|  | tRNA-RTE | 3,443 | 231,962 | 0.01 |
| LINEs | | 947,098 | 287,943,349 | 16.43 |
|  | CR1 | 178,263 | 39,822,603 | 2.27 |
|  | Dong-R4 | 21,177 | 6,300,007 | 0.36 |
|  | I | 649 | 44,872 | 0.00 |
|  | I-Jockey | 16,536 | 3,023,508 | 0.17 |
|  | L1 | 32,568 | 15,027,310 | 0.86 |
|  | L2 | 199,361 | 64,273,433 | 3.67 |
|  | Penelope | 66,521 | 13,708,273 | 0.78 |
|  | R2-NeSL | 116 | 22,059 | 0.00 |
|  | RTE-BovB | 372,010 | 129,982,167 | 7.42 |
|  | RTE-RTE | 269 | 64,349 | 0.00 |
|  | RTE-X | 50,133 | 11,827,799 | 0.67 |
|  | Rex-Babar | 9,495 | 3,846,969 | 0.22 |
|  |  |  |  |  |
| **LTR elements** | | **80,454** | **72,706,738** | **4.15** |
|  | Copia | 3,713 | 3,224,584 | 0.18 |
|  | DIRS | 25,270 | 16,851,664 | 0.96 |
|  | ERV | 272 | 104,097 | 0.01 |
|  | ERV1 | 5,776 | 1,596,256 | 0.09 |
|  | ERVK | 1,795 | 996,283 | 0.06 |
|  | Gypsy | 27,479 | 42,285,692 | 2.41 |
|  | Ngaro | 16,149 | 7,648,162 | 0.44 |
|  |  |  |  |  |
| **DNA transposons** | | **432,771** | **89,795,030** | **5.12** |
|  | CMC-Chapaev-3 | 385 | 154,910 | 0.01 |
|  | Maverick | 871 | 618,150 | 0.04 |
|  | PIF-Harbinger | 5,195 | 676,377 | 0.04 |
|  | TcMar | 58,667 | 18,056,702 | 1.03 |
|  | TcMar-Mariner | 2,350 | 1,083,990 | 0.06 |
|  | TcMar-Tc1 | 10,032 | 4,721,161 | 0.27 |
|  | TcMar-Tc2 | 120,600 | 15,583,345 | 0.89 |
|  | TcMar-Tigger | 120,825 | 27,876,875 | 1.59 |
|  | Zisupton | 1,384 | 316,647 | 0.02 |
|  | hAT-Ac | 24,713 | 4,868,609 | 0.28 |
|  | hAT-Blackjack | 6,949 | 1,247,319 | 0.07 |
|  | hAT-Charlie | 61,120 | 10,465,252 | 0.60 |
|  | hAT-Tag1 | 7,783 | 1,336,798 | 0.08 |
|  | hAT-Tip100 | 11,161 | 2,464,145 | 0.14 |
|  | hAT-hAT19 | 237 | 113,591 | 0.01 |
|  | hAT-hAT5 | 499 | 211,159 | 0.01 |
|  |  |  |  |  |
| **Penelope-like elements** | | **1,797** | **150,905** | **0.01** |
|  | Chlamys | 1,797 | 150,905 | 0.01 |
|  |  |  |  |  |
| **Rolling-circles** | | **447** | **95,044** | **0.01** |
|  | Helitron | 447 | 95,044 | 0.01 |
|  |  |  |  |  |
| **Unclassified** | | **1,775,114** | **294,309,581** | **16.79** |
|  |  |  |  |  |
| **Total interspersed repeats** | | **3,352,468** | **760,770,318** | **43.40** |
|  |  |  |  |  |
| Satellite | | 4,246 | 1,284,141 | 0.07 |
| Simple Repeat | | 576,326 | 34,276,116 | 1.96 |
| rRNA | | 402 | 1,812,567 | 0.10 |
| snRNA | | 2,397 | 502,447 | 0.03 |
| tRNA | | 101 | 6,775 | 0.00 |
|  |  |  |  |  |
| **Total Masked** | |  | 798,652,364 | 45.56 |


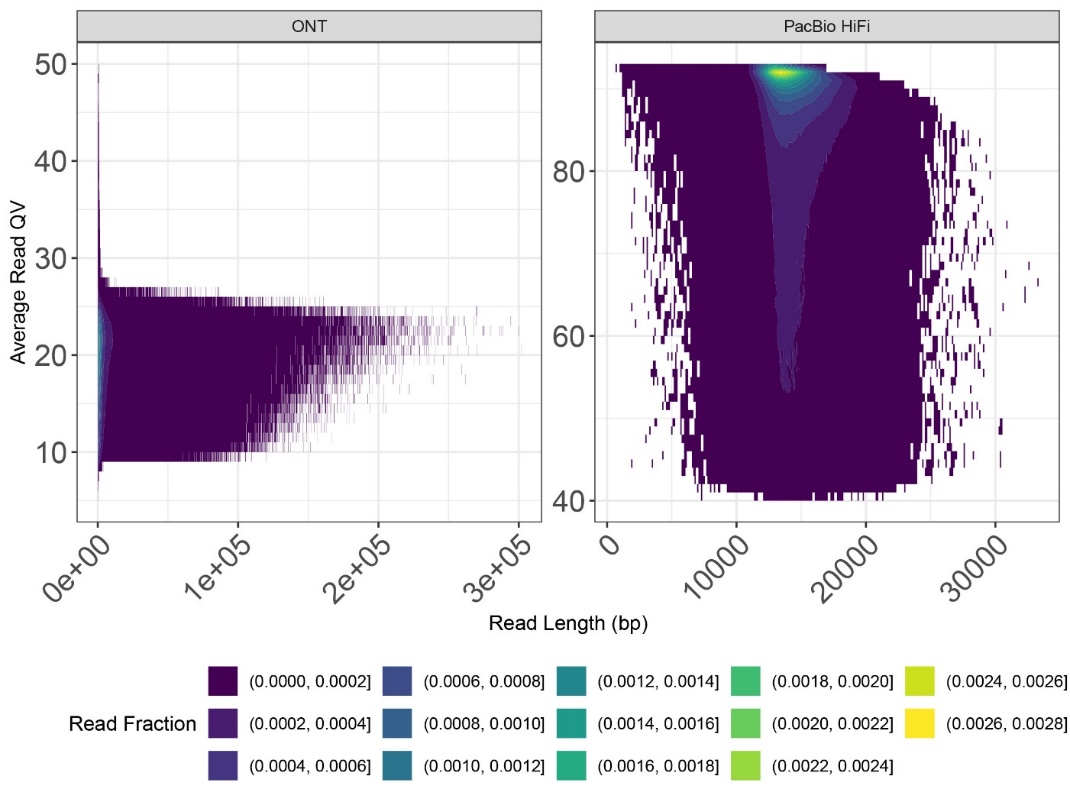


**Figure S1.** Comparison of average read quality values (QV) versus read length for the two sequencing technologies: Oxford Nanopore Technologies (ONT) and PacBio HiFi. Color intensity represents the base fraction in specified ranges, with darker colors indicating lower fractions and lighter colors indicating higher fractions. ONT reads show a broader distribution of read lengths with moderate quality values, whereas PacBio HiFi reads exhibit higher quality values with more concentrated reads at ~15 Kbp lengths.


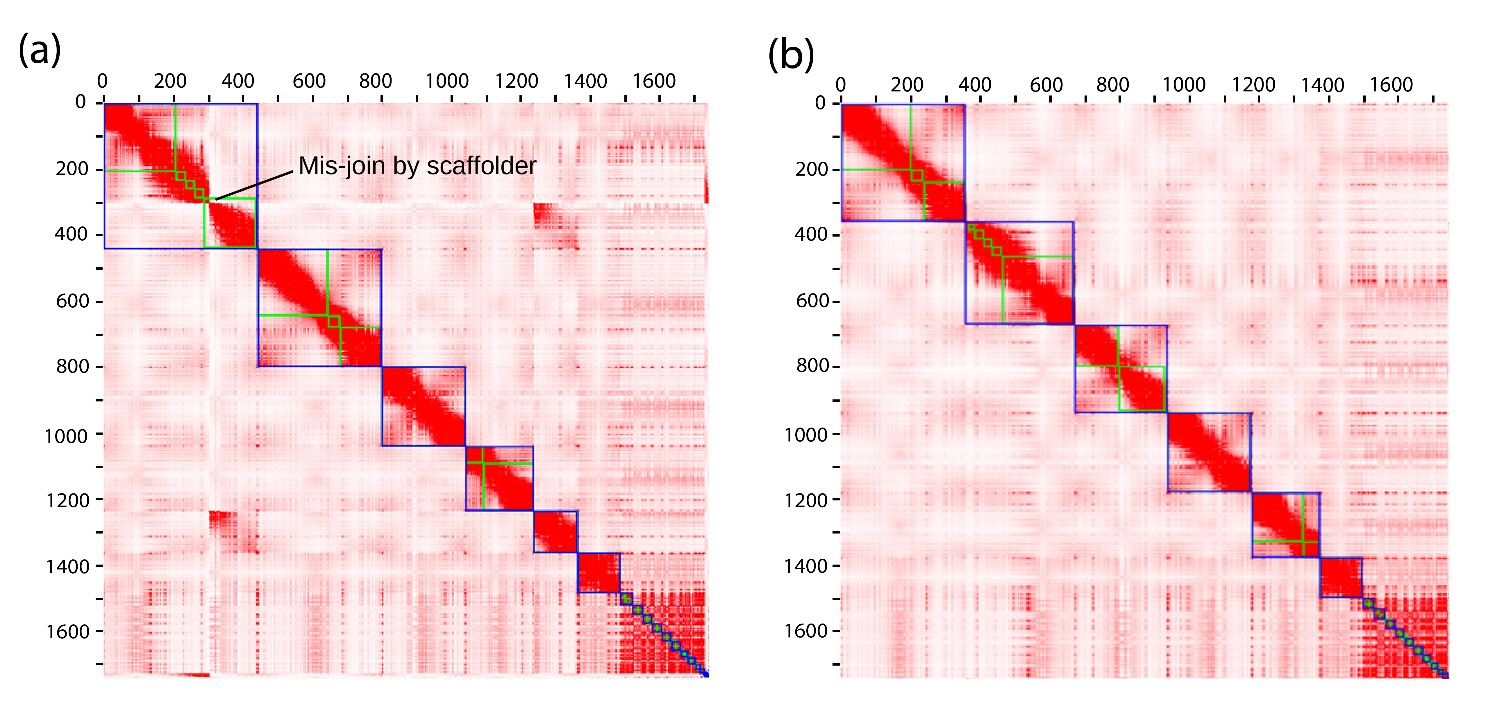


**Figure S2.** HiC contact maps for Haplotype 2 showing an assembly mis-join in the YAHS assembly. **(a)** The original contact map showing the mis-join; **(b)** the resolved assembly with the mis-join was resolved manually.


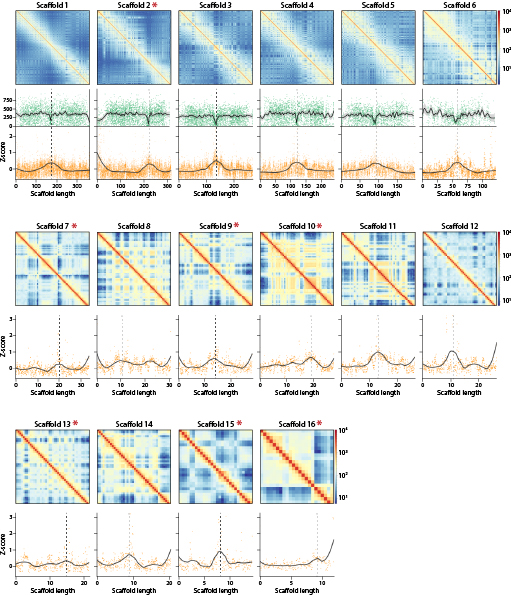


**Figure S3.** Identification of putative centromeres for the six macrochromosomes and 10 microchromosomes of the bearded dragon *Pogona vitticeps*. For both macrochromosomes and microchromosomes, the upper panels are chromosome-specific Hi-C heatmaps showing intra-chromosomal interactions. The lower panels are the Z-scores for the HiC inter-chromosomal interactions along chromosome length (Mbp) with smoothed lines of best fit. Each dot in the lower panels represents the Z-score interaction value of a different 50 Kbp bin. The middle panels for the macrochromosomes only, show the counts of heterozygous sites per 50 Kbp window (green dots) with lines of best fit and 95% confidence interval (grey shading). Dashed vertical lines correspond to putative centromere locations. Scaffold 16 is the pseudo-autosomal region (PAR) of the sex chromosomes. Scaffolds marked with an asterisk are inverted with respect to the published karyotype.


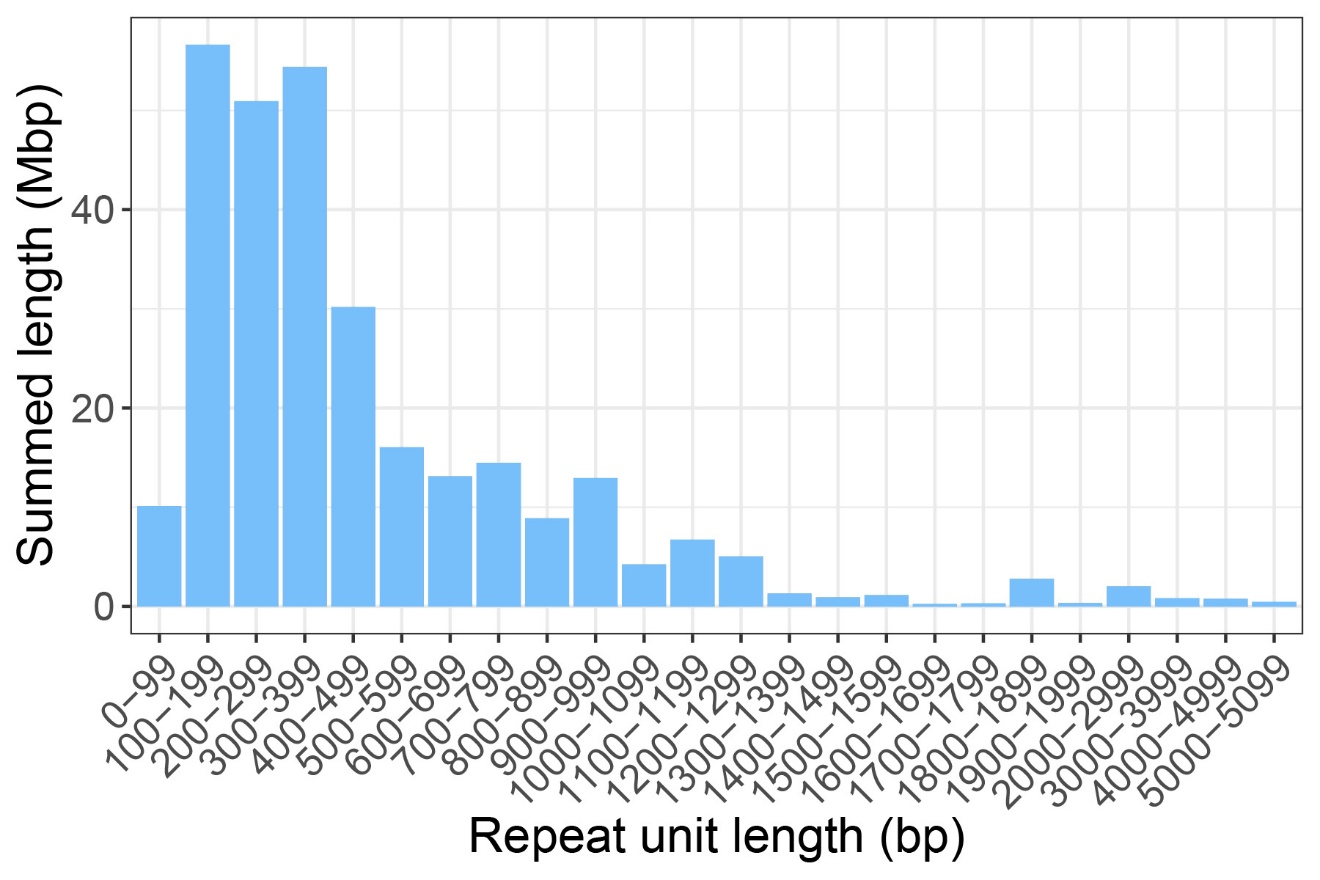


**Figure S4**. Size distribution of the repetitive elements that could not be identified (16.8%, Table S8).


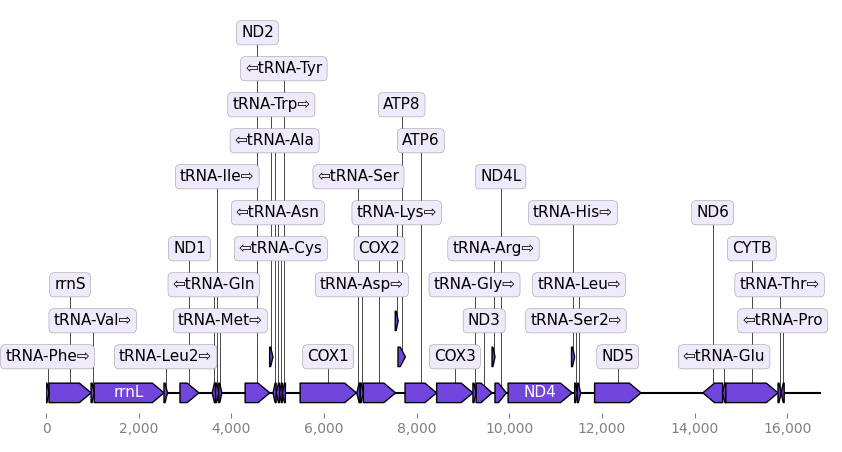


**Figure S5**. Annotation of the mitochondrial genome of the bearded dragon *Pogona vitticeps* assembled using *flye* and annotated using *mitoHiFi*. Control region not shown. Length 16,731 bp.

## References

Price, A.L., Jones, N.C., Pevzner, P.A. 2005. *De novo* identification of repeat families in large genomes. *Bioinformatics* 21: i351–i358. [https://doi.org/10.1093/bioinformatics/bti1018.](https://doi.org/10.1093/bioinformatics/bti1018)

Benson G. 1999. Tandem repeats finder: a program to analyze DNA sequences. *Nucleic Acids Research* 27: 573–580. [https://doi.org/10.1093/nar/27.2.573.](https://academic.oup.com/nar/article/27/2/573/1061099)

Bolger, A.M., Lohse, M., Usadel, B. 2014. Trimmomatic: a flexible trimmer for Illumina sequence data. *Bioinformatics* 30:2114–2120. <https://doi.org/10.1093/bioinformatics/btu170.>

Buchfink, B., Reuter, K., Drost, H.G. 2021. Sensitive protein alignments at tree-of-life scale using DIAMOND. Nature Methods 18:366–368. [https://doi.org/10.1038/s41592-021-01101-x.](https://doi.org/10.1038/s41592-021-01101-x)

Camacho, C., Coulouris, G., Avagyan, V., Ma, N., Papadopoulos, J., Bealer, K., Madden, T.L., 2009. BLAST+: architecture and applications. *BMC Bioinformatics* 10: 421.

Chen, Y., Zhang, Y., Wang, A.Y. [Gao,](https://genomebiology.biomedcentral.com/articles/10.1186/s13059-021-02527-4#auth-Min-Gao-Aff2-Aff5) M., [Chong,](https://genomebiology.biomedcentral.com/articles/10.1186/s13059-021-02527-4#auth-Zechen-Chong-Aff1-Aff2) Z. 2021. Accurate long-read de novo assembly evaluation with Inspector. Genome Biology 22: 312. https://doi.org/10.1186/s13059-021-02527-4.

Cheng, H., Concepcion, G.T., Feng, X. et al. 2021. Haplotype-resolved de novo assembly using phased assembly graphs with hifiasm. *Nature Methods* 18: 170–175. <https://doi.org/10.1038/s41592-020-01056-5.>

Cheng, H., Jarvis, E.D., Fedrigo, O., Koepfli, K.P., Urban, L., Gemmell, N.J., Li, H. 2022. Haplotype-resolved assembly of diploid genomes without parental data. *Nature Biotechnology* 40: 1332–1335. [https://doi.org/10.1038/s41587-022-01261-x.](https://doi.org/10.1038/s41587-022-01261-x)

Cock, P.J.A., Antao, T., Chang, J.T., Chapman, B.A., Cox, C.J., Dalke, A., Friedberg, I., Hamelryck, T., Kauff, F., Wilczynski, B., de Hoon, M.J.L. 2009. Biopython: freely available Python tools for computational molecular biology and bioinformatics, *Bioinformatics* 25: 1422–1423.<https://doi.org/10.1093/bioinformatics/btp163>.

Danecek, P., Bonfield, J.K., Liddle, J., Marshall, J., Ohan, V., Pollard, M.O., Whitwham, A., Keane, T., McCarthy, S.A., Davies, R.M., Li, H. 2021. Twelve years of SAMtools and BCFtools. *GigaScience* 10: giab008. <https://doi.org/10.1093/gigascience/giab008.>

Dainat, J. 2022. Another Gtf/Gff Analysis Toolkit (AGAT): Resolve interoperability issues and accomplish more with your annotations. Plant and Animal Genome XXIX Conference. https://github.com/NBISweden/AGAT.

Dudchenko, O., Batra, S.S., Omer, A.D., Nyquist, S.K., Hoeger, M., Durand, N.C., Shamim, M.S., Machol, I., Lander, E.S., Aiden, A.P., Aiden, E.L. 2017. De novo assembly of the *Aedes aegypti* genome using Hi-C yields chromosome-length scaffolds*.* *Science* 356:92-95. https://doi.org/<https://doi.org/10.1126/science.aal3327>.

Durand, N.C., Shamim, M.S., Machol, I., Rao, S.S.P., Huntley, M.H., Lander, E.S., Aiden, E.L. 2016. Juicer provides a one-click system for analyzing loop-resolution Hi-C experiments. *Cell Systems* 3: 95-98. https://doi.org/10.1016/j.cels.2016.07.002.

Edwards, R.J., Dong, C., Park, R.F., Tobias, P.A. 2022. A phased chromosome-level genome and full mitochondrial sequence for the dikaryotic myrtle rust pathogen, *Austropuccinia psidii*. *bioRxiv* 2022.04.22.489119 https://doi.org/[10.1101/2022.04.22.489119](https://doi.org/10.1101/2022.04.22.489119)

Fu, L., Niu, B., Zhu, Z., Wu, S., Li, W. 2013. CD-HIT: accelerated for clustering the next-generation sequencing data. *Bioinformatics*. 28: 3150-152. https://doi.org/10.1093/bioinformatics/bts565.

Garrison, E., Marth, G. 2012. Haplotype-based variant detection from short-read sequencing. *arXiv preprint arXiv:1207.3907 [q-bio.GN].*

Gel, B., Serra, E. 2017. karyoploteR: an R/Bioconductor package to plot customizable genomes displaying arbitrary data, *Bioinformatics* 33: 3088–3090. [https://doi.org/10.1093/bioinformatics/btx346.](https://doi.org/10.1093/bioinformatics/btx346)

Grabherr, M.G., Haas, B.J., Yassour, M., Levin, J.Z., Thompson, D..A, Amit, I., Adiconis, X, Fan L., Raychowdhury, R., Zeng, Q., Chen, Z., Mauceli, E., Hacohen, N., Gnirke, A., Rhind, N., di Palma, F., Birren, B.W., Nusbaum, C., Lindblad-Toh, K., Friedman, N., Regev, A. 2011. Full-length transcriptome assembly from RNA-seq data without a reference genome. *Nature Biotechnology* 29: 644-52. https://doi.org/10.1038/nbt.1883.

Gremme, G., Steinbiss, S., Kurtz, S. 2013. GenomeTools: a comprehensive software library for efficient processing of structured genome annotations. *IEEE/ACM Trans Computational Biology and Bioinformatics*10: 645-656. https://doi.org/10.1109/TCBB.2013.68.

Kokot, M., Długosz, M., Deorowicz, S. 2017. KMC 3: counting and manipulating k-mer statistics. *Bioinformatics* 33: 2759–2761. https://doi.org/10.1093/bioinformatics/btx304.

Olmogorov, M., Yuan, J., Lin, Y., Pevzner, P. 2019. Assembly of long error-prone reads using repeat graphs. *Nature Biotechnology* 37: 540-546. [https://doi.org/10.1038/s41587-019-0072-8.](https://doi.org/10.1038/s41587-019-0072-8)

Li, H. 2018. Minimap2: pairwise alignment for nucleotide sequences. *Bioinformatics* 34:3094–3100. <https://doi.org/10.1093/bioinformatics/bty191>

Liao, Y., Smyth, G.K., Shi, W. 2013. The Subread aligner: fast, accurate and scalable read mapping by seed-and-vote. *Nucleic Acids Research* 41 :e108.

[Manni](https://currentprotocols.onlinelibrary.wiley.com/authored-by/Manni/Mos%C3%A8), M., [Berkeley](https://currentprotocols.onlinelibrary.wiley.com/authored-by/Berkeley/Matthew+R.), M.R., [Seppey](https://currentprotocols.onlinelibrary.wiley.com/authored-by/Seppey/Mathieu), M., Zdobnov, E.M. 2021. BUSCO: Assessing genomic data quality and beyond. *Current Protocols* <https://doi.org/10.1002/cpz1.323.>

Marco-Sola, S., Sammeth, M., Guigó, R., Ribeca, P. 2012. The GEM mapper: Fast, accurate and versatile alignment by filtration. *Nature Methods* 9: 1185–1188. <https://doi.org/10.1038/nmeth.2221>

Martin, M., Patterson, M., Garg, S., Fischer, S.O., Pisanti, N., Klau, G.W., Schoenhuth, A., Marschall, T. 2016. *WhatsHap:* fast and accurate read-based phasing. *bioRxiv* 085050. https://doi.org/[10.1101/085050](https://doi.org/10.1101/085050)

Price, A.L., Jones, N.C., Pevzner, P.A. 2005. *De novo* identification of repeat families in large genomes. *Bioinformatics* 21: i351–i358.[https://doi.org/10.1093/bioinformatics/bti1018.](https://doi.org/10.1093/bioinformatics/bti1018)

Ramírez, F., Bhardwaj, V., Arrigoni, L., Lam, K. C., Grüning, B.A., Villaveces, J., Habermann, B., Akhtar, A., Manke, T. 2018. High-resolution TADs reveal DNA sequences underlying genome organization in flies. *Nature Communications* 9: 189. <https://doi.org/10.1038/s41467-017-02525-w>

Rhie, A., Walenz, B.P., Koren, S., [Phillippy,](https://genomebiology.biomedcentral.com/articles/10.1186/s13059-020-02134-9#auth-Adam_M_-Phillippy-Aff1) A.M.. 2020. Merqury: reference-free quality, completeness, and phasing assessment for genome assemblies. *Genome Biology* 21 :245. <https://doi.org/10.1186/s13059-020-02134-9.>

Serra, F., Baù, D., Goodstadt, M., Castillo, D., Filion, G.J., Marti-Renom, M.A. 2017. Automatic analysis and 3D-modelling of Hi-C data using TADbit reveals structural features of the fly chromatin colors. *PLoS Computational Biology* 13: e1005665. <https://doi.org/10.1371/journal.pcbi.1005665.>

Shen, W., Le, S., Li, Y., Hu, F. 2016. SeqKit: A cross-platform and ultrafast toolkit for FASTA/Q file manipulation. *PLoS ONE* 11(10): e0163962. https://doi.org/10.1371/journal.pone.0163962.

Smit, A..FA., Hubley, R. RepeatModeler Open-1.0. 2008-2015 <http://www.repeatmasker.org>. Last accessed 1-Jun-2025.

Smit, A.F.A., Hubley, R., Green, P. RepeatMasker Open-4.0. 2013-2015 <<http://www.repeatmasker.org>> Last accessed 1-Jun-2025.

Stanke, M., Morgenstern, B. 2005. AUGUSTUS: a web server for gene prediction in eukaryotes that allows user-defined constraints. *Nucleic Acids Research* 33: W465-7. https://doi.org/10.1093/nar/gki458.

Tange O. 2018. GNU Parallel 2018, March 2018, https://doi.org/10.5281/zenodo.1146014.

Uliano-Silva, M., Ferreira, J.G.R.N., Krasheninnikova, K., [Darwin Tree of Life Consortium](https://bmcbioinformatics.biomedcentral.com/articles/10.1186/s12859-023-05385-y#group-1), [Formenti](https://bmcbioinformatics.biomedcentral.com/articles/10.1186/s12859-023-05385-y#auth-Giulio-Formenti-Aff4), G., [Abueg](https://bmcbioinformatics.biomedcentral.com/articles/10.1186/s12859-023-05385-y#auth-Linelle-Abueg-Aff4), L., [Torrance](https://bmcbioinformatics.biomedcentral.com/articles/10.1186/s12859-023-05385-y#auth-James-Torrance-Aff1), J., [Myers](https://bmcbioinformatics.biomedcentral.com/articles/10.1186/s12859-023-05385-y#auth-Eugene_W_-Myers-Aff5-Aff6), E.W., [Durbin](https://bmcbioinformatics.biomedcentral.com/articles/10.1186/s12859-023-05385-y#auth-Richard-Durbin-Aff7-Aff1), R., [Blaxter,](https://bmcbioinformatics.biomedcentral.com/articles/10.1186/s12859-023-05385-y#auth-Mark-Blaxter-Aff1) M., McCarthy, S.A. 2023. MitoHiFi: a python pipeline for mitochondrial genome assembly from PacBio high fidelity reads. *BMC Bioinformatics* 24: 288. https://doi.org/10.1186/s12859-023-05385-y.

Wingett, S., Ewels, P., Furlan-Magaril, M., Nagano, T., Schoenfelder, S., Fraser, P., Andrews, S. 2015. HiCUP: pipeline for mapping and processing Hi-C data. F1000Research 4:1310. https://doi.org/10.12688/f1000research.7334.1

Wolff, J., Rabbani, L., Gilsbach, R., Richard, G., Manke, T., Backofen, R., Grüning, B.A. 2020. Galaxy HiCExplorer 3: a web server for reproducible Hi-C, capture Hi-C and single-cell Hi-C data analysis, quality control and visualization. *Nucleic Acids Research* 48:W177–W184. <https://doi.org/10.1093/nar/gkaa220.>

Zhang, Y., Chu, J., Cheng, H., Li, H. 2023. De novo reconstruction of satellite repeat units from sequence data. *Genome Research* 33: 1994-2001. https://doi.org/10.1101/gr.278005.123.

Zhou, C., McCarthy, S.A., Durbin, R. 2023. YaHS: yet another Hi-C scaffolding tool. *Bioinformatics* 39: btac808
