# Supplementary material for: A near telomere-to-telomere phased genome assembly and annotation for the Australian central bearded dragon Pogona vitticeps
Source: Gigascience. 2025 Aug 19;14:giaf085. doi: 10.1093/gigascience/giaf085 (PMC12360841; doi:10.1093/gigascience/giaf085)
Supplement: giaf085_GIGA-D-25-00166_Revision_1 [file giaf085_giga-d-25-00166_revision_1.pdf]

GigaScience

A near-telomere to telomere phased genome assembly and annotation for the Australian central bearded dragon Pogona vitticeps

--Manuscript Draft--

**Manuscript Number:**	GIGA-D-25-00166R1
**Full Title:**	A near-telomere to telomere phased genome assembly and annotation for the Australian central bearded dragon Pogona vitticeps
**Article Type:**	Research

Funding Information:

National Health and Medical Research Council (APP2021172)	Dr. Hardip Patel
Bioplatforms Australia	Professor Arthur Georges
Australian Research Council (DP220101429)	Professor Arthur Georges
Ministerio de Ciencia e Innovación (PID2020-112557GB-I00 funded by AEI/10.13039/501100011033)	Dr Aurora Ruiz-Herrera
Agència de Gestió d'Ajuts Universitaris i de Recerca (2021SGR00122)	Dr Aurora Ruiz-Herrera
Ministerio de Ciencia, Innovación y Universidades (FPU18/03867 and EST22/00661)	Miss Laia Marin-Gual

Abstract:

Background

The central bearded dragon (Pogona vitticeps) is widely distributed in central eastern Australia and adapts readily to captivity. Among other attributes, it is distinctive because it undergoes sex reversal from ZZ genotypic males to phenotypic females at high incubation temperatures. Here, we report an annotated near-telomere to telomere (T2T) phased assembly of the genome of a female ZW central bearded dragon.

Results

Genome assembly length is 1.75 Gbp with a scaffold N50 of 266.2 Mbp, N90 of 28.1 Mbp, 26 gaps and 42.2% GC content. Most (99.6%) of the reference assembly is scaffolded into 6 macrochromosomes and 10 microchromosomes, including the Z and W microchromosomes, corresponding to the karyotype. The genome assembly exceeds standard recommended by the Earth Biogenome Project (6CQ40): 0.003% collapsed sequence, 0.03% false expansions, 99.8% k-mer completeness, 97.9% complete single copy BUSCO genes and an average of 93.5% of transcriptome data mappable back to the genome assembly. The mitochondrial genome (16,731 bp) and the model rDNA repeat unit (length 9.5 Kbp) were assembled. Male vertebrate sex genes Amh and Amhr2 were discovered as copies in the small non-recombining region of the Z chromosome, absent from the W chromosome.
This, coupled with the prior discovery of differential Z and W transcriptional isoform composition arising from pseudoautosomal sex gene Nr5a1, suggests that complex interactions between these genes, their autosomal copies and their resultant transcription factors and intermediaries, determines sex in the bearded dragon.

Conclusion

This high-quality assembly will serve as a resource to enable and accelerate research into the unusual reproductive attributes of this species and for comparative studies across the Agamidae and reptiles more generally.

**Corresponding Author:**	Hardip Patel<br>Australian National University The John Curtin School of Medical Research<br>Acton, ACT AUSTRALIA
**Corresponding Author Secondary**	

Powered by Editorial Manager® and ProduXion Manager® from Aries Systems Corporation

**Information:**	
**Corresponding Author's Institution:**	Australian National University The John Curtin School of Medical Research
**Corresponding Author's Secondary Institution:**	
**First Author:**	Hardip Patel
**First Author Secondary Information:**	
**Order of Authors:**	Hardip Patel
	Kirat Alreja
	Andre L.M. Reis
	J King Chang
	Zahra A. Chew
	Hyungtaek Jung
	Jillian M. Hammond
	Ira W. Deveson
	Aurora Ruiz-Herrera
	Laia Marin-Gual
	Clare E. Holleley
	Xiuwen Zhang
	Nicholas C. Lister
	Sarah Whiteley
	Lei Xiong
	Duminda S.B. Dissanayake
	Paul D. Waters
	Arthur Georges
**Order of Authors Secondary Information:**	
**Response to Reviewers:**	Recommendation: This work represents a major advancement in reptilian genomics. Addressing the above issues—particularly methodological clarity, terminology consistency, and functional validation—will enhance its rigor and impact. Minor revisions are required before acceptance.<br>Reviewer #1<br>The authors de novo assembled a telomere to telomere phased genome assembly of the Australian central bearded dragon Pogona vitticeps, using PacBio HiFi, ONT, HiC, and Illumina sequencing platforms. The assembly achieves remarkable contiguity (scaffold N50: 266.2 Mb) and completeness (97.9% BUSCO score), surpassing Earth Biogenome Project standards. The phased assembly of sex chromosomes (Z/W) and identification of candidate sex-determining genes (Amh, Amhr2, and Nr5a1) provide valuable insights into reptilian sex determination. Overall, the study is well-executed and provides a valuable resource for comparative genomics and reproductive biology.<br>We are grateful for this assessment.<br>Major concern:<br>1.The description of read depth had errors at lines 401-402, such as 60.6x. In addition, “4 x promethION”, “2x150 bp” were should be revised and please check and revise all the similar description in the manuscript.<br>Agreed. We have corrected the values in the text (Lines 412:413) to match the values in Table 1.<br>2.There are errors in the citation format of the journal references, such as the absence of punctuation “.”marks between the title name and the journal name at lines 1005-1009, mixing abbreviations (e.g., “PNAS” vs. “Proceedings of the National Academy of Sciences USA”) (lines 988-990, 1005-1009). Please check carefully the format of all

	references.<br>We have checked the references for consistency and made the necessary changes.<br>3.The script “calculateGC.py and processtrftelo.py” (lines 242 and 245) are mentioned without code availability or parameter details. Provide effective links or repository access.<br>All scripts are available at https://github.com/kango2/ausarg. Please See Line 194-195.<br><br>4.The inconsistent use of "Gb" and "Gbp" is observed; it is recommended to adopt a unified description.<br>Agreed. We have corrected in the manuscript to use Gbp consistently.<br>5.Units were missing in the descriptions in multiple places in Table 1 and 2, such as the unit for "Total Bases" and “Assembly length”; please include them.<br>Agreed. We have corrected this in Table 2. Table 1 did not require any change.<br>6.At lines 683-687, the conclusion that Amh/Amhr2 are sex-determining genes relies solely on positional evidence. Discuss the need for functional studies (e.g., CRISPR knockouts) to strengthen claims.<br>Agreed. We have added text to this effect on lines 716-722 and added additional citations to the reference list:<br>Added Lines 716-722. This will be a fruitful area for future functional studies based on upregulation and knockout technologies (e.g. CRISPR knockouts). Such approaches were necessary to establish the Sry gene on the Y chromosome of eutherian mammals as the master sex determining gene acting through dominance (Sinclair et al., 1990; Koopman et al., 1991), and to establish Drmt1 on the Z chromosomes of birds as the master sex determining gene acting though dosage (Smith et al., 2009; Lambeth et al., 2014).<br>7.There were errors in “Vasimuddin et al. 2019” (line 238) and “Danecek et al. 2021” (line 239). Please check all the other formats of references.<br>Corrected.<br>8.At lines 476-481, BAC mappings are cited as validation but lack visual evidence (e.g., alignment plots in figures or supplements). Please verify the accuracy of Figure 7 at line 478, as it does not correspond with the description.<br>Clarification: Figure 4 includes visual representation of BAC alignments in their physical positions as red diamonds. The reference to Figure 7 reference was incorrect and we have corrected this to refer to Figure 8.<br>Reviewer #2<br>Patel et al. present a genome assembly of the bearded dragon Pogona vitticeps a lizard species that is widely distributed as a pet and known for its interesting sex-determination, which may switch from genetic sex-determination (ZW) to temperature dependent sex-reversal. The methods chosen to assemble the genome are very state-of-the-art including HIFI and ONT long reads, Hi-C and suitable bioinformatic tools.<br>Again, we are grateful for these kind comments.<br>I have to admit that I have recently been reviewing a similar manuscript for Gigascience (https://aus01.safelinks.protection.outlook.com/?url=https%3A%2F%2Fwww.biorxiv.org%2Fcontent%2F10.1101%2F2024.09.05.611321v1&data=05%7C02%7Chardip.patel%40anu.edu.au%7C0b5e4b6669b0461cc50208dda23bb876%7Ce37d725cab5c46249ae5f0533e486437%7C0%7C0%7C638845099894341712%7CUnknown%7CTWFpbGZsb3d8eyJFbXB0eU1hcGkiOnRydWUsIlYiOiIwLjAuMDAwMCIsIlAiOiJXaW4zMiIsIkFOIjoiTWFpbCIsIldUIjoyfQ%3D%3D%7C0%7C%7C%7C&sdata=tzAkvTe%2F8qnoHRmiQweCP7AFtIDhGZoEzX%2FCyhy2Ow0%3D&reserved=0), where a female ZZ P. vitticeps had been sequenced/assembled from long read data of a different nanopore technology and analyses of the ZW-chromosome was done by short read coverage analysis. One of my major comments was that this approach lacked a true assembly of the W-chromosome. Thus, I am happy to see that the assembly of the W-specific region has been achieved here and the sequencing technologies used might even improve the assembly quality over the ZZ assembly in terms of phasing, consensus accuracy etc.<br>The two manuscripts are highly complementary and I think they should be published, if possible, in the very same issue of Gigascience. Surely both groups have invested a lot of efforts. (Reading L. 685, I just have realized that this seems to be the intention of the journal and I very much support this idea.)<br>Yes, that is our intention – to have a simultaneous publication announcing the discovery of AMH on the Z chromosome but absent from the W chromosome. Two labs working independently with the same discovery. It is a good idea to publish

simultaneously in the same journal.
Still there are some minor points that need improvement for the current manuscript:
1. Why do you leave the Z and W splitted into PAR, Z- and W-specific scaffolds and do not assemble the full-length chromosomes (L. 676)? Would the Hi-C data not support that?

The HiC used in the YAHS assembly did not resolve these breaks between the sex chromosome scaffolds 17 and 18 and the pseudo-autosomal region of the Z and W chromosomes. The junctions are obvious, but we decided not to make the joins without supporting evidence. On line 581-582 we have added:
“The Z and W specific sex chromosome scaffolds were identified as 18 and 17, respectively. These represent the non-recombining region of the sex chromosomes. They were not assembled to the PAR in either haplotype despite the use of HiC data for scaffolding”.
Mitochondrial assembly: from ONT only (L. 307), please do a consensus correction with illumina data, or at least show that the MT assembly has a high consensus accuracy (Q40-Q50).
We have now included the assessment of consensus accuracy using the Illumina data as suggested. Please see Lines 313-317 for methods and Lines 686-688 for results. There were no discrepancy observed except for 12 short homopolymer regions (1bp difference), which can be erroneous by either sequencing techniques.
Genome annotation: show BUSCO scores for annotated proteins (do they fit to BUSCO performed on the whole genome?).
Agreed. We have now included BUSCO scores for annotated proteins in the manuscript (Lines 386-387 methods, lines 671-675 of results).
If possible, compare to results of the NCBI RefSeq annotation (is it already available?).
The NCBI RefSeq annotation is not available. They will become available upon the release of the genome assembly post-publication to provide an alternative annotation for those interested.
In this regard please explain the relatively low mapping rates (L. 647) of RNAseq to the annotated sequences.
We have added a sentence to discuss the implication of the low-mapping rates on lines 678-681):
Relatively lower mapping rates of RNAseq data can be attributed to incomplete annotations of untranslated regions and the presence of other non-coding RNAs that are not annotated. Further improvements to annotations in the future will likely improve the utility of genomic resources.

Could you provide some expression data for the Z-specific Amh and AmhR2? Is it differentially expressed in testis/ovary (after correction for copy number)?
We have addressed this on lines 716-722 as outlined above. This work is underway and will be the subject of a future publication. The scope of the question and the work needed to address it is beyond that of a genome resource note. Watch this space.
Table1, could you show results for the two different ONT library types (ligation vs. ultralong kit). It seems the overall yield was low (5 cells -> 100Gb), any speculation why?
The reviewer may have missed this, but Table S4 contain details of individual runs. Lines 396 covers the speculation.
I think assembly statistics (Table2) should also contain contig N50 length as an additional value to show the high continuity of the assembly.
We have added lines 445-447 to describe contig N50, N90 values. Table 2 is not altered because values presented there are applicable to only curated assembly.
L. 488: "48.36 (1 error in 146kb)", I think something is wrong here. Q48.36 would be 1 error in 68.5kb. I would suggest to re-check these values and incorporate them in Table2.
Agreed. Thank you for spotting this error in our calculations. We misinterpreted the error probability value of (0.000014588), which led to the error. The revised number is now presented on line 511. Regarding incorporation of this values in Table 2 is avoided because these values are not calculated for each assembly as they are computationally expensive.
The high consensus accuracy is one selling point compared to the competitor's assembly.
L. 490: "Individual haplotypes were 85.5% complete…". Explain why you are confident that the haplotypes are more complete than the Merqury results suggest (just one

	sentence).<br>We provide justification for this on lines 514-516 where we say:<br>“This shows that assembly completeness metrics for a single haplotype assembly measured using k-mers can be understated for species with high heterozygosity”
**Additional Information:**	
**Question**	**Response**
Are you submitting this manuscript to a special series or article collection?	No
**Experimental design and statistics**<br><br>Full details of the experimental design and statistical methods used should be given in the Methods section, as detailed in our Minimum Standards Reporting Checklist. Information essential to interpreting the data presented should be made available in the figure legends.<br><br>Have you included all the information requested in your manuscript?	Yes
**Resources**<br><br>A description of all resources used, including antibodies, cell lines, animals and software tools, with enough information to allow them to be uniquely identified, should be included in the Methods section. Authors are strongly encouraged to cite Research Resource Identifiers (RRIDs) for antibodies, model organisms and tools, where possible.<br><br>Have you included the information requested as detailed in our Minimum Standards Reporting Checklist?	Yes
**Availability of data and materials**<br><br>All datasets and code on which the conclusions of the paper rely must be either included in your submission or deposited in publicly available repositories (where available and ethically appropriate), referencing such data using	Yes

Powered by Editorial Manager® and ProduXion Manager® from Aries Systems Corporation

a unique identifier in the references and in the “Availability of Data and Materials” section of your manuscript.<br><br>Have you have met the above requirement as detailed in our Minimum Standards Reporting Checklist?	
GigaScience has policies and guidelines in place for the use of generative AI-writing tools such as ChatGPT. If you have used such writing tools to assist with writing the manuscript this must be declared and cited in the text. Authors should not list AI-writing tools and other AI-assisted technologies as an author or co-author and should acknowledge that they are fully responsible for text generated or refined by AI-writing tools.<p><br><br>A summary of use (particularly in the introduction or among methods) needs to be included at the end of the paper, and the outputs should also be included as a supplementary file hosted in GigaDB or other open repositories. Please read our guidelines for more information. <p><br><br>By submitting to GigaScience, you are aware of the journal's AI-writing tools policy, and if you have declared use of such tools below, you have acknowledged this where appropriate in your manuscript and have made a summary of use and outputs available. <p><br><b>AI-assisted writing tools have been used in the preparation of this manuscript?	No

Manuscript
Click here to access/download;Manuscript;Pogona_genome_05-06-2025_nea

A near telomere-to-telomere phased genome assembly and annotation for the Australian central bearded dragon *Pogona vitticeps*

Hardip R. Patel[1*], Kirat Alreja[1], Andre L.M. Reis[2,3,4], J King Chang[8], Zahra A. Chew[1], Hyungtaek Jung[1], Jillian M. Hammond[2,3], Ira W. Deveson[2,3,4], Aurora Ruiz-Herrera[5,6], Laia Marin-Gual[5,6], Clare E. Holleley[7], Xiuwen Zhang[9], Nicholas C. Lister[8], Sarah Whiteley[9], Lei Xiong[9,10], Duminda S.B. Dissanayake[9], Paul D. Waters[8], Arthur Georges[9*]

[1] National Centre for Indigenous Genomics, John Curtin School of Medical Research, Australian National University, Canberra, ACT 2601, Australia

[2] Genomics and Inherited Disease Program, Garvan Institute of Medical Research, Sydney, New South Wales, Australia

[3] Centre for Population Genomics, Garvan Institute of Medical Research and Murdoch Children's Research Institute, Darlinghurst, New South Wales, Australia

[4] Faculty of Medicine, University of New South Wales, Sydney, New South Wales, Australia

[5] Department of Cellular Biology, Physiology and Immunology, Universitat Autònoma de Barcelona (UAB), Cerdanyola del Vallès, 08193, Spain.

[6] Genome Integrity and Instability Group, Institut de Biotecnologia i Biomedicina, Universitat Autònoma de Barcelona (UAB), Cerdanyola del Vallès, 08193, Spain.

[7] Australian National Wildlife Collection, CSIRO, Canberra ACT, Australia

[8] Faculty of Science, School of Biotechnology, and Biomolecular Science, UNSW Sydney, Sydney, NSW, Australia

[9] Institute for Applied Ecology, University of Canberra ACT 2601, Australia

[10] Wannan Medical College, Whu, Anhui, 241001 China

*Correspondence: Hardip Patel, hardip.patel@anu.edu.au; Arthur Georges, arthur.georges@canberra.edu.au

OrcidID

Patel – http://orcid.org/0000-0003-3169-049X

Alreja - https://orcid.org/0009-0007-8937-9844

Martin-Reis https://orcid.org/0000-0002-7300-1157

Chang – https://orcid.org/0009-0007-8748-4368

Chew - https://orcid.org/0009-0006-3385-1743

Deveson https://orcid.org/0000-0003-3861-0472

Ruiz-Herrera - https://orcid.org/0000-0003-3868-6151

Marin - https://orcid.org/0000-0003-1480-0976

Jung - https://orcid.org/0000-0003-2464-1235

Holleley - https://orcid.org/0000-0002-5257-0019

Zhang - https://orcid.org/0000-0001-9186-9892

Lister - http://orcid.org/0000-0002-6597-4784

Whiteley - https://orcid.org/0000-0003-3372-4366

Xiong - https://orcid.org/0000-0002-6076-4438

Dissanayake – https://orcid.org/0000-0002-7307-4639

Waters – http://orcid.org/0000-0002-4689-8747

Georges – http://orcid.org/0000-0003-2428-0361

Abstract

Background The central bearded dragon (*Pogona vitticeps)* is widely distributed in central eastern Australia and adapts readily to captivity. Among other attributes, it is distinctive because it undergoes sex reversal from ZZ genotypic males to phenotypic females at high incubation temperatures. Here, we report an annotated near telomere-to-telomere phased assembly of the genome of a female ZW central bearded dragon.

Results Genome assembly length is 1.75 Gbp with a scaffold N50 of 266.2 Mbp, N90 of 28.1 Mbp, 26 gaps and 42.2% GC content. Most (99.6%) of the reference assembly is scaffolded into 6 macrochromosomes and 10 microchromosomes, including the Z and W microchromosomes, corresponding to the karyotype. The genome assembly exceeds standard recommended by the Earth Biogenome Project (6CQ40): 0.003% collapsed sequence, 0.03% false expansions, 99.8% k-mer completeness, 97.9% complete single copy BUSCO genes and an average of 93.5% of transcriptome data mappable back to the genome assembly. The mitochondrial genome (16,731 bp) and the model rDNA repeat unit (length 9.5 Kbp) were assembled. Male vertebrate sex genes *Amh* and *Amhr2* were discovered as copies in the small non-recombining region of the Z chromosome, absent from the W chromosome.

This, coupled with the prior discovery of differential Z and W transcriptional isoform composition arising from pseudoautosomal sex gene *Nr5a1*, suggests that complex interactions between these genes, their autosomal copies and their resultant transcription factors and intermediaries, determines sex in the bearded dragon.

Conclusion This high-quality assembly will serve as a resource to enable and accelerate research into the unusual reproductive attributes of this species and for comparative studies across the Agamidae and reptiles more generally.

Keywords: Squamata; Agamidae; lizard; AusARG; sex determination

Species Taxonomy

Eukaryota; Animalia; Chordata; Reptilia; Squamata; Iguania; Agamidae; Amphibolurinae; *Pogona*; *Pogona vitticeps* (Ahl, 1926) (NCBI:txid103695).

Graphical Abstract

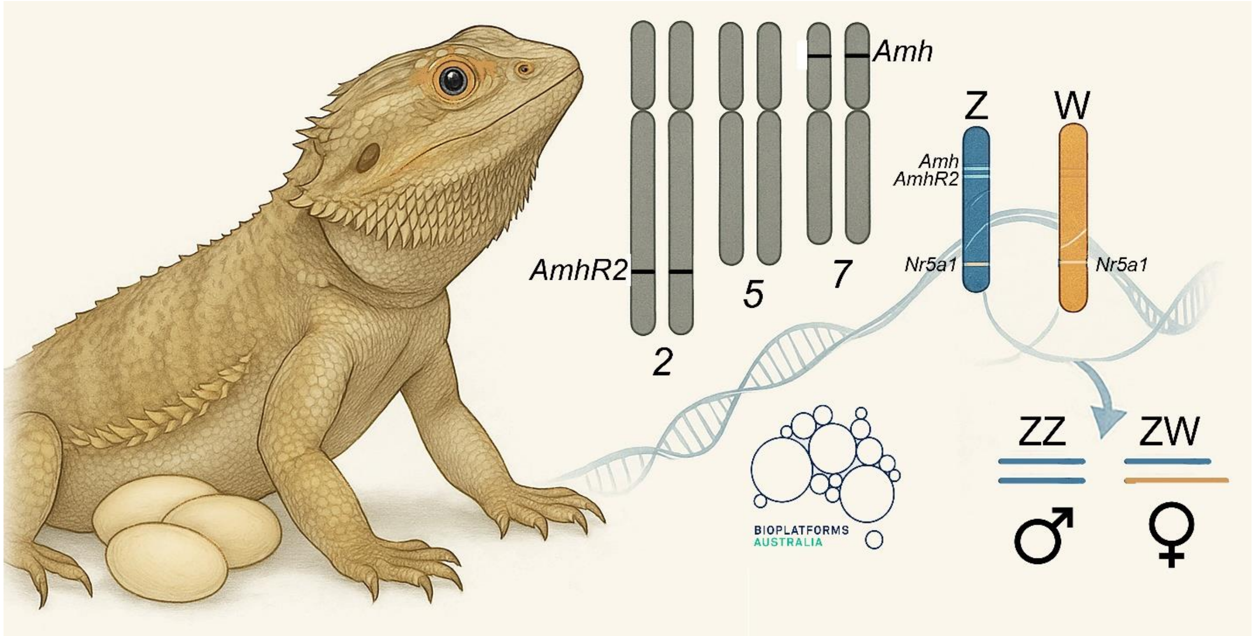

Introduction

The family Agamidae, commonly known as dragon lizards, is a diverse group of lizards found in Africa, Asia, Australia, the Western Pacific, and warmer regions of Southern Europe. The Agamidae family is well represented in Australia, in part because of their successful radiation in response to the progressive aridification of the Australian continent during the Pleistocene. New species are continually being described, but on recent count they comprise 81 species in 15 genera (Cogger 2018) that occupy a very wide array of habitats ranging from the inland deserts to the mesic habitats of the coast and the Australian Alps below the tree-line. The family includes some iconic species such as the thorny devil *Moloch horridus* and the frillneck lizard *Chlamydosaurus kingii*. Less spectacular perhaps is the central bearded dragon *Pogona vitticeps* (Ahl, 1926), a widely distributed species of Amphibolurine dragon common in central eastern Australia (Figure 1). The bearded dragon feeds on insects and other invertebrates, but a substantial component of the diet of adults is vegetable matter. It lives in the dry sclerophyll forests and woodlands in the southeast of its range, mallee and arid acacia scrublands further north and west, and the sandy deserts of the interior. Semi-arboreal, the species often perches on fallen timber and tree branches only to retreat to ground cover when disturbed.

Central bearded dragons adapt readily to captivity, lay large clutches of eggs several times per season, and are commonly kept as a pet in Europe, Asia, and North America. These attributes also increase its value as a popular reptile research model in a range of disciplines (Ollonen et al., 2018; Bonnan et al., 2024; Chandrasekara et al., 2024; Fenk et al., 2024; Nagashima et al., 2024; Razmadze et al., 2024). Central bearded dragons are a particularly compelling model species for sex determination because they display temperature-induced sex reversal in the laboratory and in the wild (Quinn et al., 2007; Holleley et al., 2015; Castelli et al., 2021). The sex chromosomes of central bearded dragons are poorly differentiated morphologically. They exhibit female heterogamety (ZZ/ZW sex chromosome system, Ezaz et al., 2005) with 6 macrochromosome pairs and 10 microchromosome pairs (Witten, 1983) that includes the sex microchromosome pair (Ezaz et al., 2005). BAC sequences have been physically mapped uniquely to each of the chromosomes (Young et al., 2013; Deakin et al., 2016).

Sex determination in this species is particularly subtle until now with no substantial difference between the Z and W chromosome gene content or single-copy sequence (Zhang et al., 2022). The developmental program initiated by chromosomal sex determination can be reversed by high incubation temperature, allowing for investigations of environmental influences on fundamental developmental processes. Research in these areas of interest will be greatly facilitated by applying modern sequencing technologies to generate a high-quality draft genome assembly for the central bearded dragon. The ability to generate telomere-to-telomere (T2T) assemblies of the sex chromosomes and identify the non-recombining regions within which lies any master sex determining gene will greatly narrow the field of candidate sex determining genes in species with chromosomal sex determination. Furthermore, the disaggregation of the Z and W sex chromosome haplotypes (phasing) will allow comparisons of the Z and W sequences to gauge putative loss or difference in function of key sex gene candidates.

In this paper, we present a draft annotated near-T2T phased assembly of the genome of the Australian central bearded dragon as a resource to enable and accelerate research into the unusual reproductive attributes of this species and for comparative studies across the Agamidae and reptiles more generally. This is a vastly improved assembly in comparison with an earlier assembly based on Illumina short-read technology published in 2015 (Georges et al., 2015).

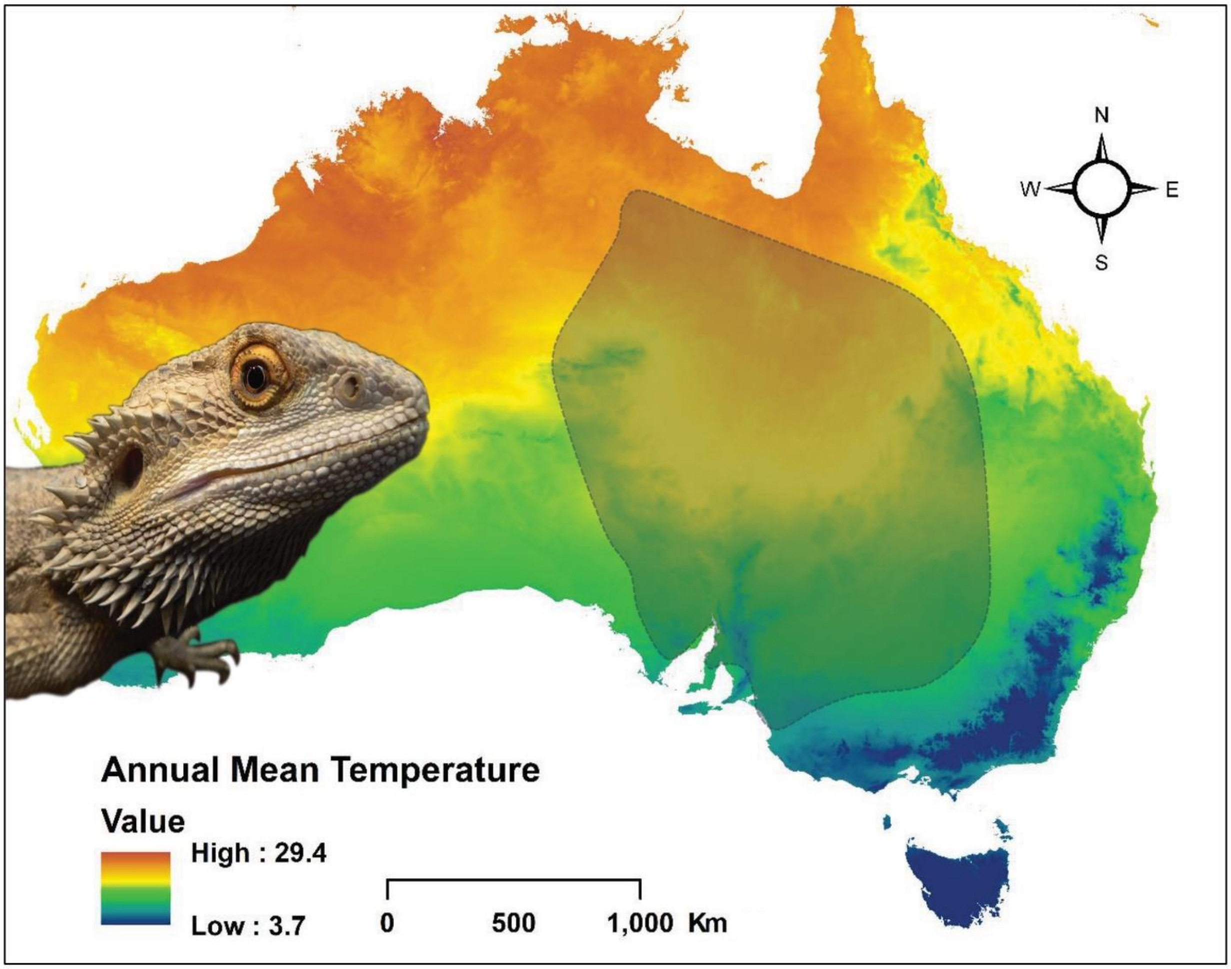

Figure 1. The central bearded dragon *Pogona vitticeps* and the distribution of the species based on records from Australian museums (via Atlas of Living Australia https://www.ala.org.au/).

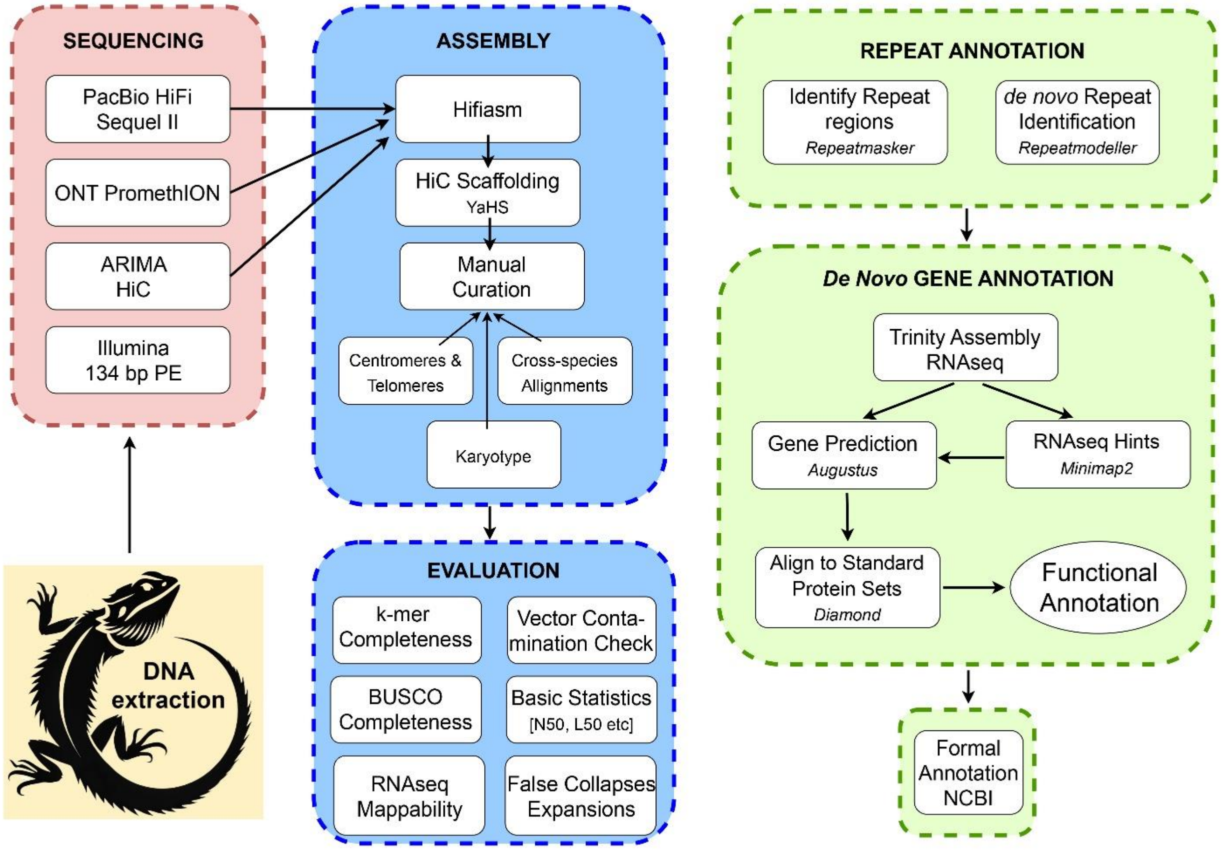

Figure 2. Schematic overview of workflow for sequencing, assembly and annotation of the genome of the central bearded dragon *Pogona vitticeps*. Target: Earth Biogenomes Project standard 6CQ40 (Lawniczak et al., 2022). Illumina 134 bp PE reads (Table S6) were not used directly in the assembly, but for quality assessment of the genome. Quality control workflow not shown. Repeat annotation was undertaken with Repeatmasker (4.1.2-p1, Smit et al., 2013-2015). Refer to Table S1 for software used in this project.

Materials and Methods

Sample collection

DNA samples were obtained from a blood sample taken from a single female *Pogona vitticeps* (RadMum, UCID Pit_001003342236, NCBI:txid103695) collected on 15-Mar-2011 on a road verge 62 km west of Eulo on Adventure Way, Queensland (GPS -28.099000 144.433000). It was verified as a ZW female using sex-linked polymerase chain reaction (PCR) markers (Holleley et al., 2015).

An additional 3 adult individuals were sampled to provide tissues (brain, heart, kidney, liver, lung, skeletal muscle, testes, ovary), complemented by embryonic brain and gonad, for transcriptomics (Table S2).

Extraction and Sequencing

We generated sequencing data using three platforms – PacBio HiFi, ONT ultralong reads and HiC generated using the Arima Genomics protocols (Figure 2). Illumina short read DNA data were previously generated (Georges et al., 2015). Transcriptome data were generated using the Illumina platform. All sequence data generated in this study are available from NCBI SRA under BioProject ID PRJNA1252275.

PacBio HiFi: Genomic DNA was extracted from blood of the focal ZW individual by PacBio Asia (Singapore) and sequenced using two flow cells on a PacBio Sequel II (Table S3). HiFi data were processed using *cutadapt* (v3.7, parameters: --anywhere --error-rate 0.1 --overlap 25 --match-read-wildcards --revcomp --discard-trimmed) to remove reads containing PacBio primers and adaptor sequences. This step removes putative chimeric sequences.

ONT PromethION: Genomic DNA was extracted from blood of the focal ZW individual (Table S4) using the salting out procedure (Miller et al., 1988) and spooled to enrich for high molecular weight DNA. DNA was shipped to the Garvan Institute of Medical Research in Sydney. Library preparation was performed with 3 µg of DNA as input, using the SQK-LSK109 kit (Oxford Nanopore Technologies, UK) and sequenced across 4 x promethION (FLO-PRO002) flow cells, with washes (EXP-WSH004) performed when sequencing dropped.

A second extraction was performed on 10 µl of blood using the Circulomics UHMW extraction kit, following the “Nucleated blood” protocol, obtaining approximately 60 µg of ultra-high molecular weight DNA. Library preparation was then performed using a pre-release version of the SQK-ULK001 kit from Oxford Nanopore Technologies, which uses the RAP adapter. The library was then loaded onto one promethION (FLO-PRO002) flow cell with washes (EXP-WSH004) performed at 24 and 48 hours to increase output.

ONT basecalling was performed using the *buttery-eel* (v0.4.2+dorado7.2.13, parameters: --config dna_r9.4.1_450bps_hac_prom.cfg --detect_mid_strand_adapter --trim_adapters --detect_adapter --do_read_splitting --qscore 7). Parameters were chosen to remove reads with

average quality value score <7, remove adapters at 5’ or 3’ ends of sequence, and to split reads if adapters were in the middle of the read.

HiC: A blood sample from the focal ZW individual (Table S5) was used for HiC. Blood sample was processed by the Biomolecular Resource Facility (BRF) at the Australian National University using the Arima HiC 2.0 kit for library preparation and sequencing with two flow cells on an Illumina NovaSeq 6000 NovaSeq 6000, S1 300 cycles kit 2x150 bp.

RNA: Total RNA was extracted from adult brain, liver, heart and ovary/testis (Table S2) by the Garvan Institute of Medical Research (Sydney). Tissue extracts were homogenized using T10 Basic ULTRA-TURRAX® Homogenizer (IKA, Staufen im Breisgau, Germany) and extracted using TRIzol reagent following the manufacturer’s instructions, purifying with an isopropanol precipitation. Seventy-five bp single-end reads were generated for recent samples on the Illumina NextSeq 500 platform at the Ramaciotti Centre for Genomics (UNSW, Sydney, Australia). Some earlier samples generated 100 bp PE reads.

RNAseq from three embryonic gonads were sourced from Whiteley et al. (2022) and RNAseq from three embyronic brains were sourced from Whiteley et al. (2021) and Wagner et al. (2023) (Table S2).

Assembly

All data analyses were performed on the high-performance computing facility, Gadi, hosted by Australia’s National Computational Infrastructure (NCI, https://nci.org.au). Scripts are available at https://github.com/kango2/ausarg.

Primary genome assembly: PacBio HiFi, ONT and HiC sequence data were used to generate interim haplotype assemblies and an interim pseudohaplotype (=consensus haplotype) assembly using *hifiasm* (v0.19.8, Cheng et al., 2021, 2022, RRID:SCR_021069, default parameters). HiC data were aligned to the interim pseudohaplotype and haplotype assembly using the *Arima Genomics alignment pipeline* (Arima Genomics, 2024) following the user guide for scaffolding and assessing the accuracy of assembly. HiC read alignments were processed using *YaHS* (v1.1, Zhou et al., 2022, RRID:SCR_022965 , parameters: -r 10000, 20000, 50000, 100000, 200000, 500000, 1000000, 1500000 --no-contig-ec -e GATC,GANTC,CTNAG,TTAA) to generate scaffolds. Range resolution parameter (-r) in *YaHS* was restricted to 1500000 to ensure

separation of microchromosomes into individual scaffolds. Contig correction was disabled to maintain the original contig structure produced by *hifiasm*.

HiC contact maps were processed and visualised using *Juicer (*v1.5, Durand et al., 2016, RRID:SCR_017226). Read depth, GC content, and telomere locations for *YaHS* scaffolds >1 Mbp length were visually inspected. One scaffold in the pseudohaplotype assembly contained internal telomeric repeat and contact pattern of a mis-join, owing to incorrect contig assembly by *hifiasm*. Similar error was observed for one scaffold in haplotype 2 as a result of scaffolding error (Figure S2). YaHS was rerun without --no-contig-ec parameter, which is the default behaviour that fixed these errors.

Reference genome assembly: The karyotype was obtained from Witten (1983) and Ezaz et al. (2005) as a guide for the expected number of chromosomes for final T2T assembly. A reference assembly was *generated* by choosing the best chromosome scaffolds from one of the two haplotype assemblies. The basis for selection was as follows for scaffolds >1Mbp in size. If the scaffold of haplotype 1 had both ends represented by telomeric sequence and the corresponding scaffold of haplotype 2 had only one end represented by telomeric sequence, then the scaffold for haplotype 1 was chosen for the reference assembly, and vice versa. If both the scaffolds for haplotype 1 and haplotype 2 contained telomeric sequence at both ends, then the scaffold with the fewest gaps was chosen for the reference assembly. If both T2T haplotypes had the same number of gaps, then the longest scaffold was selected for the reference assembly. If both haplotypes were equal in telomere presence, number of gaps and length, haplotype 1 sequence was chosen for the reference assembly. The Z and W specific scaffolds were added to the reference assembly. All scaffolds <1Mbp were drawn from haplotype 1 for the reference assembly.

Chromosome assignments: Bacterial Artificial Chromosome (BAC) clones were previously used for generating physical map for *Pogona vitticeps* (Young et al., 2013; Deakin et al., 2016). BAC end sequences (n=273) corresponding to 137 clones were downloaded from the NCBI GSS database. These sequences were aligned to the reference genome using *minimap2* (parameters: -x asm20 --secondary-no) to identify their locations in the reference genome. We also mapped the sex-linked sequence represented by 3,288 bp Clone C1 of Quinn et al. (2010) (Genbank accession EU938138) generated by walking out from a sex-linked 50 bp AFLP Pvi72W marker

(Genbank accession ED982907) identified by Quinn et al. (2007) to confirm the assignment of a scaffold to the non-recombining region of the W chromosome (Scaffold 17).

Read depth and GC content calculations: PacBio HiFi (parameter: -x map-pb) and ONT (parameter: -x map-ont) sequence data were aligned to the scaffold assembly using *minimap2* (v2.17, Li 2018) Similarly, Illumina sequence data were aligned to the assembly using *bwa-mem2* (v2.2.1, Vasimuddin et al., 2019) using default parameters. Resulting alignment files were sorted and indexed for efficient access using *samtools* (v1.19, Danecek *et al.*, 2021). Read depth in non-overlapping sliding windows of 10 Kbp was calculated using the *samtools bedcov* command. GC content in non-overlapping sliding windows of 10 Kbp was calculated using *calculateGC.py* script.

Telomere repeats: Tandem Repeat Finder (*TRF*) (v4.09.1, Benson 1999, parameters: 2 7 7 80 10 500 6 -l 10 -d -h) was used to detect all repeats up to 6 bp length. TRF output was processed using *processtrftelo.py* script to identify regions >600 bp that contained conserved vertebrate telomeric repeat motif (TTAGGG). These regions were labeled as potential telomeres.

Centromere annotations: Enrichment of satellite repeats, increased inter-chromosomal HiC contacts (Mokhtaridoost et al., 2024), and reduced recombination typically mark centromeric regions. To identify satellite repeats we followed the procedure described by Zhang et al. (2023) with some modifications. Briefly, we counted 101-mers occurring 20 times or more with k-mer counter *KMC* (v3.2.4, Kokot et al., 2017, parameters: k=101, ci=20, -cs=100000). Satellite Repeat Finder (*SRF*, Zhang et al., 2023, commit id e54ca8c) was used to identify putative satellite repeats using those k-mers. Identified repeat units were elongated up to 1000 bp if they were <1000 bp, and all-vs-all alignments were performed using *minimap2* to group repeats into classes based on their sequence similarity. The reference genome was aligned to the identified repeat units using *minimap2* (Li, 2018 Parameters: –c –N1000000 –f1000 –r100,100 <(srfutils.js enlong srf.fa)). Note that repeat units <200 bp were extended to 200 bp before alignments using the srfutils.js utility in *SRF*. Alignments were processed using *srfprocess.R* script to merge consecutive alignments to the same repeat unit separated by <10 bp. All regions >100 bp long and 10% of the repeat unit length were retained for further analysis. If a genomic region overlapped multiple repeat classes, the longer region with its repeat class was chosen as a set of putative satellite repeat region with corresponding repeat class.

HiC inter-chromosomal interactions were examined and quantified for their association with centromeres. HiC data were mapped against the reference genome using the *GEM mapper* (v3.6.1, Marco-Sola et al., 2012) from *TADbit* (v1.0.1, Serra et al., 2017). Reads were iteratively mapped using windows from 15 bp to 75 bp in 5 bp steps. Possible artifacts were then removed, including: "self-circle", "dangling-end", "error", "extra dangling-end", "too short", "too large", "duplicated" and "random breaks". Binning and data normalization were conducted using an in-house script that imports the "HiC_data" module of *TADbit* to bin unique reads into a square matrix of 50 Kbp. A 500 Kbp matrix was created and subsequently processed with *HiCExplorer* (v3.7, Ramírez et al., 2018, RRID:SCR_022111). Both 50 Kbp and 500 Kbp matrices were corrected with Iterative Correction and Eigenvector (ICE) decomposition and normalized to a total of 100,000,000 interaction counts by scaling the sum of all interactions within the matrix. Normalized matrices were then plotted at a 500 Kbp resolution using *HiCExplorer*. The normalized 50 Kbp matrix was transformed into a GInteraction table using *HiCExplore*r, which includes interaction values between all genomic bins. Inter-chromosomal interactions were log-transformed and normalized to obtain Z-score values for each chromosome and genomic bin, as previously described (Alvarez-Gonzalez et al., 2022; Bista et al., 2024). Z-score values were plotted with ggplot2 as points and the LOESS method (span=0.4, Cleveland, 1979) was used for best fit line.

For measuring heterozygosity changes across the genome, each haplotype sequence was aligned to the reference genome using *minimap2* (parameters: -x asm5 --cs –K 1000M). Resulting alignments were processed using *paftools.js call* to identify variant sites. Since one of the haplotype sequences is the reference sequence, all variable sites are considered as heterozygous sites. Heterozygous variant site counts in 50 Kbp windows were counted and plotted using *ggplot2*. LOESS smoothing (span=0.5) was applied for the best fit line.

Sex chromosome identification: The putative Z and W scaffolds will have half the read depth of the autosomal scaffolds in a ZW individual. Scaffolds >1 Mbp long were examined for median read depths in 10 Kbp windows. Sex specific Z and W scaffolds were identified by having approximately half the median read depth of autosomes and the PAR in the sequenced ZW individual (Figure 10). The PAR scaffold was identified by homology with known Z chromosome sequence.

HiC analysis for sex chromosome differences in contact maps: HiC reads were quality-trimmed using *Trimmomatic* v0.39 (Bolger et al., 2014)(RRID:SCR_011848) to remove adapter sequences and low-quality reads. The trimmed reads of HiC data and Illumina DNA sequence data were aligned to both genome haplotypes using *BWA-mem* (v0.7.17). PacBio data were aligned to both genome haplotypes using *minimap2* v2.28. Resulting BAM files were merged and coordinate-sorted using *SAMtools* v1.19.2 (RRID:SCR_002105). Variant calling for each haplotype was performed using Illumina BAM files with *FreeBayes* v1.3.8 to generate VCF files. These VCF files were normalized using *BCFtools* v1.14, then compressed using *bgzip* from *HTSlib* v1.20. Phasing of VCF files was then conducted using *WhatsHap* v2.3 to resolve haplotype-specific information across the dataset, using genome haplotypes, normalized VCF files and PacBio BAM files as inputs. Phased VCF files were then used to phase the mapped HiC reads.

Mitochondria genome assembly: PacBio HiFi and ONT sequences were aligned to a *Pogona vitticeps* reference (NCBI Accession: NC_006922, Amer and Kumasawa, 2005) using *minimap2* (RRID:SCR_018550, parameters: --map-pb or --map-ont) to search for mitochondrial reads. Alignments were processed to identify reads <20 Kbp and aligned residues >5 Kbp. No PacBio HiFi reads were identified using this filter. ONT reads were assembled using *flye* (v2.9.3, parameters: -- iterations 2, Kolmogorov et al., 2019) to generate mitochondrial genome sequence. The output assembly sequence was processed using *MitoHiFi* (v2.9.5, Uliano-Silva et al., 2023, RRID:SCR_026369) to adjust the start coordinate and obtain annotations. Illumina data were aligned to the reference genome including mitochondrial sequence using *bwa-mem2* (v2.2.1, parameters: -Y -K 100000000). Alignments were processed using *bcftools* (v1.21, parameters: mpileup --min-MQ 30 -r --adjust-MQ --redo-BAQ --fasta-ref reference.fasta aligned.bam | bcftools call -mv -Ov --ploidy 1 >out.vcf) to call variants on the mitochondria sequence.

Assembly evaluation

The assembly was evaluated against criteria established by the Earth Biogenomes Project (EBP, https://www.earthbiogenome.org/report-on-assembly-standards, version 6CQ40, Lawniczak et al., 2022) namely: percentage of collapsed sequence, percentage false expansions, k-mer

completeness, complete single copy BUSCO genes, and average percentage of transcriptome data mappable to the genome assembly and contaminations (Figure 2).

K-mer completeness and per base error rate estimation: Illumina sequence data were trimmed for adapters and low-quality reads using *Trimmomatic* (v0.39, Bolger et al., 2014, parameters: ILLUMINACLIP:TruSeq3-PE.fa":2:30:10:2:True LEADING:3 TRAILING:3 SLIDINGWINDOW:4:20 MINLEN:36). Resultant paired-end sequences were used to generate k-mer database using *meryl* (v1.4.1, Rhie et al., 2020). *Merqury* (v1.3, Rhie et al., 2020) was used with *meryl* k-mer database to evaluate assembly k-mer completeness and estimate per base error rate of pseudo-haplotype and individual haplotype assemblies.

False expansions and collapses: Putative false expansion and collapse metrics were calculated using the *Inspector* (v1.2, Chen et al., 2021, default parameters) and PacBio HiFi data.

Contamination check: Vector contamination was assessed using *VecScreen* defined parameters for *BLAST* (v2.14.1, Camacho et al., 2009, parameters: -task blastn -reward 1 -penalty -5 -gapopen 3 -gapextend 3 -dust yes -soft_masking true -evalue 700 -searchsp 1750000000000) and the *UniVec* database (accessed on 18th June 2024).

Gene completeness evaluation: BUSCO (v5.4.7, Manni et al., 2021, RRID:SCR_015008) was run using *sauropsida_odb10* library in offline mode to assess completeness metrics for conserved genes. BUSCO synteny plots were created with *ChromSyn* (v1.3.0, Edwards et al., 2022).

RNAseq mapping rate: RNAseq data from multiple tissues (Table S2) were aligned to the assembly using *subread-align* (v2.0.6, parameters: -n 150 Liao et al., 2013) to calculate percentage of mapped fragments for evaluating RNAseq mapping rate. We chose –n 150 to sample all possible seeds for alignments because of high heterozygosity observed for the species. We did not have RNAseq data for the focal individual used for the genome assembly.

Annotation

Repeat annotation: RepeatModeler (v2.0.4, Smit et al. (2008-2015), RRID:SCR_015027, parameters: -engine ncbi) was used to identify and classify repetitive DNA elements in the genome. Subsequently, *RepeatMasker* (v4.1.2-pl, Smit et al. (2013-2015), RRID:SCR_012954)

was used to annotate and soft-mask the genome assembly using the species-specific repeats library generated by *RepeatModeler* and families were labelled accordingly.

Ribosomal DNA: Assembled scaffolds were searched for ribosomal DNA units using *ribocop.py* which searches for consecutive alignments of 18S, 5.8S, and 28S to determine rDNA sequences.

De novo gene annotations: RNAseq data from multiple tissues (Table S2) were processed using *Trinity* (v2.12.0, Grabherr et al., 2011, parameters: --min_kmer_cov 3 --trimmomatic) to produce individual transcriptome assemblies. Parameters were chosen to remove low abundance and sequencing error k-mers. The assembled transcripts were aligned to the UniProt-SwissProt database (last accessed on 28-Feb-2024) using *diamond* (v2.1.9, Buchfink et al., 2021, parameters: blastx --max-target-seqs 1 --iterate --min-orf 30). Alignments were processed using *blastxtranslation.pl* script to obtain putative open reading frames and corresponding amino acid sequences. Transcripts containing both the start and the stop codons, with translated sequence length between 95% and 105% of the best hit to UniProt_SwissProt sequence, were selected as full-length transcripts.

Amino acid sequences of full-length transcripts were processed using *CD-HIT* (v4.8.1, Fu et al., 2012, parameters: -c 0.8 -aS 0.9 -g 1 -d 0 -n 3) to cluster similar sequences with 80% pairwise identity and where the shorter sequence of the pair aligned at least 90% of its length to the larger sequence. A representative transcript from each cluster was aligned to the repeat-masked genome using *minimap2* (v2.26, parameters: --splice:hq), and alignments were coordinate-sorted using *samtools*. Transcript alignments were converted to *gff3* format using *AGAT* (v1.4.0, Dainat, 2022, agat_convert_minimap2_bam2gff.pl) and parsed with *genometools* (v1.6.2, Gremme et al., 2013) to generate training gene models and hints for *Augustus* (v3.4.0, Stanke et al., 2008) with untranslated regions (UTRs). Similarly, transcripts containing both start and stop codons with translated sequence length outside of 95% and 105% of the best hit to UniProt_SwissProt sequence, were processed in the same way to generate additional hints. A total of 500 of these representative full-length transcripts were used in training for gene prediction to calculate species-specific parameters. During the gene prediction model training, parameters were optimized using all 500 training gene models with a subset of 200 used only for intermediate evaluations to improve run time efficiency. Gene prediction for the full dataset used 20 Mbp chunks with 2 Mbp overlaps to improve run time efficiency.

An issue was identified where the predicted *Amh* gene on the Z-specific scaffold (scaffold 18) was fused with neighboring genes. To resolve this, gene prediction was rerun on the Z scaffold with manually modified hints. Specifically, the weighting of UTR hints intersecting with the two predicted introns flanking the *Amh* coding sequence was increased to down weight intronic predictions by *Augustus* in that region. The updated Z scaffold gene predictions were then concatenated with the original gene predictions.

Completeness of annotated genes was evaluated using *BUSCO* (v5.8.2, parameters: --mode protein) in offline mode using sauropsida_odb10 library. Predicted genes were aligned against Uniprot_Swissprot database for functional annotation using best-hit approach and *diamond*. Unaligned genes were subsequently aligned against Uniprot_TrEMBL database for functional annotation (RRID:SCR_002380).

Results and Discussion

DNA sequence data quantity and quality

PacBio HiFi sequencing yielded 70.6 Gbp with a mean read length of 14,980 bp (Table 1, Table 3) and mean quality value >Q30 of all reads. The ONT sequencing yielded 105.6 Gbp of reads with an N50 value of ~37 Kbp and 48.1% reads with mean quality value >Q20 (Table 1, Table S4). HMW DNA often results in lower yields for the ONT platform. The distributions of quality scores and read lengths for the long-read sequencing align with known characteristics of the ONT and PacBio platforms (Figure S1). K-mer frequency histograms of Illumina, ONT and PacBio HiFi sequence data for k=17, k=21 and k=25 show two distinct peaks (Figure 3) confirming the diploid status of this species. The peak for heterozygous k-mers was smaller for k=17 compared to the homozygous k-mer peak. In contrast, the heterozygous k-mer peak was higher for k=25 compared to the homozygous k-mer peak, suggestive of high heterozygosity at a small genomic distance. Genome size was estimated to be 1.81 Gbp using the formulae of Georges et al. (2015) and Illumina sequence data, with a k-mer length of 17 bp, homozygous peak of 45.5 (Figure 3) and the mean read length of 134.3 bp. However, the PacBio estimate of genome size of 1.74 Gbp agrees more closely with the previous estimate using earlier Illumina reads (Georges et al., 2015) and the estimate from flow cell cytometry of 1.77 Gbp (Georges et al., 2015). The reason for the discrepancy between the current and former estimates of genome

size from Illumina data is unclear, but may have arisen because the current Illumina data was not filtered for error reads in the same way.

Read depth, obtained by dividing the total DNA sequence data from each platform by the assembly size, was consistent (Table 1) with the median read depths of 60.5*x* for ONT, 40.4*x* PacBio HiFi and 52.6*x* Illumina platforms calculated for 10 Kbp non-overlapping sliding windows of the assembly.

Table 1. Summary metrics for sequence data and assembly for the bearded dragon *Pogona vitticeps*.

Sequencing Platform	Number of Reads	Mean Read Length (bp)	Total Bases	Est. Genome size (Gbp)	Read Depth
Illumina PE DNA	694,401,150	134	93,255,202,850	1.81	52.6*x*
PacBio HiFi Sequel II	4,714,654	14,980	70,625,888,904	1.74	40.4*x*
ONT R9.4.1	7,118,515	14,830	105,566,275,033	2.7	60.5*x*
Arima Genomics HiC	590,697,330	151	89,195,296,830	--	--

Table 2. Summary metrics for the genome assembly of the bearded dragon *Pogona vitticeps*. The pseudo-haplotype is a combination of haplotypes 1 and 2 (sensu *hifiasm*); the Reference Assembly was constructed by selecting the best scaffolds from each of haplotypes 1 and 2.

Metric	Haplotype 1	Haplotype 2	Pseudo-haplotype	Reference Assembly
Assembly length (bp)	1,752,200,003	1,747,167,247	1,747,460,405	1,752,814,424
No. of scaffolds/contigs	89	51	71	89
GC Content (%)	42.2	42.2	42.2	42.2
No. of gaps > 100 bp	31	28	15	26
Mean sequence length (bp)	19,687,640	34,258,181	24,612,118	19,694,544
Median sequence length (bp)	77,000	152,108	85,021	77,000
Longest sequence (bp)	359,918,989	358,276,425	359,349,958	358,276,425
Shortest sequence (bp)	4,000	18,330	4,000	4,000
N50 (bp)	265,980,915	266,210,064	266,029,613	266,210,064
N90 (bp)	28,115,431	28,121,876	28,118,385	28,115,431
L50	3	3	3	3
L90	9	9	9	9

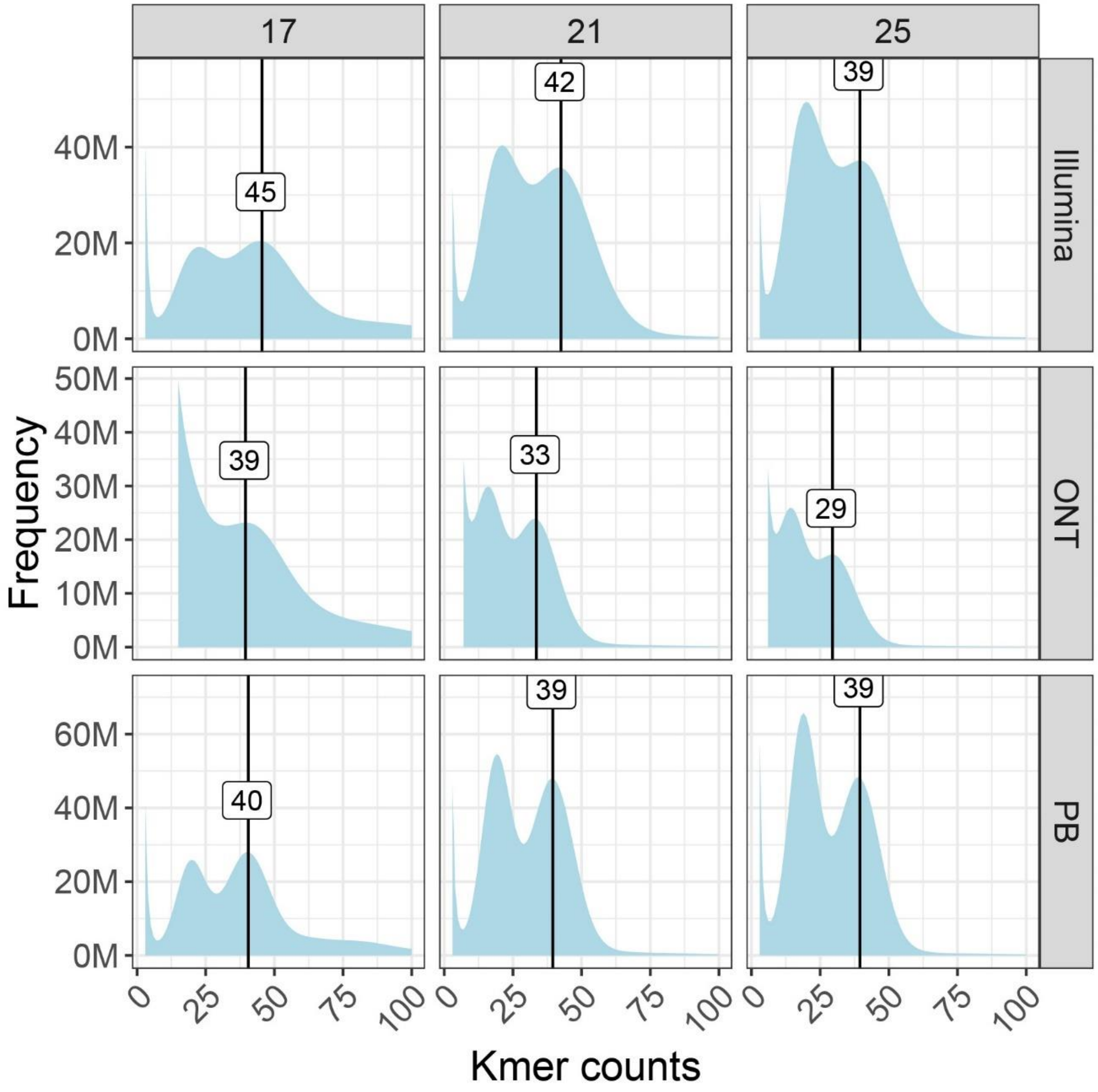

Figure 3. Distribution of k-mer counts frequency using sequences from Illumina, Oxford Nanopore Technologies (ONT), and PacBio (PB) platforms for the bearded dragon *Pogona vitticeps*. Heterozygosity is high as indicated by dual peaks in each graph, and the height of the heterozygous peak increases with the length of the k-mer. This confirms diploidy.

Assembly

Hifiasm produced three assemblies: one for each haplotype and a pseudo-haplotype of high quality as evidenced by assembly metrics (Table 2). The haplotype assemblies were subject to further scaffolding and mis-join error correction using the HiC data to improve assembly contiguity (Figure S2). Minimal manual curation was required as wrongly joined scaffolds were corrected by YaHS (Figure S2). The reference assembly for the central bearded dragon had a

total length of 1,752,814,424 bp assembled into 89 scaffolds, with 26 gaps each marked by 100 Ns. This compares well with other published squamate genome assemblies.

The central bearded dragon reference genome (PviZW2.1) is contiguous with a scaffold N50 value of 266.2 Mbp and a N90 value of 28.1 Mbp with the largest scaffold of 358.3 Mbp (Table 2). L50 and L90 values were 3 and 9 respectively, typical of species with microchromosomes, where most of the genome is present in large macrochromosomes. The highly contiguous scaffolded assembly was obtained because the foundational contigs had an N50 value of 134.1 Mbp and an N90 value of 14.1 Mbp and a largest contig at 240.1 Mbp.

All 15 major scaffolds in the assembly (corresponding to autosome number in the karyotype of the bearded dragon) had well defined telomeres at each end (Figure 4). Telomeres were comprised of the vertebrate telomeric motif TTAGGG and ranged in size from 2,430 bp (405 copies of the repeat motif) to 42,098 bp (7,151 repeat copies). The telomeric regions were typically characterized by an expected rise in GC content (Figure 4) and a significant rise in inter-chromosomal contact (Figure 5; Figure S3), mirroring patterns previously described in turtles (Bista et al., 2024).

Initially, we did not detect telomere repeat sequence on 5’ end of the Scaffold 10 using a stringent threshold of 600 bp for telomeric region. However, manual examination revealed 32 repeats of telomeric sequence from position 1-214 on Scaffold 10 verifying that it had telomeres at both ends. The missing telomere for Scaffold 16 is expected because it is the pseudo-autosomal region of the sex chromosomes. The putative sex chromosome scaffolds 17 = W and 18 = Z also each possessed only one terminal telomeric sequence. This is consistent with T2T assembly for the sex chromosomes once the PAR and the non-recombining regions of Z and W are combined.

Typical centromeric satellite repeats units were not evident in the repeat structure, read depth profiles or GC content profiles (Figure 4) as they were for *Bassiana duperreyi* (Hanranan et al., 2025). Putative centromeric regions were evident for the macrochromosomes as an increase in the levels of inter-chromosomal contact in the HiC data and as a drop in heterozygosity (Figure 5 and Figure S3).

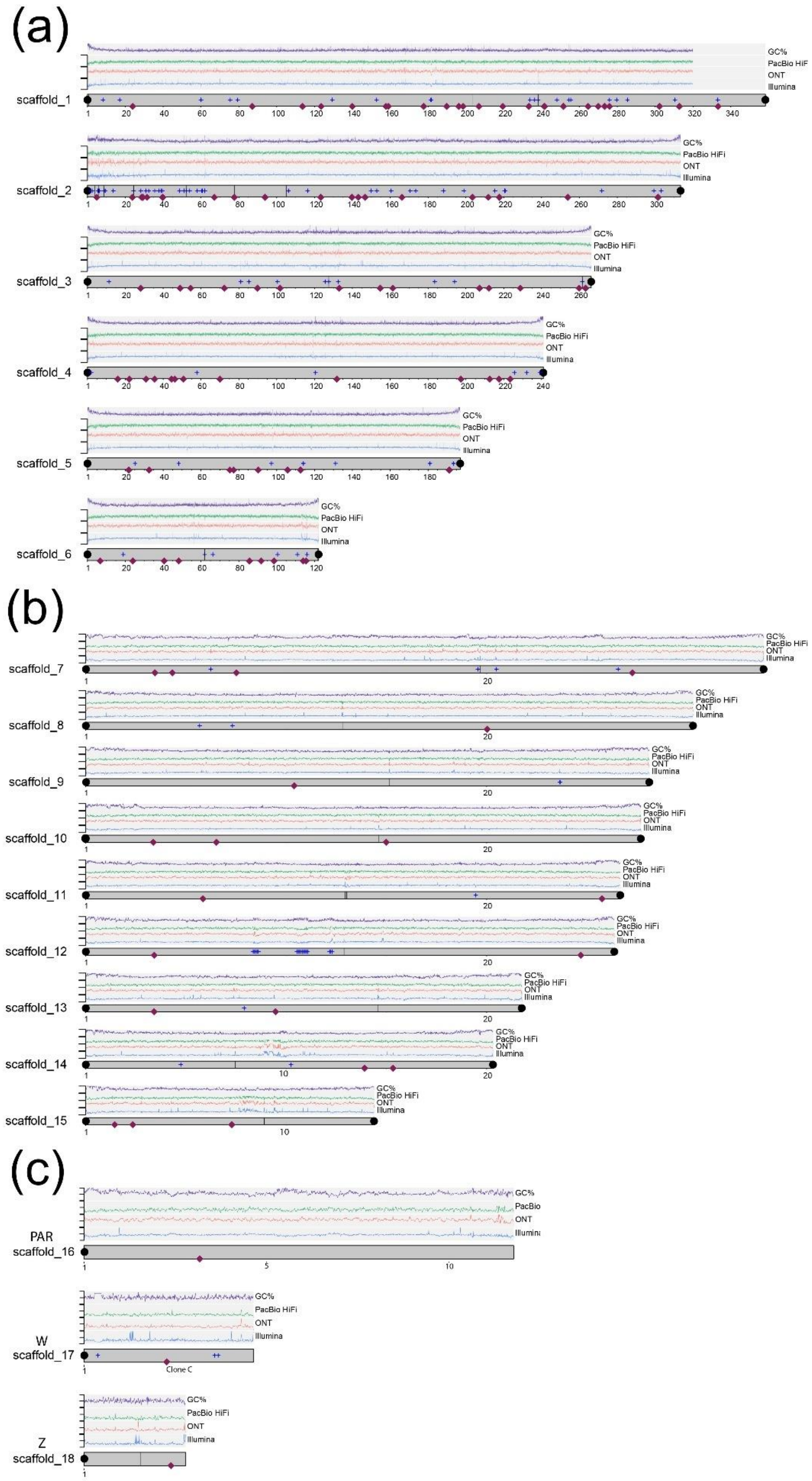
(a)
GC%
PacBio HiFi
ONT
Illumina
scaffold_1
scaffold_2
scaffold_3
scaffold_4
scaffold_5
scaffold_6
(b)
scaffold_7
scaffold_8
scaffold_9
scaffold_10
scaffold_11
scaffold_12
scaffold_13
scaffold_14
scaffold_15
(c)
PAR
scaffold_16
W
scaffold_17
Clone C
Z
scaffold_18

Figure 4. A plot of the 18 longest scaffolds (corresponding to the number of chromosomes of the bearded dragon *Pogona vitticeps*. Four traces are shown on each chromosome. The top trace (purple, range 30-60%) represents GC content, the next trace (green, range 0-50*x*) represents PacBio HiFi read depth, the next trace (red, range 0-100*x*) represents ONT read depth, and the fourth trace (blue, range 0-100*x*) represents Illumina read depth. Note that there is no indication in any of these traces of centromeric position in contrast to *Bassiana* (Hanrahan et al., 2025). Telomeres are shown as black dots; satellite repeats are indicated by the blue plus symbols (+); gaps by vertical black lines. The red diamonds show the location of BAC anchors (Young et al., 2013; Deakin et al., 2016, Table S7). Locations of the putative centromeres are shown in Figure 5. (a) Macrochromosomes; (b) Microchromosomes (c) both the Z and W specific regions were assembled into single scaffolds, with the PAR assembled into a single scaffold in both haplotypes. Refer to Supplementary Materials for a high-resolution version of this figure.

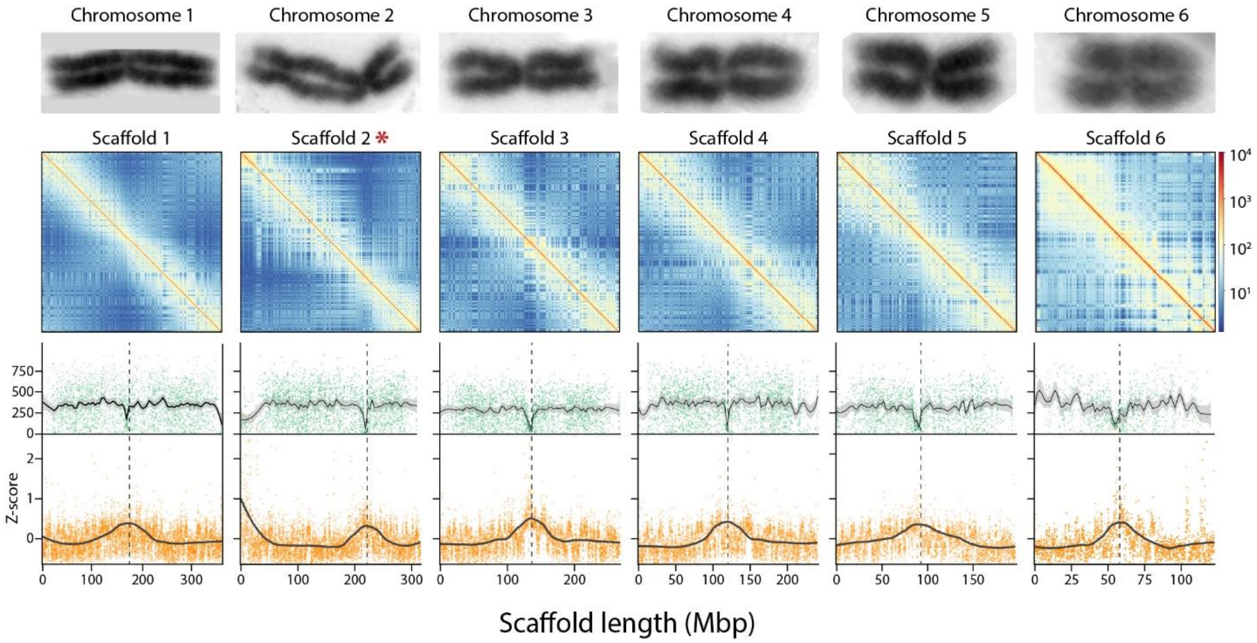

Figure 5. Identification of putative centromeres for the six macrochromosomes. The upper row of panels gives chromosome-specific Hi-C heatmaps showing intra-chromosomal interactions. The second row of panels shows the count of heterozygous sites per 50 Kbp window (green dots) with lines of best fit and 95% confidence interval (grey shading). The lower row of panels are the Z-scores for inter-chromosomal HiC interactions along chromosome length (Mbp) with smoothed lines of best fit. Each dot in the lower panels represents the Z-score interaction value of a different 50 Kbp bin. Chromosomes images are taken from Ezaz et al. (2005) and are not to scale. They are to illustrate the correspondence between the karyotype centromere and the putative position of the centromere (dashed lines) inferred from the dip in heterozygosity and the peak in inter-chromosomal contact. Scaffold 2 marked (*) is inverted with respect to the published karyotype. Refer to Figure S3 for similar plots for the microchromosomes.

Of 137 BAC clones (Young et al., 2013; Deakin et al., 2016), 5 with single sequences did not align, 2 had inter-chromosomal mappings, 14 had discrepant mappings for macrochromosomes and 2 had end sequences that were too far apart to be considered valid. This left 114 clones (83.2%) with reliable mappings. This physical mapping validated the assignment

of assembly scaffolds 1-6 to the macrochromosomes 1-6 of the genome (Figure 4a). The assignment of scaffolds 7-15 to the microchromosomes (Figure 4b) albeit with altered order (Figure 8), scaffold 16 to the PAR of the sex chromosomes (Figure 4c), and scaffold 18 as the nonrecombining region of the Z chromosome (Figure 4c). Mapping of the W-linked sequence Clone C1 (3,288 bp, Quinn et al., 2010) confirmed the identity of scaffold 17 as the non-recombining region of the W chromosome (Figure 4c).

Assembly evaluation

The percent collapsed sequence in the assembly was exceptionally low at 0.003% (492,971 bp, 54-13,643 bp, n=255) as was the percentage of false expansions at 0.03% (49,447 bp, 52-5,133 bp, n=69); two of the indicators of genome assembly quality identified by the Earth Biogenome Project (Lawniczak et al., 2022).

Completeness of the assembly was estimated to be 99.82% for both haplotype assemblies combined and the per base assembly quality estimate exceeded Q40 at 48.36 (1 error in 68.5 Kbp). High heterozygosity in the k-mer profiles (Figure 3) affects assembly completeness metrics measured by *Merqury*. Individual haplotype assemblies were 85.5% complete, which is expected of animals with high heterozygosity (in our case, 1.98%). This shows that assembly completeness metrics for a single haplotype assembly measured using k-mers can be understated for species with high heterozygosity.

Analyses using the Benchmarking Universal Single-Copy Orthologs (BUSCO) gene set for Sauropsids reveals 7,321 genes as complete (97.9%), with a minimal proportion duplicated (D: 1.1%), indicating a robust genomic structure with minimal redundancy (Figure 7). The central bearded dragon genome also had a low proportion of fragmented (F: 0.5%) and missing (M: 1.6%) orthologs. These results positioned central bearded dragon favorably in terms of genome completeness and integrity, on par with other squamates, and highlights its potential as a reference for further genomic and evolutionary studies within this phylogenetic group. In our comparison set, only chicken (*Gallus gallus*) has better BUSCO statistics than the bearded dragon. RNAseq data mappability was on average 93.5% and 18 of 22 samples had more than 90% of fragments mapped to the genome (Table S2). Note that sensitivity settings for alignments had to be increased for mapping RNAseq data given high hetergozygosity observed for this species (1.98%).

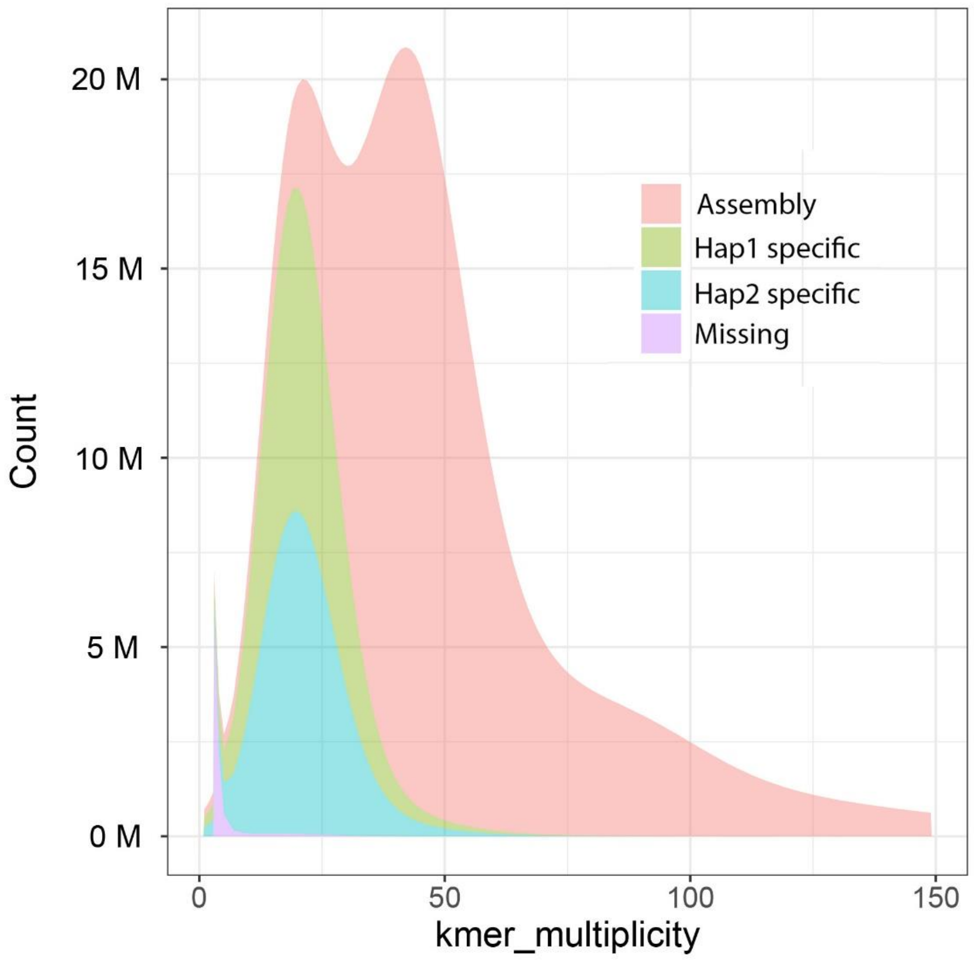

Figure 6. Distribution of Illumina k-mers (k = 17) in the genome assembly of the bearded dragon *Pogona vitticeps* (Table S6). K-mer counts are shown on the x-axis and the frequency of occurrence of those counts on the y-axis. Those scored as missing are found in reads only.

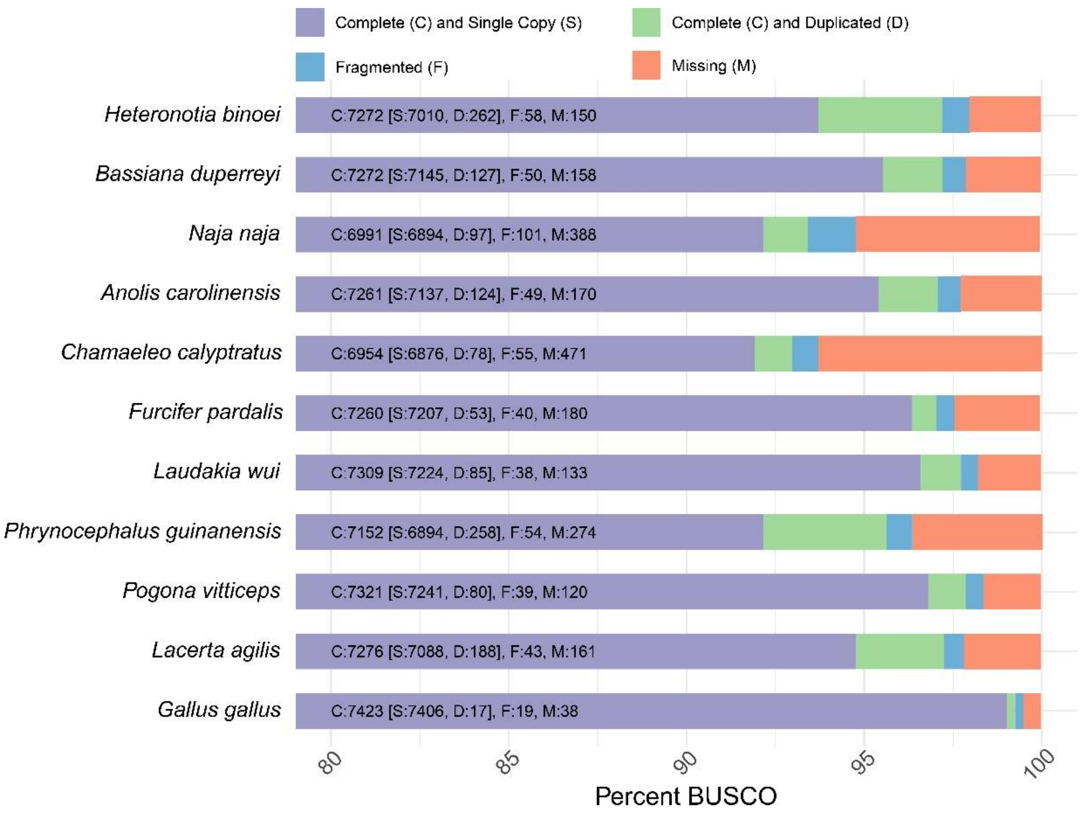

Figure 7. A visual representation of how complete the gene content is for each listed species genome, including *Pogona vitticeps*, based on Benchmarking Universal Single-Copy Orthologs (BUSCO, n=7480).

Chromosome Assembly

The bearded dragon has 2n=32 chromosomes with six pairs of macrochromosomes and ten pairs of microchromosomes including the sex chromosomes. The distinction between macro and microchromosomes typically relies on a bimodal distribution of size, however other characteristics such as GC content provide additional evidence for this classification (Waters et al., 2021; Bista et al., 2024) (Figure 8). The median GC content of 10 Kbp windows for the six largest scaffolds (representing macrochromosomes) ranged between 40.7% and 41.8% . In contrast, the remaining 12 scaffolds ordered by decreasing length had a median GC content of between 42.6% and 47.6% characteristic of microchromosomes in other squamates.

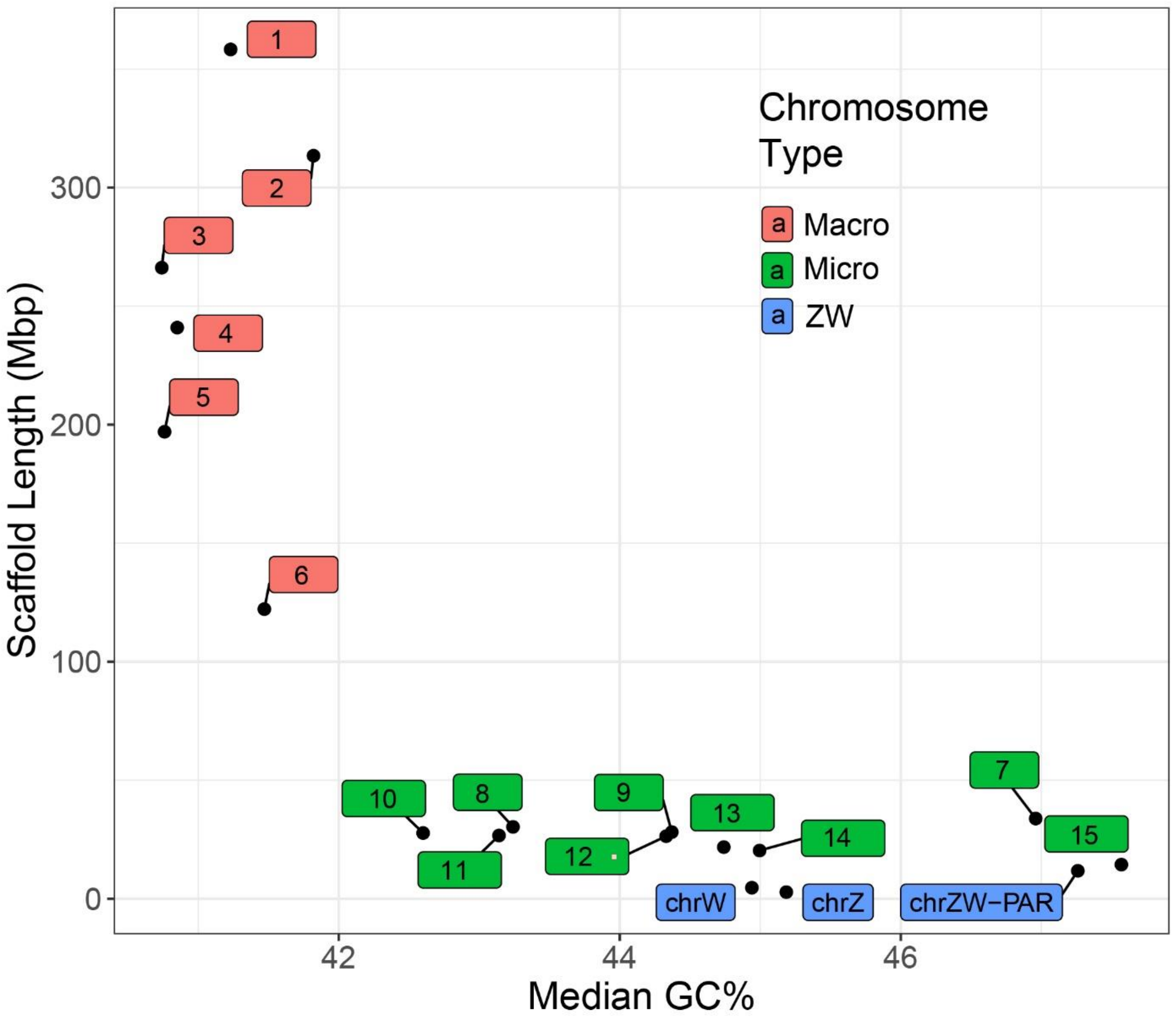

Figure 8. A plot of assembly scaffolds defined by scaffold length vs median GC content in 10 Kbp windows. Microchromosomes are characterised by higher GC content than macrochromosomes. Median GC content in 10 Kbp windows of scaffolds vs length of scaffolds representing macrochromosomes (scaffolds 1-6, red), the sex chromosomes (blue, the PAR and nonrecombining regions of the Z and W) and the other microchomosomes (green, scaffolds 7-15). Scaffold numbers 1-6 correspond to the macrochromosome numbers of Deakin et al. (2016) for scaffolds. Scaffold numbers 7-15 translate to the microchromosome numbers of Deakin et al. as per Table S6.

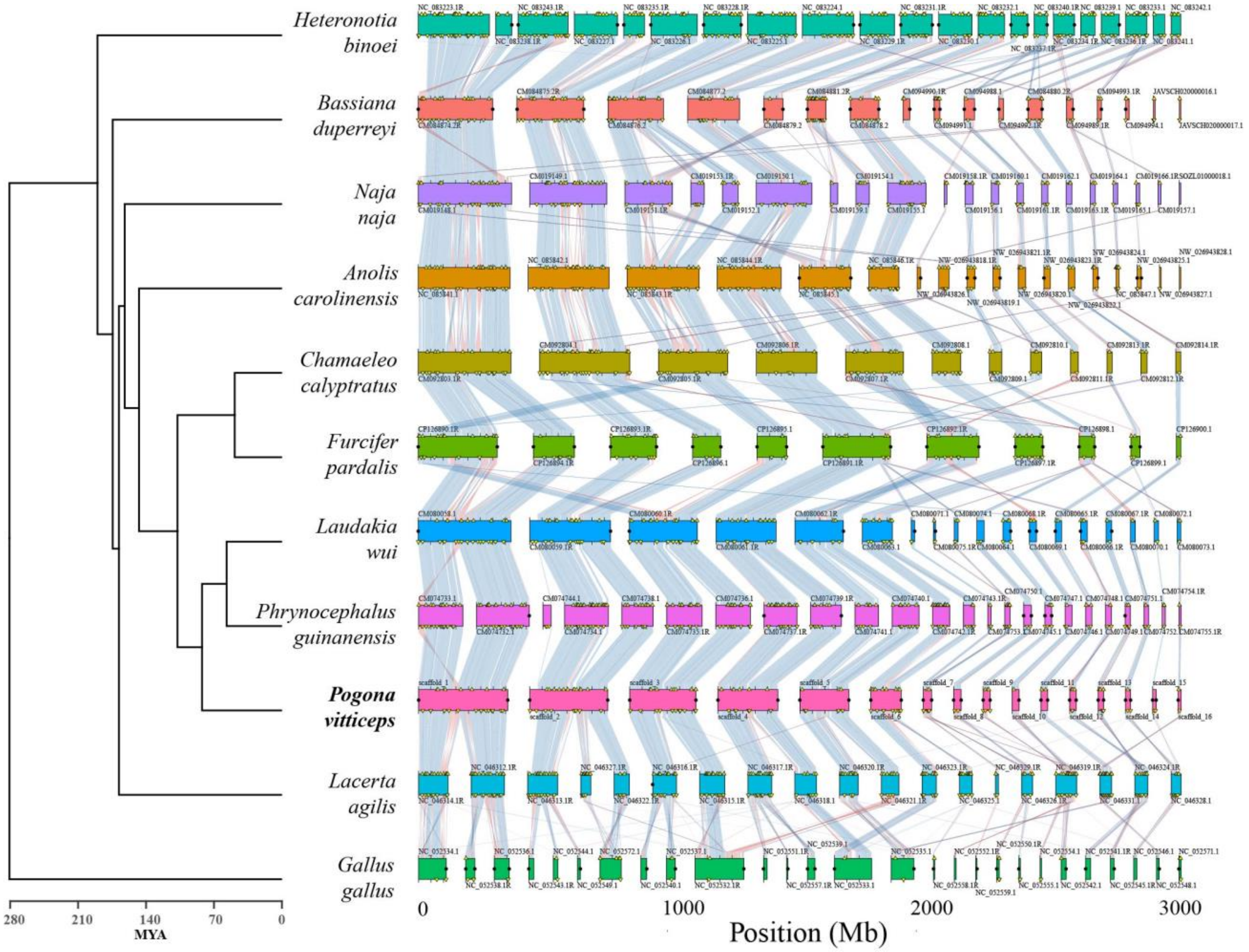

Figure 9. Synteny conservation of BUSCO homologs for the bearded dragon *Pogona vitticeps* and squamates with chromosome level assemblies including representative skink, iguanid, snake and gecko lineages and chicken. Synteny blocks corresponding to each species are aligned horizontally, highlighting conserved chromosomal segments across the genomes. The syntenic blocks are connected by ribbons that represent homologous regions shared between species, with the varying colours denoting segments of inverted gene order. Duplicated BUSCO genes are marked with yellow triangles. Predicted telomeres are marked with black circles.

Unlike mammals, reptiles (including most birds) show a high level of chromosomal homology across species (Waters et al., 2021; Bista et al., 2024). Figure 9 shows synteny conservation between bearded dragon, representative squamate species and chicken. Apart from a handful of intrachromosomal rearrangements, the major scaffolds of bearded dragon and other squamates corresponded well, including the pseudoautosomal region (PAR) of the sex microchromosomes (scaffold 16) within the Agamidae. When compared with other genomes in the analysis, the bearded dragon genome showed a high degree of evolutionary conservation with respect to both chromosomal arrangement and gene order (Figure 9).

The Z and W specific sex chromosome scaffolds were identified as 18 and 17, respectively. These represent the non-recombining region of the sex chromosomes. They were not assembled to the PAR in either haplotype despite the use of HiC data for scaffolding. The Z specific scaffold was 2.78 Mbp and W specific scaffold was 4.64 Mbp. In the sequenced ZW female, read depth for both scaffolds were identified based on the median read depth in 10 Kbp sliding windows. As expected, read depth was approximately half that of the autosomes and the PAR scaffold (Figure 10a). The first half of the Z and W scaffolds share good homology (Figure 10b). On the second half of the W scaffold there appears to have been duplication and expansion that increased its size relative to the Z. The PAR scaffold (scaffold 16 reference, 11.77 Mbp) was identified by homology to known Z sequences from Pvi1.0.

The W specific scaffold had seven annotated genes, with none presenting as an obvious sex determining candidate. The Z specific scaffold also had seven annotated genes, four of which were ZW shared (Figure 10b). Notably, copies of both *Amh* and its receptor (*AmhR2*) were located on the Z, presumably duplicated from the autosomal homologues which remain present on scaffolds 7 and 2 respectively. Both genes are central to the sex determining pathway in other vertebrates, so present as strong sex determining candidates that would presumably function in a dosage dependent manner.

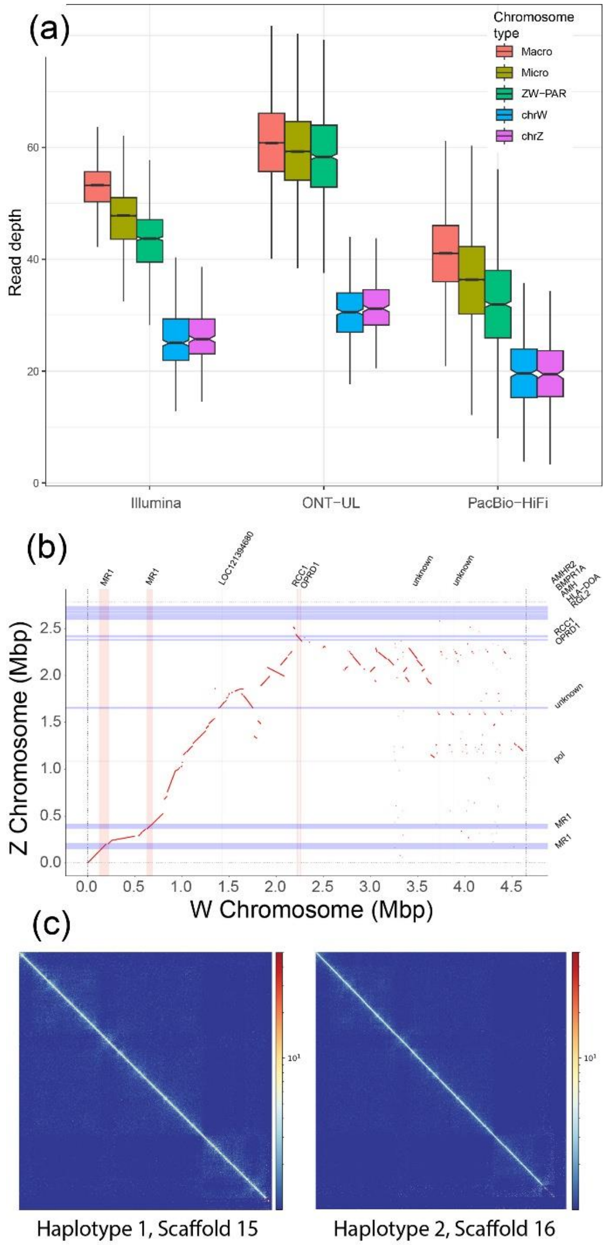

Figure 10. Sex chromosome analysis. a) For each sequencing technology, boxplots of read depth in 10 Kbp windows of macrochromosomes, microchromosomes, the PAR, and Z and W specific scaffolds. Boxes represent the middle 50% of the data, notches represent 95% confidence intervals of the medians (central horizontal black bar), whiskers are +/- 1.5 the interquartile range, outliers not plotted. b) Alignment of the Z specific (y-axis) and W specific (x-axis) scaffolds. Red lines represent homologies. Blue horizonal bars are genes annotated on the Z scaffold (gene names given on the y-axis), pink vertical bars are genes annotated on the W scaffold (gene names given on the x-axis). c) Phase HiC contact maps of the PAR in the two different haplotypes. Note that it is unknown which is the Z PAR and which is the W PAR.

HiC reads were phased to the PAR scaffolds to determine if there was different 3D structure of the Z and W scaffolds (see Zhang et al., 2022). Surprisingly, despite clear cytological differences between the Z and W in cultured fibroblasts (Ezaz et al., 2005), there was little difference between HiC contact maps for each haplotype (Figure 10c). The discrepancy between cytogenetic (fibroblasts) and HiC data (blood) with respect to the Z and W structure, likely arise from the different cell types examined. Alternatively, the cytogenetic data only captures cells in metaphase when three-dimensional genome structure differences might be at their most pronounced.

Annotation

General Repeat Annotation

An estimated 45.6% (798 Mbp) of the bearded dragon genome was composed of repetitive sequences, including interspersed repeats, small RNAs and simple and low complexity tandem repeats (Table 9). Retroelements (SINEs and LINEs) were the most common repetitive element (17.3%). DNA transposons were the second most common repetitive element (5.1%) and are dominated by Tc-Mar and hAT elements (Table S8). CR1, BovB and L2 elements were the dominant long interspersed elements (81% of LINE elements; 13.4% of the genome), which is consistent with other squamate genomes (Pasquesi et al., 2018). A total of 37% of all repeat content was unclassified and did not correspond to any element in the RepeatModeler libraries. Refer to Figure S4 for the size distribution of these unclassified repeats. The number of elements masked and their relative abundances are presented in the supplementary material (Table S8).

Table 9. Condensed summary of the copy number and percentage of the bearded dragon (*Pogona vitticeps*) genome covered by repeat elements. Refer to Table S8 for full breakdown.

Family	Numbers of elements	Length masked (bp)	% of sequence
Retroelements	1,061,885	303,713,020	17.33
SINEs	114,787	15,769,671	0.90
LINEs	947,098	287,943,349	16.43
LTR elements	80,454	72,706,738	4.15
DNA transposons	432,771	89,795,030	5.12
Penelope-like elements	1,797	150,905	0.01
Rolling-circles	447	95,044	0.01
Unclassified retroelements	1,775,114	294,309,581	16.79
**Total interspersed repeats**	**3,352,468**	**760,770,318**	**43.40**
Satellite	4,246	1,284,141	0.07
Simple Repeat	576,326	34,276,116	1.96
rRNA	402	1,812,567	0.10
snRNA	2,397	502,447	0.03
tRNA	101	6,775	0.00
**Total Masked**		**798,652,364**	**45.56**

Satellite repeats

We undertook a more detailed analysis in an attempt to identify centromeric satellite repeats and centromeric regions as we did for the genome assembly of the skink *Bassiana duperreyi* (Hanrahan et al., 2025). The 67 satellite repeat units identified in the KMC/SRF analysis had lengths between 5 bp and 9,460 bp. These collapsed into 45 distinct classes based on sequence similarity (Table S9).

One repeat class (srfclass-16) corresponded to the telomeric microsatellite repeats (TTAGGG). A second class (srfclass-18) with a large repeat unit of 9,460 bp corresponded to ribosomal DNA sequence, dealt with in more detail later. A class of interspersed repeat (srfclass-11), possibly LINE elements, was comprised of 5,695 bp in unit length. A class of repeat

(srfclass-30) was present as 84 copies on Scaffold 4 (119,114,420-119,423,712); all other copies were interspersed across the genome. A telomeric repeat was embedded in this larger repeat. A fifth class (srfclass-21) comprised repeat units of 2,190 bp on Scaffold 1 (117 copies, 167,719,827-167,977,892) and was somewhat enigmatic. These units were tandemly organized as 1 to 43 repeats, occasionally with a small intervening sequence. This repetitive sequence was found also on other scaffolds as interspersed units comprising a 1,406 bp motif and a 606 bp motif separated by 500 bp intervening sequence. A sixth class (srfclass-38) comprised repeat units of 877 bp, found only on scaffold 1 (238 copies, 54,463,716 – 254,497,379). A seventh class (srfclass-15) comprised repeat units of 398 bp, each as a composite of a 68 bp subunit, on scaffold 1 (181407117- 181830275, *ca* 1063 copies). These repeat units align with elements on Scaffolds 3, 4 and 5, but with abbreviated subunits (e.g. 56 bp on Scaffold 3; 64 bp on Scaffold 4). An eighth repeat class (srfclass-4) comprised a 98 bp motif occurred on the W chromosome scaffold 17 (1,961 copies, 281,785-473,916) found as 109-450 bp alignments on other scaffolds. Of the 45 repeat classes, only one (srfclass-5, 151 bp) showed potential as a centromeric repeat unit. However, this repeat class was not distributed as a single consolidated cluster on each chromosome, as would be expected of centromeric repeat units.

We were thus unable to definitively identify centromeric repeat units in the bearded dragon to confirm the presence of only one per chromosomal scaffold as we were able to do in the skink *Bassiana duperreyi* (Hanrahan et al., 2025). We were however able to confirm the likely presence of one centromere per scaffold as expected if the scaffolds correspond to chromosomes using plots of heterozygosity and an index of inter-chromosomal contact rates against position on the scaffold (Figure 5). A dip in heterozygosity corresponded with a peak in HiC inter-chromosomal contact rate which together corresponded well with the position of the centromere taken from metaphase chromosomal spreads (Ezaz et al., 2005).

Gene Annotation

We assembled transcriptomes from 22 samples (Table S2). Genome annotation using *Augustus* predicted 17,237 genes and transcripts, of which 16,799 had a match to a Uniprot_Swissprot or Uniprot_TrEMBL protein sequence, and 16,483 were assigned a gene name. Analyses using the BUSCO gene set (v5.8.2, *n*=7480 genes) for Sauropsids reveals 6,558 genes as complete (87.7%), with 1.3% duplicates. There were 237 genes (3.2%) identified as fragmented and a

further 685 genes (9.2%) as missing in the annotated protein sequences. These data suggest relatively accurate gene prediction. The quality of the annotation was further validated using RNAseq data from 22 samples, with an average 54.4% (ranging from 22.8% to 76.4%) of aligned reads assigned to annotated exons, indicating a reasonable level of correspondence between the predicted gene models and the observed transcriptomes. Relatively lower mapping rates of RNAseq data can be attributed to incomplete annotations of untranslated regions and the presence of other non-coding RNAs that are not annotated. Further improvements to annotations in the future will likely improve the utility of this genomic resource.

Mitochondrial Genome

The bearded dragon mitochondrial genome assembly was 16,731 bp in size with 37 intact genes without frameshift mutations. It consisted of 22 tRNAs, 13 protein coding genes, 2 ribosomal RNA genes and the control region (Figure S5), so was typical of the vertebrate mitochondrial genome. Base composition was A = 33.0%, C = 29.8%, G = 13.1% and T = 24.0%. Illumina data showed only 12 homopolymer variant sites suggesting accurate recovery of the mitochondrial sequence.

We note that mitochondrial sequence was absent in the HiFi data presumably because it was eliminated during the size selection step. As the assembly software uses PacBio HiFi for the core assembly, these mitochondrial sequences, although present in the ONT data, were not recovered during the combined assembly process. We also note a drop in the read depth for the PacBio HiFi and Illumina data for exceptionally small microchromosomes (Figure 10a) that is not observed for ONT data. This suggests a systematic bias in the data from sequence-by-synthesis platforms for small elements and high GC content sequences.

Ribosomal DNA

The rDNA unit length in the bearded dragon is approximately 9.5 Kbp, with a total of 1.75 Mbp of sequence across 24 scaffolds containing rDNA sequences. The rDNA sequence was found on chromosome 2 scaffold as expected (Young et al., 2013) near the sub-telomeric region of 2q. There were 23 additional short scaffolds comprised entirely of rDNA arrays as well indicating poor quality assembly of rDNA array. The first and second internal transcribed spacers (533 bp

and 344 bp respectively) and intergenic spacer (2.7 Kbp) are relatively small, compared to mammals (McDonald et al., 2024).

Conclusion

Here we present a high-quality genome assembly of the central bearded dragon, *Pogona vitticeps* (Ahl, 1926). The quality of the genome assembly and annotation compares well with other chromosome-length assemblies and is among the best for any species of Agamidae. We have chromosome length scaffolds, and near telomere-to-telomere quality.

The non-recombining regions of the Z and W chromosomes were each assembled as a single scaffold. The PAR was assembled as a single scaffold in both haplotypes. The sex chromosomes scaffolds and PAR scaffold each lacked one telomere, but this is likely resolved when they are combined to form Z and W scaffolds including both the PAR and non-recombining regions. The identification of *Amh* and *Amhr2* on the Z specific scaffold (but not the W) has them as strong candidates for the sex determining gene(s) in this species. This will be a fruitful area for future functional studies based on upregulation and knockout technologies (e.g. CRISPR knockouts). Such approaches were necessary to establish the *Sry* gene on the Y chromosome of eutherian mammals as the master sex determining gene acting through dominance (Sinclair et al., 1990; Koopman et al., 1991), and to establish *Drmt1* on the Z chromosomes of birds as the master sex determining gene acting though dosage (Smith et al., 2009; Lambeth et al., 2014). Gene *Nr5a1,* encoding transcription factor SF1, was previously identified as a candidate sex determining gene because it resided on the sex chromosomes and because of its differential transcript isoform composition (Zhang et al., 2022); it is confirmed as residing within the PAR on both the Z and W chromosomes. The concurrent discovery of *Amh* and *Amhr2* as duplicate copies of their autosomal orthologs (see Guo et al., 2025 GigaScience, this issue) on the Z chromosome and confirmation here that they do not reside on the W, hints at a dosage-based mechanism of sex determination involving one or both of these genes. *Amh* is a gene and its receptor *AmhR2* are central to male differentiation in vertebrates and so are predisposed to recruitment as master sex determining genes on the sex chromosomes. This has occurred multiple times in fish with the enlistment of *Amh* or *AmhR2* to the Y chromosome (Li et al., 2015; Song et al., 2021; Nakamoto et al., 2021; Jeffries et al., 2022) or the involvement of *Amh* in the establishment of a *de novo* sex chromosome (Kamiya et al., 2012). In the frog *Rana*

temporaria, the Y chromosome underwent a reciprocal translocation with an autosome fusing them into a single inherited neo-Y chromosome that included key sex genes *Dmrt1*, *Amh*, and *AmhR2* (Rodrigues et al., 2016). *Amh* is also implicated as the master sex determining gene in monotremes (Zhou et al., 2021). Our results indicate that sex determination in the dragon is likely involve more complex gene interactions, involving expression of the Z and autosomal copies of *Amh* and *AmhR2* and involving also *Nr5a1* which encodes transcription factor SF1 and has a foundational involvement in sex determination in vertebrates. The gene *Nr5a1*, although on the PAR as confirmed here, and with virtually identical copies on the Z and W chromosomes, yields substantially different Z and W transcriptional isoform composition (Zhang et al., 2022). This suggests that complex interactions between these genes and their resultant transcription factors and intermediaries, determines sex in the bearded dragon. This will be a fruitful area for future investigation.

This annotated assembly for the central bearded dragon was generated as part of the AusARG initiative of Bioplatforms Australia, to contribute to the suite of high-quality genomes available for Australian reptiles and amphibians as a national resource. The central bearded dragon is already widely used in research requiring genomic foundations, in large part because of the earlier publication of an assembly based on short read technologies (Georges et al., 2015). The central bearded dragon is an emerging model species (Ollonen et al., 2018) because of its high fecundity and short incubation, ease with which it adapts to captivity and a published genome, all considered key advantages accelerating its use (Infante et al., 2018). We anticipate that this new and vastly improved reference genome will serve to accelerate comparative genomics, developmental studies and evolutionary research on this and other species. As an exemplar of a well-studied oviparous taxon with sex reversal by temperature, the central bearded dragon reference assembly will provide a solid basis for genomic studies of the evolution of the genetic basis for reprogramming of sexual development under the influence of environmental temperature (Quinn et al., 2007; Holleley, et al., 2015; Castelli et al., 2021).

Editors' Note

Another near-T2T bearded dragon genome assembly is published alongside this work, sequenced using different sequencing technology and approaches (Guo et al., 2025).

Funding

This work was supported by the AusARG initiative funded by Bioplatforms Australia, the Australian Research Council (DP220101429) and the National Health and Medical Research Council (APP2021172). A.R.-H. acknowledges the Spanish Ministry of Science and Innovation (PID2020-112557GB-I00 funded by AEI/10.13039/501100011033), the Agència de Gestió d'Ajuts Universitaris i de Recerca, AGAUR (2021SGR00122) and the Catalan Institution for Research and Advanced Studies (ICREA). L.M.-G. was supported by an FPU predoctoral fellowship from the Spanish Ministry of Science, Innovation and University (FPU18/03867 and EST22/00661).

Data Availability

The supplementary file contains a description of all supplemental materials, which include tables showing software used in the preparation of this paper, outcomes of the sequencing on the four sequencing platforms used, and figures in support of statements on the quality of data. The authors affirm that all other data necessary for confirming the conclusions of the article are present within the article, figures, and tables. The annotated assembly can be accessed from NCBI as PviZW2.1 and all reads used in support of the assembly are lodged with the Short Read Archive. All sequence data generated in this study are available from NCBI SRA under BioProject ID PRJNA1252275. Accession numbers are provided in the main text and the Supplementary Tables (Tables S2-S6). High resolution versions of figures and custom scripts used to conduct the analyses are at https://github.com/kango2/ausarg/. Additional data is available in the GigaDB repository (Patel et a., 2025).

Abbreviations

BAC	Bacterial Artificial Chromosome
BUSCO	Benchmarking Universal Single-Copy Orthologs
EBP	Earth BioGenome Project
HiC	High-throughput Chromosome Conformation Capture
HiFi	High Fidelity
L50	min number of contigs (or scaffolds) to add in length to 50% of assembly length
L90	min number of contigs (or scaffolds) to add in length to 90% of assembly length
LINE	Long Interspersed Nuclear Element
LTR	Long Terminal Repeat

N50	median (50th percentile) contig or scaffold length
N90	90th percentile of contig or scaffold length
NCBI	The National Center for Biotechnology information
ONT	Oxford Nanopore Technologies
PacBio	Pacific Biosciences
PAR	pseudoautosomal region
PCR	polymerase chain reaction
Q20	Phred score of 20 corresponding to a 1% error rate
Q30	Phred score of 30 corresponding to a 0.01% error rate
rDNA	ribosomal DNA
RNAseq	RNA-sequencing
rRNA	ribosomal RNA
SINE	Short Interspersed Nuclear Element
snRNA	small nuclear RNA
T2T	telomere-to-telomere
tRNA	transfer RNA
UTR	Untranslated Region

Author Contributions

All authors contributed to the writing and editing of drafts of this manuscript. In addition, A.G. was the AusARG project lead and responsible for coordinating the initial proposal and securing the funding; A.L.M-R. contributed to the development of assembly pipelines; D.S.B.D – prepared samples, constructed figures and contributed to the initial conceptual work; H.R.P. led the assembly and development of related workflows and pipelines; I.W.D. and J.H. provided oversight of the data generation and supervision of subsequent analysis; J.K.C. developed the annotation workflow and pipelines; N.C.L performed phased HiC analyses. N.C.L. and H.J. examined variation between the haplotypes and the reference haplotype for analysis of trends in heterozygosity. Z.A.C. undertook the rDNA annotation. H.R.P oversaw the data generation, associated quality control and the submission to NCBI; K.A. was responsible under the supervision of H.R.P for data curation and management, constructing the automated assembly and annotation workflows, for the manual curation of the assembly & analysis and post-assembly analysis; P.D.W. with H.R.P. provided oversight of the assembly and annotation, interpretation of the Z and W scaffolds. L.X., C.E.H., S.W. and X.Z. contributed to interpretation of the sex chromosome genes and the implications for future work. A.R-H. and L.M-G. conducted chromosome contact analysis.

Acknowledgements

We acknowledge the provision of computing and data resources provided by the Australian BioCommons Leadership Share (ABLeS) program. This program is co-funded by Bioplatforms Australia (enabled by the National Collaborative Research Infrastructure Strategy, NCRIS) and the National Computational Infrastructure (NCI).

Competing interest

H.R.P., I.W.D., A.L.M-R., A.G. have previously received travel and accommodation expenses from ONT and/or PacBio to speak at conferences. I.W.D. has a paid consultant role with Sequin Pty Ltd. The authors declare no other competing interests.

References

Álvarez-González, L., Arias-Sardá, C., Montes-Espuña, L., Marín-Gual, L., Vara, C., Lister, N.C., Cuartero, Y., Garcia, F., Deakin, J., Renfree, M.B., Robinson, T.J., Martí-Renom, M.A., Waters, P.D., Farré, M., Ruiz-Herrera, A. 2022. Principles of 3D chromosome folding and evolutionary genome reshuffling in mammals. Cell Reports 41: 111839. doi: 10.1016/j.celrep.2022.111839.

Amer, S.A.M., Kumazawa, Y. 2005. Mitochondrial genome of *Pogona vitticepes* (Reptilia; Agamidae): control region duplication and the origin of Australasian agamids. Gene 346: 249-256. https://doi.org/10.1016/j.gene.2004.11.014.

Arima Genomics. The Arima Genomics alignment pipeline, v03. GitHub. 2024. https://github.com/ArimaGenomics/mapping_pipeline accessed 16-Apr-2025

Benson, G. 1999. Tandem repeats finder: a program to analyze DNA sequences. Nucleic Acids Research 27 :573–580. doi:10.1093/nar/27.2.573.

Bista, B., González-Rodelas, L., Álvarez-González, L., Wu, Z.Q, Montiel, E.E., Lee, L.S., Badenhorst, D.B., Radhakrishnan, S., Literman, R., Navarro-Dominguez, B., Iverson, J.B., Orozco-Arias, S., González, J., Ruiz-Herrera, A., Valenzuela, N. 2024. De novo genome assemblies of two cryptodiran turtles with ZZ/ZW and XX/XY sex chromosomes provide insights into patterns of genome reshuffling and uncover novel

3D genome folding in amniotes. Genome Research 34: 1553-1569. doi: 10.1101/gr.279443.124.

Bolger, A.M., Lohse, M., Usadel, B. 2014. Trimmomatic: a flexible trimmer for Illumina sequence data. *Bioinformatics* 30: 2114–2120. https://doi.org/10.1093/bioinformatics/btu170.

Bonnan, M.F., Crisp, L.M., Barton,A. Dizinno, J.,Muller, K., Smith, J., Walker, J. 2024. Exploring elbow kinematics in the central bearded dragon (*Pogona vitticeps*) using XROMM: Implications for the role of forearm long-axis rotation in non-avian reptile posture and mobility. The Anatomical Record. https://doi.org/10.1002/ar.25588.

Buchfink, B., Reuter, K., Drost, H.G. 2021. Sensitive protein alignments at tree-of-life scale using DIAMOND. Nature Methods 18:366–368. doi:10.1038/s41592-021-01101-x.

Chandrasekara, U., Mancuso, M., Sumner, J., Edwards, D., Zdenek, C.N., Fry, B.G. 2024. Sugar-coated survival: N-glycosylation as a unique bearded dragon venom resistance trait within Australian agamid lizards. Comparative Biochemistry and Physiology C: Toxicology & Pharmacology 282: 109929, https://doi.org/10.1016/j.cbpc.2024.109929.

Castelli, M., Georges, A., Cherryh, C., Rosauer, D., Sarre, S.D., Contador-Kelsall, I. and Holleley, C.E. 2021. Evolving thermal thresholds may explain the distribution of temperature sex reversal in an Australian dragon lizard (*Pogona vitticeps*). Diversity and Distributions 27: 427-438. https://doi.org/10.1111/ddi.13203.

Chen, Y., Zhang, Y., Wang, A.Y. Gao, M., Chong, Z. 2021. Accurate long-read de novo assembly evaluation with Inspector. Genome Biology 22: 312. https://doi.org/10.1186/s13059-021-02527-4.

Cheng, H., Concepcion, G.T., Feng, X. et al. 2021. Haplotype-resolved de novo assembly using phased assembly graphs with hifiasm. Nature Methods 18: 170–175. https://doi.org/10.1038/s41592-020-01056-5.

Cheng, H., Jarvis, E.D., Fedrigo, O., Koepfli, K.P., Urban, L., Gemmell, N.J., Li, H. 2022. Haplotype-resolved assembly of diploid genomes without parental data. Nature Biotechnology 40: 1332–1335. https://doi.org/10.1038/s41587-022-01261-x.

Cleveland, W.S. 1979. Robust Locally Weighted Regression and Smoothing Scatterplots. Journal of the American Statistical Association 74: 829-836.

Cogger, H.G. 2018. Reptiles and Amphibians of Australia (7th updated ed.). Melbourne: CSIRO Publishing.

Camacho, C., Coulouris, G., Avagyan, V., Ma, N., Papadopoulos, J., Bealer, K., Madden, T.L. 2009. BLAST+: architecture and applications. BMC Bioinformatics, 10: 421. https://doi.org/10.1186/1471-2105-10-421.

Dainat, J. (2022). AGAT: Another Gff Analysis Toolkit to handle annotations in any GTF/GFF format. (Version v0.7.0). Zenodo. https://www.doi.org/10.5281/zenodo.3552717.

Danecek, P., Bonfield, J.K., Liddle, J., Marshall, J., Ohan, V., Pollard, M.O., Whitwham, A., Keane, T., McCarthy, S.A., Davies, R.M., Li, H. 2021. Twelve years of SAMtools and BCFtools. *GigaScience* 10: giab008. https://doi.org/10.1093/gigascience/giab008.

Deakin, J., Edwards, M.J., Patel, H., O'Meally, D., Lian, J., Stenhouse, R., Ryan, S., Livernois, A., Azad, B., Holleley, C., Li, Q. and Georges, A. 2016. Anchoring genome sequence to chromosomes of the central bearded dragon (*Pogona vitticeps*) enables reconstruction of ancestral squamate macrochromosomes and identifies sequence content of the Z chromosome. BMC Genomics 17: 447. https://doi.org/10.1186/s12864-016-2774-3.

Durand, N.C., Shamim, M.S., Machol, I., Rao, S.S.P., Huntley, M.H., Lander, E.S., Aiden, E.L. 2016. Juicer provides a one-click system for analyzing loop-resolution Hi-C experiments. Cell Systems 3: 95-98. doi: 10.1016/j.cels.2016.07.002.

Edwards, R.J., Dong, C., Park, R.F., Tobias, P.A. 2022. A phased chromosome-level genome and full mitochondrial sequence for the dikaryotic myrtle rust pathogen, *Austropuccinia psidii*. bioRxiv 2022.04.22.489119 doi: 10.1101/2022.04.22.489119.

Ezaz, T., Quinn, A.E., Miura, I., Sarre, S.D., Georges, A., Graves, J.A.M. 2005. The dragon lizard *Pogona vitticeps* has ZZ/ZW micro-sex chromosomes. Chromosome Research 13: 763-776. https://doi.org/10.1007/s10577-005-1010-9.

Fenk, L.A., Riquelme, J.L., Laurent, G. 2024. Central pattern generator control of a vertebrate ultradian sleep rhythm. Nature 636: 681–689. https://doi.org/10.1038/s41586-024-08162-w.

Fu, L., Niu, B., Zhu, Z., Wu, S., Li, W. 2013. CD-HIT: accelerated for clustering the next-generation sequencing data. Bioinformatics. 28: 3150-152. https://doi.org/10.1093/bioinformatics/bts565.

Georges, A., Li, Q., Lian, J., O'Meally, D., Deakin, J., Wang, Z., Zhang, P., Fujita, M., Patel, H.R., Holleley, C.E., Zhou, Y., Zhang, X., Matsurbara, K., Waters, P., Graves, J.A.M., Sarre, S.D., Zhang, G. 2015. High-coverage sequencing and annotated assembly of the genome of the Australian dragon lizard *Pogona vitticeps*. GigaScience 4: 45. https://doi.org/10.1186/s13742-015-0085-2.

Grabherr, M.G., Haas, B.J., Yassour, M., Levin, J.Z., Thompson, D..A, Amit, I., Adiconis, X, Fan L., Raychowdhury, R., Zeng, Q., Chen, Z., Mauceli, E., Hacohen, N., Gnirke, A., Rhind, N., di Palma, F., Birren, B.W., Nusbaum, C., Lindblad-Toh, K., Friedman, N., Regev, A. 2011. Full-length transcriptome assembly from RNA-seq data without a reference genome. *Nature Biotechnology* 29: 644-52. https://doi.org/10.1038/nbt.1883.

Gremme, G., Steinbiss, S., Kurtz, S. 2013. GenomeTools: a comprehensive software library for efficient processing of structured genome annotations. IEEE/ACM Trans Computational Biology and Bioinformatics10: 645-656. https://doi.org/10.1109/TCBB.2013.68.

Guo, Q., Pan, Y., Dai, W., Guo, F., Zeng, T., Chen, W., Mi, Y., Zhang, Y., Shi, S., Jiang, W., Cai, H., Wu, B., Zhou, Y., Wang, Y., Yang, C., Shi, X., Yan, X., Chen, J., Cai, C., Yang, J., Xu, X., Gu, Y., Dong, Li, Q. 2025. A near-complete genome assembly of the bearded dragon *Pogona vitticeps* provides insights into the origin of *Pogona* sex chromosomes. GigaScience, in press.

Hanrahan, B.J., Alreja, K., Reis, A.L.M., Chang, J.K., Dissanayake, D.S.B., Edwards, R., Bertozzi, T., Hammond, J.M., O'Meally, D., Deveson, I.W., Georges A., Waters, P., Patel, H.R. 2025. A genome assembly and annotation for the Australian alpine skink *Bassiana duperreyi* using long-read technologies. G3: Genes | Genomes | Genetics, 15: jkaf046. http://doi.org/10.1093/g3journal/jkaf043.

Holleley, C.E., O'Meally, D., Sarre, S.D., Graves, J.A.M., Ezaz, T., Matsubara, K., Azad, B., Zhang, X., Georges, A. 2015. Sex reversal triggers the rapid transition from genetic to temperature-dependent sex. Nature 523: 79-82. https://doi.org/10.1038/nature14574.

Infante, C.R., Rasys, A.M., Menke, D.B. (2018). Appendages and gene regulatory networks: Lessons from the limbless. Genesis 56: e23078. https://doi.org/10.1002/dvg.23078.

Jeffries, D.L., Mee, J.A., Peichel, C.L. 2022. Identification of a candidate sex determination gene in *Culaea inconstans* suggests convergent recruitment of an *Amh* duplicate in two lineages of stickleback. Journal of Evolutionary Biology 35: 1683–1694. https://doi.org/10.1111/jeb.14034.

Kamiya, T., Kai, W., Tasumi, S., Oka, A., Matsunaga, T., Mizuno, N., Fujita, M., Suetake, H., Suzuki, S., Hosoya, S., Tohari, S., Brenner, S., Miyadai, T., Venkatesh, B., Suzuki, Y., Kikuchi, K. 2012. A trans-species missense SNP in Amhr2 is associated with sex determination in the tiger pufferfish, *Takifugu rubripes* (fugu). PLoS Genetics 8:e1002798. https://doi.org/10.1371/journal.pgen.1002798.

Kolmogorov, M., Yuan, J., Lin, Y., Pevzner, P. 2019. Assembly of long error-prone reads using repeat graphs. Nature Biotechnology 37: 540-546. https://doi.org/10.1038/s41587-019-0072-8.

Koopman, P., Gubbay, J., Vivian, N., Goodfellow, P., Lovell-Badge, R. 1991. Male development of chromosomally female mice transgenic for *Sry* gene. Nature 351: 117–21. https://doi.org/10.1038/351117a0.

Lambeth, L.S., Raymond, C.S., Roeszler, K.N., Kuroiwa, A., Nakata, T., Zarkower, D., Smith, C.A. 2014. Over-expression of DMRT1 induces the male pathway in embryonic chicken gonads. Developmental Biology 389: 160-172. https://doi.org/10.1016/j.ydbio.2014.02.012.

Lawniczak, M.K.N., Durbin, R., Flicek, P., +41 , Richards, S. 2022. Standards recommendations for the Earth BioGenome Project. Proceedings of the National Academy of Sciences (USA) 119: e2115639118. https://doi.org/10.1073/pnas.2115639118.

Li, H. 2018. Minimap2: pairwise alignment for nucleotide sequences, *Bioinformatics* 34: 3094–3100. https://doi.org/10.1093/bioinformatics/bty191.

Li, M., Sun, Y., Zhao, J., Shi, H., Zeng, S., Ye, K., Jiang, D., Zhou, L., Sun, L., Tao, W., Nagahama, Y., Kocher, T.D., Wang, D. 2015. A tandem duplicate of anti-Müllerian hormone with a missense SNP on the Y chromosome is essential for male sex determination in Nile Tilapia, *Oreochromis niloticus*. PLoS Genetics 11: e1005678. https://doi.org/10.1371/journal.pgen.1005678.

Liao, Y., Smyth, G.K., Shi, W. 2013. The Subread aligner: fast, accurate and scalable read mapping by seed-and-vote. Nucleic Acids Research 41: e108. https://doi.org/10.1093/nar/gkt214.

McDonald, E., Whibley, A., Waters, P.D., Patel, H., Edwards, R.J., Ganley, A.R.D. 2024. Origin and maintenance of large ribosomal RNA gene repeat size in mammals. Genetics 228:iyae121, https://doi.org/10.1093/genetics/iyae121.

Manni, M., Berkeley, M.R., Seppey, M., Zdobnov, E.M. 2021. BUSCO: Assessing genomic data quality and beyond. Current Protocols https://doi.org/10.1002/cpz1.323. https://doi.org/10.1002/cpz1.323.

Marco-Sola, S., Sammeth, M., Guigó, R., Ribeca, P. 2012. The GEM mapper: Fast, accurate and versatile alignment by filtration. Nature Methods 9: 1185–1188. https://doi.org/10.1038/nmeth.2221

Miller, S.A., Dykes, D.D., Polesky, H.F. 1998. A simple salting out procedure for extracting DNA from human nucleated cells. Nucleic Acids Research 16: 1215. https://doi.org/10.1093/nar/16.3.1215.

Mokhtaridoost, M., Chalmers, J.J., Soleimanpoor, M., McMurray, B.J., Lato, D.F., Nguyen, S.C., Musienko, V., Nash, J.O., Espeso-Gil, S., Ahmed, S., Delfosse, K., Browning, J.W.L., Barutcu, A.R., Wilson, M.D., Liehr, T., Shlien, S., Aref, A., Joyce, E.F., Weise, A., Maass, P.G. 2024. Inter-chromosomal contacts demarcate genome topology along a spatial gradient. Nature Communications 15: 9813. https://doi.org/10.1038/s41467-024-53983-y.

Nagashima, S., Yamaguchi, S.T., Zhou, Z., Norimoto, H. 2024. Transient cooling resets circadian rhythms of locomotor activity in lizards. Journal of Biological Rhythms 39: 607-613. doi:10.1177/07487304241273190.

Nakamoto, M., Uchino, T., Koshimizu, E., Kuchiishi, Y., Sekiguchi, R., Wang, L., Sudo, R., Endo, M., Guiguen, Y., Schartl, M., Postlethwait, J.H., Sakamoto, T. 2021. A Y-linked anti-Müllerian hormone type-II receptor is the sex-determining gene in ayu, *Plecoglossus altivelis*. PLoS Genetics 17: e1009705. https://doi.org/10.1371/journal.pgen.1009705

Ollonen, J., da Silva, F.O., Mahlow, K., Di-Poil, N. 2018. Skull development, ossification pattern, and adult shape in the emerging lizard model organism *Pogona vitticeps*: A comparative analysis with other Squamates. Frontiers in Physiology 2018:00278. https://doi.org/10.3389/fphys.2018.00278.

Pasquesi, G.I.M., Adams, R.H., Card, D.C., Schield, D.R., Corbin, A.B., Perry, B.W., Reyes-Velasco, J., Ruggiero, R.P., Vandewege, M.W., Shortt, J.A., Castoe, T.A. 2018. Squamate reptiles challenge paradigms of genomic repeat element evolution set by birds and mammals. Nature Communications 9: 2774. https://doi.org/10.1038/s41467-018-05279-1.

Patel HR; Alreja K; M Reis AL; Chang JK; Chew ZA; Jung H; Hammond JM; Deveson IW; Ruiz-Herrera A; Marin-Gual L; Holleley CE; Zhang X; Lister NC; Whiteley S; Xiong L; B Dissanayake DS; Waters PD; Georges A (2025): Supporting data for "A near telomere-to-telomere phased genome assembly and annotation for the Australian central bearded dragon *Pogona vitticeps*" GigaScience Database. https://doi.org/10.5524/102730

Quinn, A.E., Georges, A., Sarre, S.D., Guarino, F., Ezaz, T., Graves, J.A.M. 2007. Temperature sex reversal implies sex gene dosage in a reptile. Science 316: 411. https://doi.org/10.1126/science.1135925.

Quinn, A.E., Ezaz, T., Sarre, S.D., Graves, J.A.M., Georges, A. 2010. Extension, single-locus conversion and physical mapping of sex chromosome sequences identify the Z microchromosome and pseudo-autosomal region in a dragon lizard, *Pogona vitticeps*. Heredity 104: 410-417. https://doi.org/10.1038/hdy.2009.133.

Ramírez, F., Bhardwaj, V., Arrigoni, L., Lam, K. C., Grüning, B.A., Villaveces, J., Habermann, B., Akhtar, A., Manke, T. 2018. High-resolution TADs reveal DNA sequences

underlying genome organization in flies. Nature Communications 9: 189. https://doi.org/10.1038/s41467-017-02525-w.

Razmadze, D., Salomies, L., Di-Poï, N. 2024. Squamates as a model to understand key dental features of vertebrates. Developmental Biology 516: 1-19. https://doi.org/10.1016/j.ydbio.2024.07.011.

Rhie, A., Walenz, B.P., Koren, S., Phillippy, A.M. 2020. Merqury: reference-free quality, completeness, and phasing assessment for genome assemblies. Genome Biology 21: 245. https://doi.org/10.1186/s13059-020-02134-9.

Rodrigues, N., Vuille, Y., Brelsford, A., Merilä, J., Perrin, N. 2016. The genetic contribution to sex determination and number of sex chromosomes vary among populations of common frogs (*Rana temporaria*). Heredity 117: 25–32. https://doi.org/10.1038/hdy.2016.22.

Serra, F., Baù, D., Goodstadt, M., Castillo, D., Filion, G.J., Marti-Renom, M.A. 2017. Automatic analysis and 3D-modelling of Hi-C data using TADbit reveals structural features of the fly chromatin colors. PLoS Computational Biology 13: e1005665. https://doi.org/10.1371/journal.pcbi.1005665.

Sinclair, A.H., Berta, P., Palmer, M.S., Hawkins, J.R., Griffiths, B.L., Smith, M.J., Foster, J.W., Frischauf, A.M., Lovell-Badge, R., Goodfellow, P.N. 1990. A gene from the human sex-determining region encodes a protein with homology to a conserved DNA-binding motif. Nature 346: 240–4. https://doi.org/10.1038/346240a0.

Smit, A..FA., Hubley, R. 2008-2015. RepeatModeler Open-1.0. <http://www.repeatmasker.org>. Last accessed 1-Jun-2025.

Smit, A.F.A., Hubley, R., Green, P. 2013-2015. RepeatMasker Open-4.0. http://www.repeatmasker.org. Last accessed 1-Jun-2025.

Smith, C., Roeszler, K., Ohnesorg, T., Cummins, D.M., Farlie, P.G., Doran, T.J., Sinclair, A.H. 2009. The avian Z-linked gene *DMRT1* is required for male sex determination in the chicken. Nature 461: 267–271. https://doi.org/10.1038/nature08298.

Song, W., Xie, Y., Sun, M., Li, X., Fitzpatrick, C.K., Vaux, F., O'Malley, K.G., Zhang, Q., Qi, J., He, Y. 2021. A duplicated *amh* is the master sex-determining gene for *Sebastes*

rockfish in the Northwest Pacific. Open Biology 11: 210063. doi: http://doi.org/10.1098/rsob.210063.

Stanke, M., Morgenstern, B. 2005. AUGUSTUS: a web server for gene prediction in eukaryotes that allows user-defined constraints. Nucleic Acids Research 33: W465-7. https://doi.org/10.1093/nar/gki458.

Vasimuddin, M., Misra, S., Li, H., Aluru, S. 2019. Efficient Architecture-Aware Acceleration of BWA-MEM for Multicore Systems. IEEE Parallel and Distributed Processing Symposium (IPDPS), 2019. https://doi.org/10.1109/IPDPS.2019.00041.

Wagner, S., Whiteley, S.L., Castelli, M., Patel, H.R., Deveson, I.W., Blackburn, J., Holleley, C.E., Marshall Graves, J.A., Georges, A. 2023. Gene expression of male pathway genes *sox9* and *amh* during early sex differentiation in a reptile departs from the classical amniote model. BMC Genomics 24: 243. https://doi.org/10.1186/s12864-023-09334-0.

Waters, P.D., Patel, H.R.,Ruiz-Herrera, A., Álvarez-González, L., Lister, N.C., Simakov, O., Ezaz, T., Kaur, P., Frere, C., Grützner, F., Georges, A., Marshall Graves, J.A. 2021. Microchromosomes are building blocks of bird, reptile and mammal chromosomes. Proceedings of the National Academy of Sciences (USA) 118: e2112494118. https://doi.org/10.1073/pnas.2112494118.

Whiteley, S.L., Holleley, C.E., Blackburn, J., Deveson, I.W., Wagner, S., Graves, J.A.M., Georges, A. 2021. Two transcriptionally distinct pathways drive female development in a reptile with genetic sex determination and temperature induced sex reversal. PLoS Genetics 17: e1009465. https://doi.org/10.1371/journal.pgen.1009465.

Whiteley, S.L., Holleley, C.E. and Georges, A. 2022. Developmental dynamics of sex reprogramming by high incubation temperatures in a dragon lizard. BMC Genomics 23: 322. https://doi.org/10.1186/s12864-022-08544-2.

Witten J.G. 1983. Some karyotypes of Australian agamids (Reptilia: Lacertilia). Australian Journal of Zoology 31: 533-540. https://doi.org/10.1071/ZO9830533.

Young, M.J., O'Meally, D., Sarre, S.D., Georges, A., Ezaz, T. 2013. Molecular cytogenetic map of the central bearded dragon *Pogona vitticeps* (Squamata: Agamidae). Chromosome

Research 21: 361-374. https://doi.org/10.1007/s10577-013-9362-z.

Zhang, X., Wagner, S., Deakin, J.E., Holleley, C.E., Matsubara, K., Deveson, I.W., Li, Z., Wang, C., O'Meally, D., Edwards, M., Patel, H.R., Ezaz, T., Marshall Graves, J.M., Georges, A. 2022. Sex-specific splicing of Z- and W-borne *nr5a1* alleles suggests sex determination is controlled by chromosome conformation. Proceedings of the National Academy of Sciences (USA) 119: e2116475119. https://doi.org/10.1073/pnas.211647511.

Zhang, Y., Chu, J., Cheng, H., Li, H. 2023. De novo reconstruction of satellite repeat units from sequence data. Genome Research 33: 1994-2001 https://doi.org/10.1101/gr.278005.123.

Zhou, C., McCarthy, S.A., Durbin, R. 2023. YaHS: yet another Hi-C scaffolding tool. *Bioinformatics*. 39: btac808. https://doi.org/10.1093/bioinformatics/btac808.

Zhou, Y., Shearwin-Whyatt, L., Li, J., Song, Z., Hayakawa, T., Stevens, D., Fenelon, J.C., Peel, E., Cheng, Y., Pajpach, F., Bradley, N., Suzuki, H., Nikaido, M., Damas, J., Daish, T., Perry, T., Zhu, Z., Geng, Y., Rhie, A., Sims, Y., Wood, J., Haase, B., Mountcastle, J., Fedrigo, O., Li, Q., Yang, H., Wang, J., Johnston, S.D., Phillippy, A.M., Howe, K., Jarvis, E.D., Ryder, O.A., Kaessmann, H., Donnelly, P., Korlach, J., Lewin, H.A., Graves, J., Belov, K., Renfree, M.B., Grutzner, F., Zhou, Q., Zhang, G. 2021. Platypus and echidna genomes reveal mammalian biology and evolution. Nature 592: 756–762 https://doi.org/10.1038/s41586-020-03039-0.

Click here to access/download
Supplementary Material
Pogona_genome_Supplementary-Revision.docx
