# Supplementary material for: A near telomere-to-telomere phased genome assembly and annotation for the Australian central bearded dragon Pogona vitticeps
Source: Gigascience. 2025 Aug 19;14:giaf085. doi: 10.1093/gigascience/giaf085 (PMC12360841; doi:10.1093/gigascience/giaf085)
Supplement: giaf085_Authors_Response_To_Reviewer_Comments_Original_Submission [file giaf085_authors_response_to_reviewer_comments_original_submission.pdf]

Author's Response To Reviewer Comments

Recommendation: This work represents a major advancement in reptilian genomics. Addressing the above issues—particularl
Reviewer #1
The authors de novo assembled a telomere to telomere phased genome assembly of the Australian central bearded dragon Po
The phased assembly of sex chromosomes (Z/W) and identification of candidate sex-determining genes (Amh, Amhr2, and N
We are grateful for this assessment.
Major concern:
1. The description of read depth had errors at lines 401-402, such as 60.6x. In addition, "4 x promethION", "2x150 bp" were
Agreed. We have corrected the values in the text (Lines 412:413) to match the values in Table 1.
2. There are errors in the citation format of the journal references, such as the absence of punctuation "."marks between the
We have checked the references for consistency and made the necessary changes.
3. The script "calculateGC.py and processtrftelo.py" (lines 242 and 245) are mentioned without code availability or paramete
All scripts are available at https://github.com/kango2/ausarg. Please See Line 194-195.

4. The inconsistent use of "Gb" and "Gbp" is observed; it is recommended to adopt a unified description.
Agreed. We have corrected in the manuscript to use Gbp consistently.
5. Units were missing in the descriptions in multiple places in Table 1 and 2, such as the unit for "Total Bases" and "Assembly
Agreed. We have corrected this in Table 2. Table 1 did not require any change.
6. At lines 683-687, the conclusion that Amh/Amhr2 are sex-determining genes relies solely on positional evidence. Discuss t
Agreed. We have added text to this effect on lines 716-722 and added additional citations to the reference list:
Added Lines 716-722. This will be a fruitful area for future functional studies based on upregulation and knockout technologie
and to establish Drmt1 on the Z chromosomes of birds as the master sex determining gene acting though dosage (Smith et a
7. There were errors in "Vasimuddin et al. 2019" (line 238) and "Danecek et al. 2021" (line 239). Please check all the other f
Corrected.
8. At lines 476-481, BAC mappings are cited as validation but lack visual evidence (e.g., alignment plots in figures or supplem
Clarification: Figure 4 includes visual representation of BAC alignments in their physical positions as red diamonds. The refere
Reviewer #2
Patel et al. present a genome assembly of the bearded dragon Pogona vitticeps a lizard species that is widely distributed as a
ONT long reads, Hi-C and suitable bioinformatic tools.
Again, we are grateful for these kind comments.
I have to admit that I have recently been reviewing a similar manuscript for Gigascience
(https://aus01.safelinks.protection.outlook.com/?url=https%3A%2F%2Fwww.biorxiv.org%2Fcontent%2F10.1101%2F2024.0
cGkiOnRydWUsIlYiOiIwLjAuMDAwMCIsIlAiOiJXaW4zMiIsIkFOIjoiTWFpbCIsIldUIjoyfQ%3D%3D%7C0%7C%7C%7C&sdata=tzA
done by short read coverage analysis. One of my major comments was that this approach lacked a true assembly of the W-c
accuracy etc.
The two manuscripts are highly complementary and I think they should be published, if possible, in the very same issue of Gi
Yes, that is our intention – to have a simultaneous publication announcing the discovery of AMH on the Z chromosome but ab
Still there are some minor points that need improvement for the current manuscript:
1. Why do you leave the Z and W splitted into PAR, Z- and W-specific scaffolds and do not assemble the full-length chromoso

The HiC used in the YAHS assembly did not resolve these breaks between the sex chromosome scaffolds 17 and 18 and the p
"The Z and W specific sex chromosome scaffolds were identified as 18 and 17, respectively. These represent the non-recomb
Mitochondrial assembly: from ONT only (L. 307), please do a consensus correction with illumina data, or at least show that th
We have now included the assessment of consensus accuracy using the Illumina data as suggested. Please see Lines 313-317
Genome annotation: show BUSCO scores for annotated proteins (do they fit to BUSCO performed on the whole genome?).
Agreed. We have now included BUSCO scores for annotated proteins in the manuscript (Lines 386-387 methods, lines 671-67
If possible, compare to results of the NCBI RefSeq annotation (is it already available?).
The NCBI RefSeq annotation is not available. They will become available upon the release of the genome assembly post-publi
In this regard please explain the relatively low mapping rates (L. 647) of RNAseq to the annotated sequences.
We have added a sentence to discuss the implication of the low-mapping rates on lines 678-681):
Relatively lower mapping rates of RNAseq data can be attributed to incomplete annotations of untranslated regions and the p

Could you provide some expression data for the Z-specific Amh and AmhR2? Is it differentially expressed in testis/ovary (afte
We have addressed this on lines 716-722 as outlined above. This work is underway and will be the subject of a future publica

Table1, could you show results for the two different ONT library types (ligation vs. ultralong kit). It seems the overall yield wa
The reviewer may have missed this, but Table S4 contain details of individual runs. Lines 396 covers the speculation.
I think assembly statistics (Table2) should also contain contig N50 length as an additional value to show the high continuity c
We have added lines 445-447 to describe contig N50, N90 values. Table 2 is not altered because values presented there are a
L. 488: "48.36 (1 error in 146kb)", I think something is wrong here. Q48.36 would be 1 error in 68.5kb. I would suggest to r
Agreed. Thank you for spotting this error in our calculations. We misinterpreted the error probability value of (0.000014588),
The high consensus accuracy is one selling point compared to the competitor's assembly.
L. 490: "Individual haplotypes were 85.5% complete…". Explain why you are confident that the haplotypes are more complet
We provide justification for this on lines 514-516 where we say:
"This shows that assembly completeness metrics for a single haplotype assembly measured using k-mers can be understated

Editorial Manager®

Cookie Preference Center

We use cookies which are necessary to make our site work. We may also use additional cookies to analyze, improve and personalize our content and your digital experience. For more information, see our Cookie Policy.

You may choose not to allow some types of cookies. However, blocking some types may impact your experience of our site and the services we are able to offer. See the different category headings below to find out more or change your settings.

Allow all

Manage Consent Preferences

Strictly Necessary Cookies

Always active

These cookies are necessary for the website to function and cannot be switched off in our systems. They are usually only set in response to actions made by you which amount to a request for services, such as setting your privacy preferences, logging in or filling in forms. You can set your browser to block or alert you about these cookies, but some parts of the site will not then work.

Functional Cookies

These cookies enable the website to provide enhanced functionality and personalisation. They may be set by us or by third party providers whose services we have added to our pages. If you do not allow these cookies then some or all of these services may not function properly.

Performance Cookies

These cookies allow us to count visits and traffic sources so we can measure and improve the performance of our site. They help us to know which pages are the most and least popular and see how visitors move around the site.

Targeting Cookies

These cookies may be set through our site by our advertising partners. They may be used by those companies to build a profile of your interests and show you relevant adverts on other sites. If you do not allow these cookies, you will experience less targeted advertising.

Cookie List

Clear

Apply Cancel

Consent Leg.Interest

Confirm my choices

Powered by onetrus
