# Supplementary material for: A near telomere-to-telomere phased genome assembly and annotation for the Australian central bearded dragon Pogona vitticeps
Source: Gigascience. 2025 Aug 19;14:giaf085. doi: 10.1093/gigascience/giaf085 (PMC12360841; doi:10.1093/gigascience/giaf085)
Supplement: giaf085_Reviewer_1_Report_Original_Submission — Yuan Li -- 5/15/2025 [file giaf085_reviewer_1_report_original_submission.pdf]

**Reviewer Report**

**Title: A near-telomere to telomere phased genome assembly and annotation for the Australian central bearded dragon Pogona vitticeps**

**Version: Original Submission** **Date:** 5/15/2025

**Reviewer name: Yuan Li**

**Reviewer Comments to Author:**

The authors de novo assembled a telomere to telomere phased genome assembly of the Australian central bearded dragon Pogona vitticeps, using PacBio HiFi, ONT, HiC, and Illumina sequencing platforms. The assembly achieves remarkable contiguity (scaffold N50: 266.2 Mb) and completeness (97.9% BUSCO score), surpassing Earth Biogenome Project standards. The phased assembly of sex chromosomes (Z/W) and identification of candidate sex-determining genes (Amh, Amhr2, and Nr5a1) provide valuable insights into reptilian sex determination. Overall, the study is well-executed and provides a valuable resource for comparative genomics and reproductive biology.

Major concern:

1.The description of read depth had errors at lines 401-402, such as 60.6x. In addition, "4 x promethION", "2x150 bp" were should be revised and please check and revise all the similar description in the manuscript.

2.There are errors in the citation format of the journal references, such as the absence of punctuation "."marks between the title name and the journal name at lines 1005-1009, mixing abbreviations (e.g., "PNAS" vs. "Proceedings of the National Academy of Sciences USA") (lines 988-990, 1005-1009). Please check carefully the format of all references.

3.The script "calculateGC.py and processtrftelo.py" (lines 242 and 245) are mentioned without code availability or parameter details. Provide effective links or repository access.

4.The inconsistent use of "Gb" and "Gbp" is observed; it is recommended to adopt a unified description.

5.Units were missing in the descriptions in multiple places in Table 1 and 2, such as the unit for "Total Bases" and "Assembly length"; please include them.

6.At lines 683-687, the conclusion that Amh/Amhr2 are sex-determining genes relies solely on positional evidence. Discuss the need for functional studies (e.g., CRISPR knockouts) to strengthen claims.

7.There were errors in "Vasimuddin et al. 2019" (line 238) and "Danecek et al. 2021" (line 239). Please check all the other formats of references.

8.At lines 476-481, BAC mappings are cited as validation but lack visual evidence (e.g., alignment plots in figures or supplements). Please verify the accuracy of Figure 7 at line 478, as it does not correspond with the description.

**Methods**

Are the methods appropriate to the aims of the study, are they well described, and are necessary controls included? Choose an item.

**Conclusions**

Are the conclusions adequately supported by the data shown? Choose an item.

**Reporting Standards**

Does the manuscript adhere to the journal's guidelines on minimum standards of reporting? Choose an item.

Choose an item.

**Statistics**

Are you able to assess all statistics in the manuscript, including the appropriateness of statistical tests used? Choose an item.

**Quality of Written English**

Please indicate the quality of language in the manuscript: Choose an item.

**Declaration of Competing Interests**

Please complete a declaration of competing interests, considering the following questions:

- Have you in the past five years received reimbursements, fees, funding, or salary from an organisation that may in any way gain or lose financially from the publication of this manuscript, either now or in the future?
- Do you hold any stocks or shares in an organisation that may in any way gain or lose financially from the publication of this manuscript, either now or in the future?
- Do you hold or are you currently applying for any patents relating to the content of the manuscript?
- Have you received reimbursements, fees, funding, or salary from an organization that holds or has applied for patents relating to the content of the manuscript?
- Do you have any other financial competing interests?
- Do you have any non-financial competing interests in relation to this paper?

If you can answer no to all of the above, write 'I declare that I have no competing interests' below. If your reply is yes to any, please give details below.

I declare that I have no competing interests

I agree to the open peer review policy of the journal. I understand that my name will be included on my report to the authors and, if the manuscript is accepted for publication, my named report including any attachments I upload will be posted on the website along with the authors' responses. I agree for my

report to be made available under an Open Access Creative Commons CC-BY license (http://creativecommons.org/licenses/by/4.0/). I understand that any comments which I do not wish to be included in my named report can be included as confidential comments to the editors, which will not be published.

Choose an item.

To further support our reviewers, we have joined with Publons, where you can gain additional credit to further highlight your hard work (see: https://publons.com/journal/530/gigascience). On publication of this paper, your review will be automatically added to Publons, you can then choose whether or not to claim your Publons credit. I understand this statement.

Yes Choose an item.
