# Supplementary material for: A near telomere-to-telomere phased genome assembly and annotation for the Australian central bearded dragon Pogona vitticeps
Source: Gigascience. 2025 Aug 19;14:giaf085. doi: 10.1093/gigascience/giaf085 (PMC12360841; doi:10.1093/gigascience/giaf085)
Supplement: giaf085_Reviewer_2_Report_Original_Submission — Heiner Kuhl -- 5/31/2025 [file giaf085_reviewer_2_report_original_submission.pdf]

Reviewer Report

Title: A near-telomere to telomere phased genome assembly and annotation for the Australian central bearded dragon Pogona vitticeps

Version: Original Submission **Date:** 5/31/2025

Reviewer name: Heiner Kuhl

Reviewer Comments to Author:

Patel et al. present a genome assembly of the bearded dragon Pogona vitticeps a lizard species that is widely distributed as a pet and known for its interesting sex-determination, which may switch from genetic sex-determination (ZW) to temperature dependent sex-reversal. The methods chosen to assemble the genome are very state-of-the-art including HIFI and ONT long reads, Hi-C and suitable bioinformatic tools.
I have to admit that I have recently been reviewing a similar manuscript for Gigascience (https://www.biorxiv.org/content/10.1101/2024.09.05.611321v1), where a female ZZ P. vitticeps had been sequenced/assembled from long read data of a different nanopore technology and analyses of the ZW-chromosome was done by short read coverage analysis. One of my major comments was that this approach lacked a true assembly of the W-chromosome. Thus, I am happy to see that the assembly of the W-specific region has been achieved here and the sequencing technologies used might even improve the assembly quality over the ZZ assembly in terms of phasing, consensus accuracy etc.
The two manuscripts are highly complementary and I think they should be published, if possible, in the very same issue of Gigascience. Surely both groups have invested a lot of efforts.
(Reading L. 685, I just have realized that this seems to be the intention of the journal and I very much support this idea.)
Still there are some minor points that need improvement for the current manuscript:
Why do you leave the Z and W splitted into PAR, Z- and W-specific scaffolds and do not assemble the full-length chromosomes (L. 676)? Would the Hi-C data not support that?
Mitochondrial assembly: from ONT only (L. 307), please do a consensus correction with illumina data, or at least show that the MT assembly has a high consensus accuracy (Q40-Q50).
Genome annotation: show BUSCO scores for annotated proteins (do they fit to BUSCO performed on the whole genome?). If possible, compare to results of the NCBI RefSeq annotation (is it already available?). In this regard please explain the relatively low mapping rates (L. 647) of RNAseq to the annotated sequences.
Could you provide some expression data for the Z-specific Amh and AmhR2? Is it differentially expressed in testis/ovary (after correction for copy number)?
Table1, could you show results for the two different ONT library types (ligation vs. ultralong kit). It seems the overall yield was low (5 cells -> 100Gb), any speculation why?
I think assembly statistics (Table2) should also contain contig N50 length as an additional value to show the high continuity of the assembly.
L. 488: "48.36 (1 error in 146kb)", I think something is wrong here. Q48.36 would be 1 error in 68.5kb. I

would suggest to re-check these values and incorporate them in Table2. The high consensus accuracy is one selling point compared to the competitor's assembly.
L. 490: "Individual haplotypes were 85.5% complete…". Explain why you are confident that the haplotypes are more complete than the Merqury results suggest (just one sentence).

Methods

Are the methods appropriate to the aims of the study, are they well described, and are necessary controls included? Choose an item.

Conclusions

Are the conclusions adequately supported by the data shown? Choose an item.

Reporting Standards

Does the manuscript adhere to the journal's guidelines on minimum standards of reporting? Choose an item.

Choose an item.

Statistics

Are you able to assess all statistics in the manuscript, including the appropriateness of statistical tests used? Choose an item.

Quality of Written English

Please indicate the quality of language in the manuscript: Choose an item.

Declaration of Competing Interests

Please complete a declaration of competing interests, considering the following questions:

- Have you in the past five years received reimbursements, fees, funding, or salary from an organisation that may in any way gain or lose financially from the publication of this manuscript, either now or in the future?
- Do you hold any stocks or shares in an organisation that may in any way gain or lose financially from the publication of this manuscript, either now or in the future?
- Do you hold or are you currently applying for any patents relating to the content of the manuscript?
- Have you received reimbursements, fees, funding, or salary from an organization that holds or has applied for patents relating to the content of the manuscript?
- Do you have any other financial competing interests?

- Do you have any non-financial competing interests in relation to this paper?

If you can answer no to all of the above, write 'I declare that I have no competing interests' below. If your reply is yes to any, please give details below.

I declare that I have no competing interests

I agree to the open peer review policy of the journal. I understand that my name will be included on my report to the authors and, if the manuscript is accepted for publication, my named report including any attachments I upload will be posted on the website along with the authors' responses. I agree for my report to be made available under an Open Access Creative Commons CC-BY license (http://creativecommons.org/licenses/by/4.0/). I understand that any comments which I do not wish to be included in my named report can be included as confidential comments to the editors, which will not be published.

Choose an item.

To further support our reviewers, we have joined with Publons, where you can gain additional credit to further highlight your hard work (see: https://publons.com/journal/530/gigascience). On publication of this paper, your review will be automatically added to Publons, you can then choose whether or not to claim your Publons credit. I understand this statement.

Yes Choose an item.
